# Design, methods, and participant characteristics of the Impact of Personal Genomics (PGen) Study, a prospective cohort study of direct-to-consumer personal genomic testing customers

**DOI:** 10.1186/s13073-014-0096-0

**Published:** 2014-12-03

**Authors:** Deanna Alexis Carere, Mick P Couper, Scott D Crawford, Sarah S Kalia, Jake R Duggan, Tanya A Moreno, Joanna L Mountain, J Scott Roberts, Robert C Green, for the PGen Study Group

**Affiliations:** Department of Epidemiology, Program in Genetic Epidemiology and Statistical Genetics, Harvard School of Public Health, Boston, MA 02115 USA; Department of Medicine, Division of Genetics, Brigham and Women’s Hospital, EC Alumnae Building, Suite 301, 41 Avenue Louis Pasteur, Boston, MA 02115 USA; Survey Research Center, University of Michigan Institute for Social Research, Ann Arbor, MI 48106 USA; Survey Sciences Group, LLC, Ann Arbor, MI 48108 USA; Pathway Genomics, San Diego, CA 92121 USA; 23andMe Inc, Mountain View, CA 94043 USA; Department of Health Behavior and Health Education, University of Michigan School of Public Health, Ann Arbor, MI 48104 USA; Harvard Medical School, EC Alumnae Building, Suite 301, 41 Avenue Louis Pasteur, Boston, MA 02115 USA; Partners Personalized Medicine, EC Alumnae Building, Suite 301, 41 Avenue Louis Pasteur, Boston, MA 02115 USA

## Abstract

Designed in collaboration with 23andMe and Pathway Genomics, the Impact of Personal Genomics (PGen) Study serves as a model for academic-industry partnership and provides a longitudinal dataset for studying psychosocial, behavioral, and health outcomes related to direct-to-consumer personal genomic testing (PGT). Web-based surveys administered at three time points, and linked to individual-level PGT results, provide data on 1,464 PGT customers, of which 71% completed each follow-up survey and 64% completed all three surveys. The cohort includes 15.7% individuals of non-white ethnicity, and encompasses a range of income, education, and health levels. Over 90% of participants agreed to re-contact for future research.

**Electronic supplementary material:**

The online version of this article (doi:10.1186/s13073-014-0096-0) contains supplementary material, which is available to authorized users.

## Background

Nearly a decade after being introduced to the market, commercial genomic profiling services, through which consumers can independently obtain analysis and interpretation of their genetic code, continue to fuel debate among stakeholders regarding their clinical validity and utility. Commercial offerings that were once covered by the term ‘direct-to-consumer’ (DTC) genetic testing, owing to their ‘pure’ model of direct access without clinician involvement, have since diversified to encompass a range of personal genomic testing (PGT) models with varying levels of clinician involvement both before and following testing [1]; nonetheless, the original criticisms of the industry persist. Advocates argue that by facilitating direct access to an individual’s own genetic information, PGT can promote democratization and autonomy in health-related decision-making [2,3], and enable individuals to control the privacy and use of their personal genetic data [4]. Critics caution that risk information from PGT could be misunderstood and lead to anxiety, inappropriate self-treatment or unnecessary utilization of health care services [5,6].

The call for empirical data on the impact of PGT services has been widespread [7-10] and has resulted in studies addressing such topics as public awareness [11-13], consumer interest and motivations [14], results comprehension [15,16], and physician awareness and preparedness [17]. Only a few studies, however, have involved *actual* PGT customers [18], and of these, many have been limited by low response rates [19,20], small sample sizes [19,21,22], an absence of baseline data collection [19,23], service models that differ from current commercial models [22,24-26], and an inability to link participants’ responses to their PGT results [20,23]. Research interest in PGT and its role in public health was renewed in November 2013, when the United States Food and Drug Administration (FDA) sent a Warning Letter to 23andMe, Inc. ordering it to cease marketing of its health-related PGT services until it received medical device authorization [27].

Designing an academic study of the impact of PGT presents a considerable challenge. Without collaboration between academia and industry, customers would be difficult to identify and contact, and specific details of the PGT service could be inaccessible to investigators. The Impact of Personal Genomics (PGen) Study is a longitudinal study of PGT customers that was developed in partnership with two PGT companies, 23andMe, Inc. [28] (23andMe) and Pathway Genomics [29] (Pathway), to evaluate customer motivations for purchasing PGT and the effect of PGT risk information on psychological status, risk perceptions and health behaviors. We have previously reported on how we negotiated the ethical issues associated with developing and maintaining a research partnership between academia and industry in planning the PGen Study [30]. Here, we present the surveys themselves, and describe the protocol design and data collection process, our survey response and retention rates, and the demographics and self-reported health status of the PGen Study cohort.

## Methods

### Overview

**Figure 1:**
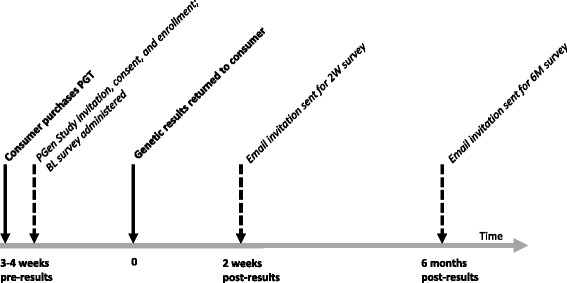
**Timing of personal genomic testing (PGT) and survey data collection in the PGen Study.** Steps of the PGT process are indicated by solid arrows and bold text; data collection points are indicated by dashed arrows and italicized text. BL, baseline; 2 W, 2 week; 6 M, 6 month.

The PGen Study was designed as a longitudinal series of surveys (Figure 1) to measure the PGT experience at three time points: baseline, after customers had ordered PGT, but before they received their results (BL); approximately 2 weeks after receiving results (2 W); and approximately 6 months after receiving results (6 M).

Planning for the PGen Study was initiated in 2009 by academic researchers at Harvard Medical School in Boston, Massachusetts and the University of Michigan School of Public Health in Ann Arbor, Michigan. Industry collaborators were sought from among the major PGT companies operating at the time, and two (23andMe and Pathway) agreed to participate. Details of how this partnership was established, including a discussion of the ways in which its inherent ethical challenges were addressed during the planning phase of the PGen Study, have been published previously [30]. Funding was received from the National Human Genomic Research Institute in 2010, and approval was obtained from Partners Human Research Committee and the University of Michigan School of Public Health Institutional Review Board.

### Survey design

Academic and industry scientists participated in a two-day retreat, followed by regular conference calls, to develop an initial set of survey items. Questions were drawn from validated measures of psychological states [31-33], health behaviors [34,35], and numeracy [36]; and from previous studies of PGT [36-40], genetics knowledge [41,42], and numeracy [43]. Academic and company scientists, along with expert consultants, were invited to propose additional established or novel survey items, which were then reviewed by the entire research team. Data collection interests of all parties were considered and balanced throughout this process, in consultation with bioethics advisors.

Through pilot testing with cognitive interview techniques, we refined the wording of certain questions, and added or deleted questions to improve the length and overall flow of the survey. The BL, 2 W, and 6 M surveys were each designed to be completed in no more than 30 minutes to minimize respondent burden and enhance response rates. Because customers of the two companies received result disclosure reports that differed in both style and content from each other, the PGen Study survey questions were tailored to reflect the specific PGT experience of each company’s customers. For example, hypothetical scenarios used in the 2 W survey were populated with company-specific results reports corresponding to the company used by each participant, and BL and 6 M survey questions were designed to reflect categories of results provided by the company that each participant had utilized. Table 1 provides a summary of the items that appeared in each survey. Variables measured in the PGen Study encompassed four broad domains: personal characteristics (for example, demographic information, genetic literacy); personal health (for example, family medical history, health behaviors); health care outcomes (for example, insurance status, utilization of medical screening services); and behavioral responses to PGT (for example, perceived utility of results, satisfaction with experience). Table 1 also highlights items included in the 6 Month Non-Responder (6 M-NR) survey, which was presented to 6 M survey non-responders and is described in greater detail below.

**Table 1:** **Variable measurement across three time points in the PGen Study**

	**Survey**		
**Variable domains**	**Baseline (BL)**	**2 weeks (2 W)**	**6 months (6 M)**
**Personal characteristics**			
Demographic information	+		+
Emotional states	+	+	+
Genetic literacy	+	+	+
Numeracy	+		
Motivations and expectations	+		
**Personal health**			
Personal and medical family history	+		
Health status and health behaviors	+		+^a^
Disease risk perceptions	+	+	+
Conditions of interest	+		
**Health care**			
Insurance status	+		+^a^
Use of medical screening services	+		+
Use of medical diagnostic services			+
**Responses to personal genomic testing**			
Sharing of genetic results	+		+^a^
Reactions to genetic results		+	+
Perceived utility of results		+	+
Use of genetic results			+
Information-seeking behaviors			+
Satisfaction with experience			+^a^

A plus sign indicates measurement of variables within that specific category. ^a^Variables in this category were also evaluated on the 6 Month Non-Responder (6 M-NR) survey.

Because PGT customers in our surveys received their reports over the internet, a web-based survey was selected for data collection. This approach leveraged the benefits of web-based data collection - cost-efficient delivery of complex survey instruments; tailored content; and maximization of participant confidentiality [44] - without facing the usual concerns associated with this survey administration method (that is, not reaching members of the target population who are not regular internet users) [45]. With the help of Survey Sciences Group, LLC (SSG, Ann Arbor, MI, USA), each survey was programmed according to established standards for web-based survey design, including screen layout, standard question formats, and other visual design elements (for example, color, graphics) [46,47]. Each survey was interactive, with navigation buttons allowing respondents to move forward and backward within the survey. Responses were saved each time a navigation button was selected, permitting capture of partially completed survey data and allowing respondents to complete the survey in multiple sessions. Electronic timestamp capture enabled the research team to record timing of survey initiation and completion, and cumulative time spent across multiple sessions.

Following initial programming, each survey was thoroughly tested through well-established testing strategies [48], including a web-based survey specifications document to describe the intended design of the web survey and to use as a tool in programmer testing; survey operations testing (that is, testing conducted by survey system specialists unfamiliar with the PGen Study questionnaires); and research team testing (that is, testing by those familiar with the questionnaires, but not the survey system). Usability assessments ensured that the survey layout was intuitive and minimized risks to data quality.

### Participant recruitment and data collection

23andMe launched its service in November 2007, offering both ancestry and health-related information directly to customers; the company continued with this model until 22 November 2013, at which time provision of health-related information to new customers was halted after receiving an FDA Warning Letter. Pathway Genomics entered the PGT market in 2008 with a similar DTC service delivery model, but in 2010 modified their model to require physician order. For the purposes of enrollment in the PGen Study only, Pathway permitted direct ordering of PGT by customers without external physician referral, as described below.

Participant recruitment was restricted to new customers of 23andMe and Pathway between March and July 2012. Between March and July 2012, 3,900 23andMe customers who had recently purchased PGT, and had completed the company’s own informed consent process for participation in research, were contacted directly by the company with an email that provided an overview of the PGen Study and an invitation to participate. The standard cost of 23andMe services at the time of study enrollment was USD $99, although promotional discounts were sometimes available. Pathway recruited new customers for enrollment in the PGen Study in two ways: a banner advertisement was placed on the Pathway website, and an email was sent to approximately 30,000 members of PatientsLikeMe, a health-based social networking site [49]. Both approaches invited enrollment in the PGen Study and offered Pathway’s health-related PGT services for a subsidized cost of $25. Individuals who responded to these invitations were not required to have their personal physician order PGT; rather, these customers ordered their own testing from the Pathway website, in a similar fashion to 23andMe customers. Immediately after purchasing PGT, Pathway customers were brought to a webpage inviting them to participate in the PGen Study.

The PGen Study invitations included a link that directed customers to the SSG-supported web-based survey system. After affirming an online consent, participants agreed to have their de-identified genetic risk information and PGen Study survey responses shared with study investigators. After consent, participants were routed to the BL survey. Reminder emails were sent 3 and 6 days after the initial invitation to those customers who had not yet responded.

Eligibility criteria for follow-up in the PGen Study included completing the BL survey prior to viewing one’s PGT results and viewing one’s health-related PGT results within a defined period. Each company updated SSG every 1 to 2 weeks post-enrollment with the status of participants’ results reports, and those who completed the BL survey *after* viewing their results were excluded from follow-up.

Invitations to the 2 W survey were sent approximately 2 weeks after participants had accessed their results. If at the start of the 2 W survey a participant indicated that they had not viewed their health-related results, they were not permitted to complete the follow-up surveys until they had done so. Invitations to the 6 M survey were emailed to eligible participants approximately 6 months after they accessed their PGT results and eligibility for the 6 M survey did not require completion of the 2 W survey. For both the 2 W and 6 M surveys, reminder emails were sent to eligible non-responders 3 and 6 days after the initial invitation, and reminder letters were mailed to non-responders who had provided a mailing address in the BL survey.

We also designed an abbreviated survey directed toward 6 M survey non-responders (6 M-NR) that contained a subset of questions from the full 6 M survey (Table 1). This 5 minute survey was administered after completion of the 6 M survey data collection protocol, and provided a way for non-responders to quickly answer the most critical questions from the 6 M survey, to identify reasons for non-response to the 6 M survey, and to provide an additional opportunity to complete the 6 M survey. Upon completion of the 6 M-NR survey, participants were invited to immediately continue on to the full 6 M survey.

Participants were compensated for their participation with Amazon.com electronic gift cards, receiving $10 for completing the BL survey, $20 for the 2 W survey, and $20 for either the 6 M or 6 M-NR survey (but not both).

### Participant confidentiality

**Figure 2:**
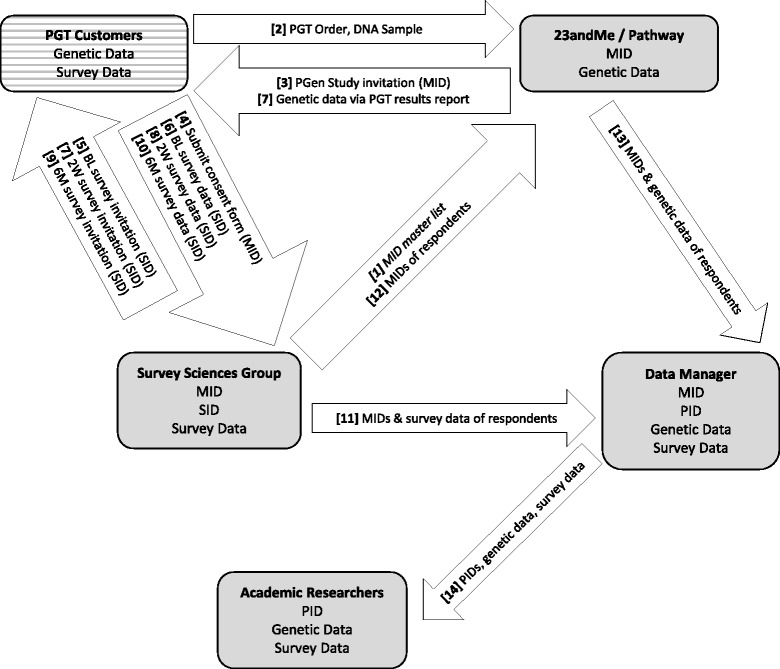
**Data transfer and protection in the PGen Study.** Heavily shaded boxes represent PGen Study team members; PGT customers/PGen Study participants are represented by a striped box. The contents of each box represent the data types available to each party. Numbers indicate the sequential steps involved in data transfer between PGen Study members (step 1 is italicized), and each arrow represents a one-way flow of data between parties. Data in parentheses were embedded in the survey invitations and were not visible to participants. PGT, personal genomic testing; MID, master identification number; SID, survey identification number; PID, personal identification number; BL, baseline; 2 W, 2 week; 6 M, 6 month.

Maintaining participant confidentiality as data flowed among researchers, PGT companies, and SSG was important to all concerned. The protocol for data flow was designed to ensure that no single institution would have access to all of three types of data: participant contact information, survey responses, and genetic risk reports (Figure 2). Prior to the start of the study, SSG created a set of master identifier numbers (MIDs) and provided each company with sufficient MIDs to accommodate all invitations to be sent to potential participants. The companies maintained a link between the MID and the customers that were invited, so that a link could later be made to their PGT results. When respondents entered the SSG survey system, the MID was passed in the web uniform resource locator (URL) and then saved to the data during the consent process. When respondents consented, the MID was then passed to the sample file that SSG maintained (which included all participant contact information) and a new Survey ID (SID) was created to individually track recruited participants through the survey process. The SID was stored in SSG’s master sample file along with the MID for later linking purposes. All respondent communications, survey invitations, and survey access points were then tracked and secured using the SID. The purpose of this separate SID was to ensure that once the final data were compiled, no individual (including respondents) would have an ID that could uniquely identify someone in the final study dataset.

After administration of all three surveys, SSG provided each company with a registry of MIDs for those customers who consented and completed the BL survey; each company was then able to link individual PGT reports to the MIDs of those who responded. Linked reports and MIDs for each customer (stripped of any contact information) were then sent to the PGen Study data manager. Meanwhile, survey data linked with the MID were sent directly from SSG to the PGen Study data manager. The data manager subsequently merged the survey data and risk reports using the MIDs.

Finally, the PGen Study data manager assigned each participant a new, randomly generated primary identification number (PID), ensuring that any party with access to MID-labeled data could not create a link to the final analytic data file. Any contact information provided by participants within the surveys was deleted, and the final PID-labeled dataset containing linked, but de-identified, survey data and risk reports was provided to the academic researchers.

Each PGT company was given a subset of the final dataset containing only their own customers. It was agreed that each company could freely use its own data; however, all parties agreed not to use PGen Study data for competitive marketing purposes. Although no explicit initial discussions were held about the use of company-specific data for regulatory purposes, this possibility was discussed following receipt of the FDA Warning Letter by 23andMe, and it was agreed that the companies could do so.

### Data analysis

Baseline demographic characteristics were tabulated for each sample of survey respondents. Chi-square tests and *t*-tests were used to compare each follow-up survey sample (2 W, 6 M, 6 M-NR) to BL survey responders to evaluate the presence of differential attrition by follow-up time. An analysis of substantive non-response bias was also performed by randomly selecting six BL questions (addressing behavioral, psychological, motivational, and health factors) and then using *t*-tests and chi-squared tests to compare responses from participants who submitted both BL and 6 M surveys to responses from participants who submitted a BL, but not a 6 M survey.

## Results

The BL, 2 W, and 6 M surveys comprised 240, 77, and 248 questions, respectively; however, all participants saw only a subset of these questions, tailored to their PGT experience and reflecting earlier responses. Median response times among those who completed each survey were 27 minutes for the BL survey, 22 minutes for the 2 W survey, and 32 minutes for the 6 M survey. Median time to survey initiation after viewing one’s PGT results was 17 days (2.4 weeks) for the 2 W survey and 190 days (6.3 months) for the 6 M survey. Complete versions of each survey are available online (Additional files 1, 2, 3 and 4).

**Figure 3:**
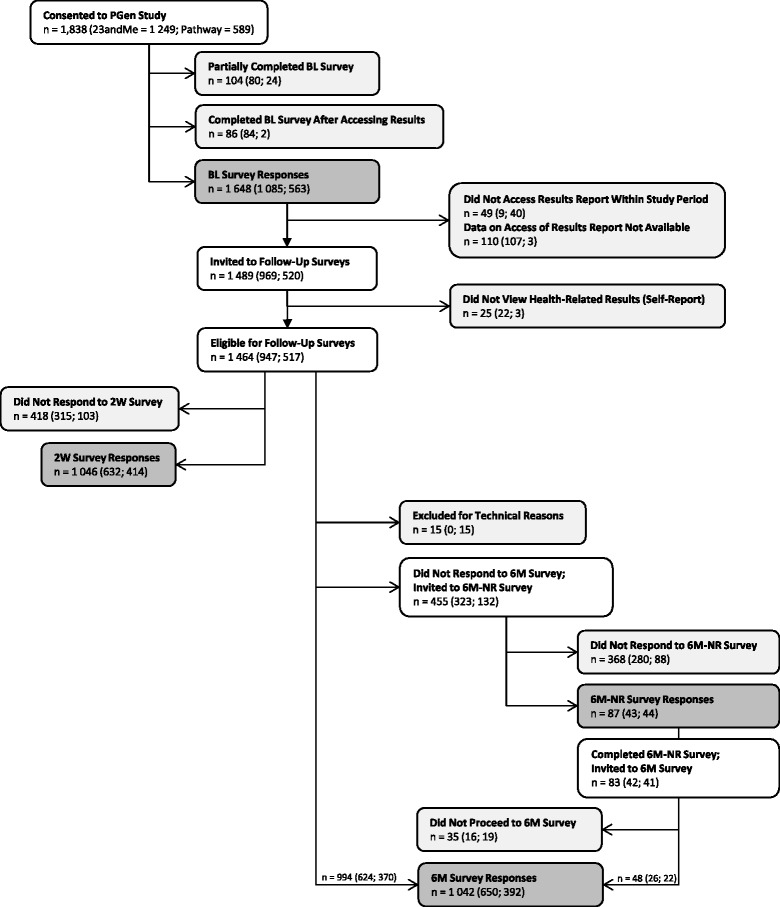
**PGen Study data collection strategy and enrollment results.** Heavily shaded boxes represent data collection points; lightly shaded boxes represent loss-to-follow-up or exclusion criteria.

Of 1,838 participants who began the BL survey, 1,648 completed the survey before accessing their PGT results (Figure 3). Of these, 1,464 met eligibility criteria for follow-up, including viewing of their PGT results within the study period. Response rates were 71.4% at 2 W and 71.1% at 6 M. Nearly all of these responses were fully completed surveys, resulting in a completion rate [50] of 98.1% at 2 W and 95.7% at 6 M. Of those eligible for follow-up, 933 (63.7%) submitted surveys at all three time points, and 1,155 (78.9%) submitted at least one follow-up survey. Among 6 M survey responders, 91.3% agreed to be re-contacted about opportunities to participate in future research, such as follow-up surveys or interviews. The 6 M-NR increased response rates for the 6 M survey: of the 455 non-responders to the 6 M survey, 87 responded to the 6 M-NR survey invitation; of these, 48 went on to submit a full 6 M survey.

A limited amount of demographic information was available from the 3,900 23andMe customers invited to the PGen Study to enable a comparison of invitees and participants. The 947 eligible BL survey responders from 23andMe were more likely to be female (56.9% versus 47.8%; X^2^ = 34.4, *P* <0.0001), but were similar to the 3,900 invitees with respect to Latino ethnicity (5.6% of responders versus 5.2% of invitees; X^2^ = 0.3, *P* =0.58), and age (50.9 ± 16.1 years versus 50.1 ± 15.8 years; t =1.4, *P* =0.16). A comparison of invited and enrolled Pathway customers would have required demographic data from the PatientsLikeMe members who were invited to participate and these data were not available to the PGen Study team.

Table 2 summarizes the demographic characteristics of PGen Study respondents for each survey. *t*-Tests and chi-squared tests demonstrated no significant differences (*P* >0.05) in demographic characteristics between eligible BL survey responders and either 2 W responders or 6 M responders. 6 M-NR survey responders differed from eligible BL survey responders only with respect to PGT company in that 6 M-NR survey responders were significantly less likely than BL survey responders to have had their testing through 23andMe (49.4% 23andMe customers at 6 M-NR versus 64.7% at BL; X^2^ = 8.8, *P* =0.003). The final 6 M responder sample, however, including those recruited to the 6 M survey following completion of the 6 M-NR survey, did not significantly differ from eligible BL responders with respect to PGT company (62.4% 23andMe customers at 6 M versus 64.7% at BL; X^2^ = 1.4, *P* =0.24). Relative to the 2013 United States population [51], PGen Study BL survey responders were older (median age 47.0 years versus 37.5 years), more likely to be female (61.2% versus 50.8%), less likely to be non-white (15.7% versus 22.3%) or Hispanic/Latino (5.5% versus 17.1%), more highly educated (78.2% college graduates versus 28.5%), slightly more likely to be married (54.4% versus 48.0%), and more likely to have health insurance coverage (94.7% versus 85.5%).

**Table 2:** **Demographic characteristics of PGen Study participants**

	**BL survey responders eligible for follow-up**	**2 W survey responders**	**6 M survey responders**	**6 M-NR survey responders**
**n**	1,464	1,046	1,042	86
**Mean age (±SD; range)**	47.5 (15.5; 19-94)	46.7 (15.7; 19-91)	46.9 (15.6; 19-94)	47.8 (15.5; 23-94)
**Mean self-reported health (±SD)** ^**a**^	2.49 (1.01)	2.50 (1.0)	2.49 (1.0)	2.60 (1.1)
**Female (%)**	61.2	60.1	60.2	70.9
**Annual income (%)**				
<$40,000	16.8	17.5	17.1	18.4
$40,000-$99,999	38.8	38.0	38.8	40.2
$100,000-$199,999	31.7	32.2	31.3	31.0
≥$200,000	12.7	12.3	12.8	10.4
**Education (%)**				
<College degree	21.8	21.0	20.4	25.3
College degree	30.6	30.2	30.0	34.5
Some graduate school	35.0	35.6	36.4	28.7
Doctoral degree	12.6	13.2	13.2	11.5
**Marital status (%)**				
Single	19.2	19.6	19.9	13.8
Long-term partner	13.7	15.2	14.7	13.8
Married	54.4	52.5	52.5	57.5
Widowed/divorced/separated	12.7	12.7	12.9	14.9
**Health insurance (%)**	94.7	95.5	95.3	96.6
**United States residency (%)**	97.9	98.0	98.2	97.7
**Non-white (%)**	15.7	14.7	14.2	10.3
**Hispanic/Latino (%)**	5.5	4.6	5.1	3.5
**23andMe customers (%)**	64.7	60.4	62.4	49.4

^a^Self-reported health was measured on a 5-point scale from 1 (poor) to 5 (excellent).

SD, standard deviation.

Six BL survey questions were randomly selected for the non-response bias analysis. These questions evaluated, respectively, motivations for undergoing PGT; beliefs about PGT utility; perceived autonomy in health care decision making; recent history of anxiety; perceived risk of Alzheimer’s disease; and body mass index (BMI). Chi-squared tests and *t*-tests to compare responses to each question demonstrated no significant differences between the 1,042 participants who completed both BL and 6 M surveys and the 422 participants who completed the BL but not 6 M survey (all *P* >0.05).

## Discussion

Empirical data on the PGT customer experience is important to a range of stakeholders, but is difficult to obtain without explicit cooperation between researchers and industry partners. Here we describe the design and implementation of the PGen Study, a longitudinal study of PGT customers performed in cooperation with two PGT companies, and present the surveys themselves along with initial data on the cohort. Careful and transparent navigation of the industry-academic partnership was important throughout the design and data collection stages of the PGen Study, and will continue to be important as we further analyze and report these data.

Response rates surpassed 70% at both short-term and long-term follow-up, and were achieved by optimizing the participant experience through a user-friendly survey design. Although minimization of participant burden is always a consideration in study design, here it was particularly important to company representatives that their customers not have a negative research experience. In surveying PGT customers who had ordered testing online, the ability to direct respondents to a web-based survey via email invitation was also key to obtaining high response rates. The value of following up non-respondents with an alternative invitation strategy was also demonstrated, as use of the 6 M-NR survey reduced overall attrition rates, and did so without evidence of differential attrition, a major source of concern in online longitudinal measurement [52,53]. Ultimately, we achieved a set of surveys that collectively administered nearly 600 questions while maintaining high response, completion, and retention rates.

To ensure that data collection did not interfere with the user experience of PGT, the PGen Study integrated data collection into the commercial PGT process. Communication and cooperation between the study collaborators was essential, and enabled us to dynamically assess participant eligibility throughout the data collection phase and ensure accurate timing of data collection based on date of results delivery.

Through these features, the PGen Study has potentially achieved a more naturalistic study of the PGT experience than previous longitudinal research on PGT users. For example, the Scripps Genomic Health Initiative (SGHI) also collaborated with an existing PGT company [39], but subsidized testing was provided on the condition of consent to the SGHI, participants could ask study investigators questions about PGT at the time of sample collection, and participants were recruited from among employees of a medical research institute focused on personalized healthcare.

In the PGen Study, the task of linking a participant’s survey responses with their individual genetic results was accomplished by leveraging the expertise of SSG in large-scale data management, and by creating a tightly controlled data transfer process between study partners. Access to participants’ actual PGT results has been available in several previous longitudinal studies of PGT, including the SGHI study, the Multiplex Initiative (Multiplex) [24], and the Coriell Personalized Medicine Collaboration [54]. The Multiplex model, however, included PGT for only eight conditions, and was provided to members of a large integrated health system, who received educational counseling with their results. The Coriell model integrated PGT into clinical care through patient education, optional genetic counseling, and physician review of results.

The PGen Study data transfer process maintained participant confidentiality by ensuring that neither company had access to data on the other company’s customers, and that no company had access to identifiable survey data for its own customers. This is the first study of PGT to rely on a three-party data transfer model, and is the only academic study in the field of clinical genetics to engage a commercial genetic testing company and third-party research firm in such a way.

A single, comprehensive, yet fully de-identified PGen Study dataset with longitudinal data on over 1,000 actual PGT customers is now available. A sample of PGT customers is not expected to be a random sample of the US population, and in fact, PGen Study participants differed from the general population with respect to age and sex distribution, ethnicity, education level, and health insurance coverage. At the same time, PGen Study participants represented a wide distribution of income and education levels, marital status, and ages, with a sizeable proportion (approximately 15%) being of non-white ethnicity. These features of the PGen Study cohort will facilitate future subgroup analyses.

The PGen Study team faced the challenge of engaging two PGT companies in a way that maintained the uncensored pursuit of research questions, yet ensured that neither company would be promoted or slighted, and allowed all parties the opportunity to continue to have a voice in ongoing research and analysis. All parties agreed that none of the researchers would engage in comparisons of the two PGT companies, particularly with respect to endpoints that could be relevant to commercial marketing (for example, customer satisfaction). This agreement has led to considerable discussion within the study team about when comparison of participants between companies may be appropriate on statistical grounds, and we expect to revisit this question with each new analysis of PGen Study data.

These subjects have required an ongoing and transparent dialogue between the principal investigators and PGT company scientists. However, careful planning of the PGen Study has thus far been conducted through a collegial relationship between study members and a shared vision for the study, with discussions and negotiations to date being respectful and fruitful. All parties have acknowledged the potential for conflicts of interest from the outset, and have worked together to maintain rigorous data collection and analysis standards.

Benefits of the PGen Study as a data source include its large and diverse sample; wide-ranging source population; longitudinal design; high response rates; expansive set of survey questions; inclusion of participants’ PGT results; and the high proportion of participants agreeing to future follow-up. Limitations include those inherent to voluntary study enrollment, such as the potential for selection bias in restricting the sample to voluntary enrollees and in excluding from longitudinal analyses those who did not complete follow-up surveys.

In addition, our findings are potentially generalizable only to other PGT customers utilizing a DTC model similar to those of 23andMe and Pathway at the time of the study, and not to individuals who undergo physician-mediated PGT, who have not voluntarily sought PGT, or who have obtained clinical genetic testing. Our decision to focus on the DTC PGT was, however, intentional: because the DTC model is the most extreme version of PGT (with respect to its dissimilarity to clinical genetic testing), it is the most likely to reveal evidence of harm from PGT, and would provide the greatest support for PGT if evidence of consumer benefit is observed.

A related but separate limitation is the fact that Pathway participants were recruited through the offer of low-cost subsidized testing, whereas 23andMe participants were not. Pathway participants in our study may differ in meaningful ways from both typical Pathway participants and 23andMe participants who paid more for their testing. Future analyses of PGen Study data will investigate testing cost and its impact on motivations and test satisfaction, and results will be stratified by testing company where appropriate.

Our data collection was completed prior to the FDA’s Warning Letter to 23andMe. However, we recognize that the FDA Warning Letter represents a significant shift in the regulation of PGT, and one that will surely impact public opinion and utilization of these services if and when they are reinstated. Moreover, the field of PGT, including its technology and service models, evolves quickly, and major changes to the industry could lessen the relevance of PGen Study data over time.

## Conclusions

We have reported on the design, implementation, and participant characteristics of a new prospective cohort study of PGT customers, highlighted state-of-the-art online survey methods capable of both minimizing costs and maximizing data integrity, and detailed a data security protocol that ensures participant confidentiality while permitting ease of data transfer between academic and industry partners. For those developing studies of new health technologies, particularly ones marketed directly to private customers, the PGen Study may serve as a model of successful industry engagement that maintains academic independence. For stakeholders in the debate surrounding PGT, the PGen Study provides a large and comprehensive data source, with high completion and response rates, that is ripe for further interrogation.

## Additional files

Additional file 1:
**Baseline PGen Study survey.**
Additional file 2:
**2 week follow-up PGen Study survey.**
Additional file 3:
**6 month follow-up PGen Study survey.**
Additional file 4:
**6 month follow-up non-responder PGen Study survey.**

## Abbreviations

2 W: 2 weeks post-results
6 M: 6 months post-results
6 M-NR: 6 Month Non-Responder
BL: baseline
DTC: direct-to-consumer
FDA: United States Food and Drug Administration
MID: master identifier number
PGen: Impact of Personal Genomics
PGT: personal genomic testing
PID: primary identification number
SGHI: Scripps Genomic Health Initiative
SID: Survey ID
SSG: Survey Sciences Group
