# Supplementary material for: Design, methods, and participant characteristics of the Impact of Personal Genomics (PGen) Study, a prospective cohort study of direct-to-consumer personal genomic testing customers
Source: Genome Med. 2014 Dec 3;6(12):96. doi: 10.1186/s13073-014-0096-0 (PMC4256737; doi:10.1186/s13073-014-0096-0)
Supplement: Additional file 1: — Baseline PGen Study survey. [file 13073_2014_96_MOESM1_ESM.pdf]

Baseline Questionnaire Specifications for PGen/ S10028 (F9)

Short URL to direct towards production survey	https:www.ssgresearch.com/pgen
Support email address to include in header	pgen@ssgresearch.com
Support phone number to include in header (if needed)	

Logo to use if other than SSG logo
Please list network location of other logo to use:

Mandatoriness (check the appropriate setting)

	All questions are optional unless otherwise noted
x	All questions are optional with a soft prompt included if no answer is provided
	All questions are mandatory

Please provide text to use for Mandatoriness prompt if being used (Default text to use is provided below):

{See survey for custom prompt text on DEM1-DEM4}

General:

We noticed that you did not answer a question on the previous page. It is important to us that we get a complete set of responses from you. Please return to the previous page by clicking "Previous" and select an answer for each question. If you would rather not select an answer, you may instead continue to the next page by clicking "Next."

Other specify:

You selected 'Other' but did not specify your answer. Please return to the last question by clicking “Previous” and type in your specific answer. If you would rather not specify an answer, you may instead continue to the next page by clicking “Next.”

Header Sections (if being used)

Section Label	Questions in Section	Section Label	Questions in Section
DEM1-DEM4	Demographics	Risk Perceptions	E1
Q1- CR2_6a	Your Use of Personal Genomic Services	Health, Behaviors and Insurance	F1-J4
C1- C5m_10_5	Conditions of Interest	Genetics and Numbers	L1-M5
D1	How You’re Feeling	About You	N1-K11

Survey Title to appear in header (appears above the section header bar)

The Impact of Personal Genomics (PGen) Study

Welcome Page text (please modify the following as needed)

{NOTE: WE WILL AUTO-AUTHENTICATE RESPONDENTS INTO THIS SURVEY, SO THE LOGIN SCREEN WILL NEVER BE SHOWN UNLESS THE RESPONDENT TIMES OUT}

Welcome to the Impact of Personal Genomics (PGen) Study!
This is the first of three surveys for this study.

Please enter your User ID, then click Start Survey to begin! If you do not know what your ID is, please email pgen@ssgresearch.com.

User ID _______________

Resume Page text (please modify the following as needed)

Thank you for returning to the survey. Please click "RESUME SURVEY" to begin where you last left off…

End Page Text

Thank you for your participation! This concludes the first study survey.

Within the next 2 weeks, you will receive a $10 Amazon.com gift certificate via email.

We will email you with a link to the second study survey shortly after you receive your personal genomic results.

You may now close your browser.

Survey Title appearing in browser window

PGen Study

GENERAL PROGRAMMING NOTES

Program DEM1-DEM4 in separate survey and only pass respondents into the main survey if all items in this initial section have been answered.

We will need to pass both DATSTAT_ALTPID and DEM3 into the second survey.

Soft prompts will be programmed on each question as well as "specify" text responses.

All emphasis should be programmed in black, all caps text instead of lowercase blue text.

Preloads

PRE_1 Genetics Company
- 1 Pathway Genomics
- 2 23andMe

Calculations

{Note: Will receive email addresses below at later date; program using placeholders for now}

CALC_COMPANY_EMAIL. {{Value:PRE_1}==1 ? "{Pathway email address}" : "{23andMe email address}"}

Consent

{NOTE: Center first two lines on screen; NO scrolling text box for consent; add PDF link at top of screen (separate PDFs for each company)}

Welcome to the Impact of Personal Genomics (PGen) Study!

Please read this consent form, and then make one of the choices below.

{Note: See P:\Surveys\S10028 PGen Study\Questionnaire\Consent - Approved by IRB\PGen_Consent_generic_version 4_1.9.12_FOR WEB SURVEY}

{NOTE: Add space between consent options}
{PRG: TIMESTAMP UPON QCONSENT SUBMISSION}

QCONSENT.

1 I have read this consent form, and I agree to the study procedures described above. I attest that I am 18 years or older. I attest that I have signed up for personal genomic testing through {DISPLAY PRE_1}, and I have received an email requesting my participation in this research study. I understand that some of my health-related genetic results will be de-identified and shared with the study researchers. I understand that my genetic results will NOT be linked to my email address or any other identifying information in the context of this research study.

2 I do NOT wish to participate in this study.

{Note: Section Header: "Demographics"}

{PRG: DEM SECTION IN SEPARATE SURVEY FROM MAIN INSTRUMENT; RESPONDENTS WILL PASS INTO MAIN SURVEY UPON SUBMISSION}
{PRG: PASS DEM3 INTO SECOND SURVEY}

{PRG: SAME SCREEN DEM1-DEM4}

{PRG: MAKE DEM1-DEM4 REQUIRED}
{PRG: DISPLAY CUSTOM ERROR MESSAGE IF ANY OF DEM1-DEM4 ARE NOT ANSWERED}

We noticed that you did not answer one or more of the required questions on this page. Please provide the missing information. Or, if you have decided not to participate in this study, please close your browser to exit the survey.

DEM1-DEM4_TEXT. The first four questions of this survey ask for your email address, year of birth, sex, and country of residence. These four items are required in order to participate in this study.

{PRG: VALIDATE EMAIL ADDRESSES}
DEM1-1a. What is your email address?
(Please enter in the format email@address.com)

DEM1. Email address: [EMAIL ADDRESS]
DEM1a. Confirm email address: [EMAIL ADDRESS]

DEM2. What year were you born?

[NUMERIC 1875-1994]

DEM3. What is your sex?

1 Male
2 Female

{PRG: DEM4.TEXT IS OPTIONAL}
DEM4. Where do you currently reside?

1 In the United States
2 Outside of the U.S. *(Please specify country)* [TEXT RESPONSE]

DEM6. First name (optional): [TEXT RESPONSE]

{PRG: NO SOFT PROMPT ON DEM5 SCREEN}

DEM5. If you would like to receive a notification letter in the mail before the second and third study surveys, please provide us with your mailing address (optional):

DEM5a. Street 1: [TEXT RESPONSE]
DEM5b. Street 2: [TEXT RESPONSE]
DEM5c. City: [TEXT RESPONSE]
DEM5d. State/Province: [TEXT RESPONSE]

DEM5e. Country: [TEXT RESPONSE]
DEM5f. Zip/Postal Code: [TEXT RESPONSE, LIMIT TO 10 CHARACTERS]

DEM5g. Other/International Address Information:
[OPEN END RESPONSE]

{Note: Section Header: “Your Use of Personal Genomic Services”}

SECT1_TEXT. Section 1 of 7: Your Use of Personal Genomic Services

Q1. Think about the process you went through in deciding to seek {DISPLAY PRE_1}'s service. Over what period of time did you make this decision?

1 A day or less
2 Several days
3 One to two weeks
4 Several weeks
5 Several months or more
6 The {DISPLAY PRE_1} service was a gift and therefore I did not make the decision to seek this service

---

Q2. Approximately how much time did you spend reading information at the [DISPLAY PRE_1] website before deciding to be tested?

1 I did not spend any time reading information at the [DISPLAY PRE_1] website
2 Less than 15 minutes
3 15 minutes to 1 hour
4 1 to 2 hours
5 Greater than 2 hours

Q3. Did you talk with anyone to help you make your decision to seek {DISPLAY PRE_1}'s service?

1 Yes
0 No

{PRG: SHOW Q3a IF Q3=1; OTHERWISE SKIP TO Q4}
{PRG: SELECT ALL THAT APPLY}

Q3a. Who helped you make your decision to seek {DISPLAY PRE_1}'s service?
(Select all that apply)

1 Family member
2 Friends
3 Co-workers/colleagues
4 Health care provider (e.g. your physician, physician assistant, nurse)
5 Genetics health care provider (e.g. a clinical geneticist or genetic counselor)
6 Representative from [DISPLAY PRE_1]
7 Other *(Please specify)*: [TEXT RESPONSE]

Q4. Have you ever purchased personal genomic services from a different company?

1 Yes
0 No

{PRG: SHOW Q4a IF Q4=1; OTHERWISE SKIP TO Q5}

Q4a. From which other company did you purchase personal genomic services?

[OPEN END RESPONSE]

{PRG: Q5 SELECT ALL THAT APPLY}

Q5. Now, thinking about your decision to seek {DISPLAY PRE_1}'s service, how did you first hear about {DISPLAY PRE_1}?
(Please select all that apply)

1 A community or health related organization *(Please specify:)* [TEXT RESPONSE]
2 Books
3 Brochures, pamphlets, etc
4 Doctor or health care provider
5 Family member
6 Friend/co-worker
7 Internet
8 Newspapers/Magazines
9 Radio/TV program
10 Talk/lecture/presentation
11 Other *(Please specify):* [TEXT RESPONSE]

{IF Q1 ≠ 6, SHOW Q6; OTHERWISE SKIP TO FILTER BEFORE Q7}
{PRG. NOTE: ENLARGE TEXT BOX (60 x 3)}

Q6.
We are interested in learning about reasons why people seek out personal genomic services such as {DISPLAY PRE_1}'s service. Please tell us about why you sought this service in the space provided below.

[OPEN END RESPONSE]

{PRG: SHOW Q7 IF Q1 = 6, OTHERWISE SKIP TO B1}

Q7. If the {DISPLAY PRE_1} service was a gift to you, please tell us about why you decided to send in a sample.

[OPEN END RESPONSE]

{DESIGN: GRID B1_1- B1_8}

B1. People seek personal genomic testing for a number of different reasons. For each of the following statements, please select the response that is most appropriate for you.

How important were the following factors in your decision to seek personal genomic testing?

1 Not at all Important
2 Somewhat Important
3 Very Important

B1_1. Curiosity about my genetic makeup
B1_2. Interest in finding out about my personal risk for specific diseases
B1_3. Desire to learn about my genetic makeup without going through a physician
B1_4. Desire to improve my health
B1_5. Interest in finding out about my individual response to different types of medications
B1_6. Desire to create a better plan for the future
B1_7. Personal interest in genetics in general
B1_8. The service seemed like it would be fun and entertaining

---

{DESIGN: GRID B1_9- B1_12}

B1. People seek personal genomic testing for a number of different reasons. For each of the following statements, please select the response that is most appropriate for you.

How important were the following factors in your decision to seek personal genomic testing?

1 Not at all Important
2 Somewhat Important
3 Very Important
4 N/A - Not Applicable

B1_9. Other members of my family are using personal genomic services
B1_10. Desire to learn more about my genetics because I have limited information about my family health history
B1_11. Desire to learn more about my genetics because I am adopted
B1_12. Interest in getting information about the risk of health conditions for my current children or future children

---

{DESIGN: GRID B2_1-B2_7}
{DESIGN: SAME SCREEN B2-B2_OTH}
{PRG: NO SOFT PROMPT ON B2_OTH}

B2. How much did you consider the following factors when deciding whether or not to seek personal genomic testing?

1 Did not consider
2 Considered somewhat
3 Considered a lot

B2_1. How well the results predict whether or not I'm going to get a particular disease
B2_2. Privacy of my genetic information
B2_3. Whether or not there are health-related actions I can take as a result of learning my genetic information
B2_4. The possibility that I might receive unwanted information
B2_5. Cost of services
B2_6. The education materials made available through the company
B2_7. The convenience of being tested at home

B2_OTH. If you considered any other factors when deciding whether or not to seek personal genomic testing, please list them here.

[OPEN END RESPONSE]

B3. Please indicate how much you agree or disagree with the following statements.

1 Strongly disagree
2 Somewhat disagree
3 Neither agree nor disagree
4 Somewhat agree
5 Strongly agree

B3a. I trust {DISPLAY PRE_1} to use my genetic information only for the purposes to which I agreed when I signed up for {DISPLAY PRE_1}'s service.
B3b. I trust {DISPLAY PRE_1} to keep my genetic information and medical information confidential or private.

B4. Please indicate how much you agree or disagree with the following statement: What I learn from my personal genomic testing can help reduce my chances of getting sick.

1 Strongly disagree
2 Somewhat disagree
3 Neither agree nor disagree
4 Somewhat agree
5 Strongly agree

CR1. Do you plan to discuss what you find out from {DISPLAY PRE_1} with anyone?

1 Yes
0 No

{PRG: SHOW CR2 IF CR1 = 1, OTHERWISE SKIP TO C1}

CR2. With whom do you plan to discuss your {DISPLAY PRE_1} results?
(Select all that apply)

1 Family members
2 Friends
3 Co-workers/colleagues
4 Primary care provider
5 Genetics specialist (e.g. genetic counselor, clinical geneticist)
6 Other medical professional
7 Contacts on social networking services (e.g. Facebook, MySpace, Twitter)
8 Contacts on health- or disease-based social networking services (e.g. Patients Like Me, 23andMe's discussion forum, Cure Together, disease-specific patient networks)
9 Other *(Please specify)* [TEXT RESPONSE]

{PRG: SHOW CR2_1a IF CR2=1, OTHERWISE SKIP TO FILTER BEFORE CR2_6a}

CR2_1a. With which family members do you plan to talk about your {DISPLAY PRE_1} results?
(Select all that apply)

1 Spouse/significant other
2 Children
3 Brothers or sisters
4 Parents
5 Other relatives *(Please specify)* [TEXT RESPONSE]

{PRG: SHOW CR2_6a IF CR2=6, OTHERWISE SKIP TO C1}
{PRG: CR2_6a SELECT ALL THAT APPLY}

CR2_6a. With which other medical professional(s) do you plan to talk about your {DISPLAY PRE_1} results? *(Select all that apply)*

1 Anesthesiologist
2 Nutritionist
3 Obstetrician/Gynecologist
4 Oncologist
5 Physician assistant, nurse, or medical assistant
6 Reproductive Endocrinologist
7 Surgeon
8 Pediatrician/Child's physician
9 Other specialist/Other medical professional *(Please specify)* [TEXT RESPONSE]

{Note: Section Header: "Conditions of Interest"}

{DESIGN: GRID C1_1-C1_4}

SECT2_TEXT. Section 2 of 7: Conditions of Interest

C1. To what extent are you interested in learning about the following types of information?

1 Not at all interested
2 Somewhat interested
3 Very interested

C1_1. Risk of disease or health condition
C1_2. Drug response (medication)
C1_3. Carrier status (e.g. for pre-pregnancy planning)
C1_5. Ancestry
{PRG: SHOW C1_6 IF PRE_1=2}
C1_6. Traits

{DESIGN GRID: C2_1-C2_34}
{PRG: NO SOFT PROMPT ON C2_OTH}

C2. How interested are you in learning about your genetic risk for each of these diseases?

1 Not at all Interested
2 Somewhat Interested
3 Very Interested

C2_1 Osteoarthritis
C2_2 Rheumatoid arthritis
C2_3 Asthma
C2_6 Celiac disease
C2_8 Ulcerative colitis
C2_9 Breast cancer *(females only)*
C2_10 Colorectal cancer
C2_12 Leukemia
C2_13 Lung cancer
C2_14 Prostate cancer *(males only)*
C2_15 Skin cancer (Melanoma)
C2_17 Heart disease (Coronary artery disease)
C2_20 Blood clotting (Venous thromboembolism)
C2_21 Chronic kidney disease
C2_22 High cholesterol
C2_23 Diabetes
C2_24 Age-related macular degeneration
C2_25 Glaucoma
C2_26 Bipolar disorder
C2_27 Alzheimer's disease
C2_28 ALS (Lou Gehrig's disease)
C2_29 Multiple sclerosis
C2_30 Parkinson's disease
C2_31 Obesity

C2_OTH. If you are somewhat or very interested in learning about your genetic risk for any **other** diseases, please list them here.

[OPEN END RESPONSE]

C3. Has a doctor every told you that you have (or had) any of the following medical conditions?

1 Yes
0 No

C3_1. Arthritis
C3_2. Asthma
C3_3. Cancer
C3_4. Chronic kidney disease
C3_5. Diabetes
C3_6. Eye conditions
C3_7. Gastrointestinal (GI) conditions
C3_8. Heart conditions
C3_9. High cholesterol
C3_10. Lupus
C3_11. Mental illness/psychiatric conditions
C3_12. Neurological conditions (e.g. Alzheimer's disease, ALS, Multiple sclerosis, Parkinson's disease)
C3_13. Obesity
C3_14. Psoriasis

{PRG: SHOW C3_1_1-13 IF ANY C3_1-C3_13=1, OTHERWISE SKIP TO FILTER BEFORE C3_1a}
C3_1_1-13. Do you **currently** have any of the following medical conditions?

1 Yes
0 No

{PRG: SHOW C3_1_1 IF C3_1=1}
C3_1_1. Arthritis
{PRG: SHOW C3_1_2 IF C3_2=1}
C3_1_2. Asthma
{PRG: SHOW C3_1_3 IF C3_3=1}
C3_1_3. Cancer
{PRG: SHOW C3_1_4 IF C3_4=1}
C3_1_4. Chronic kidney disease
{PRG: SHOW C3_1_5 IF C3_5=1}
C3_1_5. Diabetes
{PRG: SHOW C3_1_6 IF C3_6=1}
C3_1_6. Eye conditions
{PRG: SHOW C3_1_7 IF C3_7=1}
C3_1_7. Gastrointestinal (GI) conditions
{PRG: SHOW C3_1_8 IF C3_8=1}
C3_1_8. Heart conditions
{PRG: SHOW C3_1_9 IF C3_9=1}

C3_1_9. High cholesterol
{PRG: SHOW C3_1_10 IF C3_10=1}
C3_1_10. Lupus
{PRG: SHOW C3_1_11 IF C3_11=1}
C3_1_11. Mental illness/psychiatric conditions
{PRG: SHOW C3_1_12 IF C3_12=1}
C3_1_12. Neurological conditions (e.g. Alzheimer's disease, ALS, Multiple sclerosis, Parkinson's disease)
{PRG: SHOW C3_1_13 IF C3_13=1}
C3_1_13. Obesity
{PRG: SHOW C3_1_14 IF C3_14=1}
C3_1_14. Psoriasis

{PRG: SHOW C3_1a IF C3_1=1; OTHERWISE SKIP TO FILTER BEFORE C3_3a}

C3_1a. Which of the following types of **arthritis** has a doctor told you that you have/had?
(*Please select all that apply.)*

1 Osteoarthritis ("wear and tear" on joints)
2 Rheumatoid arthritis (joint swelling and stiffness)
3 Other *(Please specify)* [TEXT RESPONSE]

{PRG: SHOW C3_3a IF C3_3=1; OTHERWISE SKIP TO FILTER BEFORE C3_4a}

C3_3a. Which of the following types of **cancer** has a doctor told you that you have/had?
(*Please select all that apply.)*

{PRG: SHOW C2_3a.6 IF DEM3=2}
1 Breast cancer
2 Colorectal cancer
3 Esophageal cancer
4 Leukemia
5 Lung cancer
{PRG: SHOW C2_3a.6 IF DEM3=1}
6 Prostate cancer
7 Skin cancer (Melanoma)
8 Stomach cancer
9 Other *(Please specify)* [TEXT RESPONSE]

{PRG: SHOW C3_5a IF C3_5=1; OTHERWISE SKIP TO FILTER BEFORE C3_6a}

C3_5a. Which of the following types of **diabetes** has a doctor told you that you have/had?
(*Please select all that apply.)*

1 Type 1 (insulin dependent, juvenile onset)
2 Type 2 (non-insulin dependent)

{PRG: SHOW C3_6a IF C3_6=1; OTHERWISE SKIP TO FILTER BEFORE C3_7a}

C3_6a. Which of the following **eye conditions** has a doctor told you that you have/had?
(*Please select all that apply.)*

1 Age-related macular degeneration
2 Glaucoma
3 Cataracts
4 Other *(Please specify)* [TEXT RESPONSE]

{PRG: SHOW C3_7a IF C3_7=1; OTHERWISE SKIP TO FILTER BEFORE C3_8a}

C3_7a. Which of the following **gastrointestinal (GI) conditions** has a doctor told you that you have/had?
(*Please select all that apply.)*

1 Celiac disease
2 Crohn's disease
3 Ulcerative colitis
4 Other *(Please specify)* [TEXT RESPONSE]

{PRG: SHOW C3_8a IF C3_8=1; OTHERWISE SKIP TO FILTER BEFORE C3_7a}

C3_8a. Which of the following **heart conditions** has a doctor told you that you have/had? (*Please select all that apply.)*

1 Irregular heartbeat (Atrial fibrillation)
2 Coronary artery disease
3 Peripheral arterial disease
4 Blood clotting (Venous thromboembolism)
5 Other *(Please specify)* [TEXT RESPONSE]

{PRG: SHOW C3_11a IF C3_11=1; OTHERWISE SKIP TO FILTER BEFORE C3_12a}

C3_11a. Which of the following **mental illness/psychiatric conditions** has a doctor told you that you have/had? (Please select all that apply.)

1 Bipolar disorder
2 Depression
3 Anxiety disorder
4 Other (Please specify) [TEXT RESPONSE]

{PRG: SHOW C3_12a IF C3_12=1; OTHERWISE SKIP TO C4}

C3_12a. Which of the following **neurological conditions** has a doctor told you that you have/had?
(*Please select all that apply.)*

1 Alzheimer's disease
2 ALS (Lou Gehrig's disease)
3 Multiple sclerosis
4 Parkinson's disease

5 Other *(Please specify)* [TEXT RESPONSE]

{DESIGN: GRID C4_1-C4_13}

C4. Have any of your blood relatives (a parent~~s~~, brother or sister, child~~ren~~, grandparent~~s~~, aunt~~s~~, uncle~~s~~, or first cousin~~s~~) ever had any of the following conditions?

1 Yes
0 No

C4_1. Arthritis
C4_2. Asthma
C4_3. Cancer
C4_4. Chronic kidney disease
C4_5. Diabetes
C4_6. Eye conditions
C4_7. Gastrointestinal (GI) conditions
C4_8. Heart conditions
C4_9. High cholesterol
C4_10. Lupus
C4_11. Mental illness/psychiatric conditions
C4_12. Neurological conditions (e.g. Alzheimer's disease, ALS, Multiple sclerosis, Parkinson's disease)
C4_13. Obesity
C4_14. Psoriasis
C4_15. Substance abuse

{PRG: SHOW C5 GRID IF ANY OF C4_1 - C4_13 = 1, OTHERWISE SKIP TO D1}
{DESIGN: GRID C5_1-C5_13}
{PRG: SELECT ALL THAT APPLY}

C5. Which of your blood relatives (a parent, brother or sister, child, grandparent, aunt, uncle, or first cousin) have ever had any of the following conditions?
(*Please select all that apply.*)

1 A parent
2 A brother or sister
3 A child
4 A grandparent
5 An aunt, uncle, or first cousin

{PRG: SHOW C5_1 IF C4_1 = 1}
C5_1. Arthritis
{PRG: SHOW C5_2 IF C4_2 = 1}
C5_2. Asthma
{PRG: SHOW C5_3 IF C4_3 = 1}
C5_3. Cancer
{PRG: SHOW C5_4 IF C4_4 = 1}
C5_4. Chronic kidney disease
{PRG: SHOW C5_5 IF C4_5 = 1}
C5_5. Diabetes

{PRG: SHOW C5_6 IF C4_6 = 1}
C5_6. Eye conditions
{PRG: SHOW C5_7 IF C4_7 = 1}
C5_7. Gastrointestinal (GI) conditions
{PRG: SHOW C5_8 IF C4_8 = 1}
C5_8. Heart conditions
{PRG: SHOW C5_9 IF C4_9 = 1}
C5_9. High cholesterol
{PRG: SHOW C5_10 IF C4_10 = 1}
C5_10. Lupus
{PRG: SHOW C5_11 IF C4_11 = 1}
C5_11. Mental illness/psychiatric conditions
{PRG: SHOW C5_12 IF C4_12 = 1}
C5_12. Neurological conditions (e.g. Alzheimer's disease, ALS, Multiple sclerosis, Parkinson's disease)
{PRG: SHOW C5_13 IF C4_13 = 1}
C5_13. Obesity
{PRG: SHOW C5_14 IF C4_14 = 1}
C5_14. Psoriasis
{PRG: SHOW C5_14 IF C4_15 = 1}
C5_15. Substance abuse

{Note: DK should NOT be mutually exclusive in following grids}

{PRG: SHOW C5_1a_1 - C5_1a_5 GRID IF C5_1= 1-5, OTHERWISE SKIP TO FILTER BEFORE C5_3a_1- C5_3a_5 GRID}

{DESIGN: GRID C5_1a_1 - C5_1a_5}
{PRG: C5_1a_1 - C5_1a_5 SELECT ALL THAT APPLY}

Please select the type(s) of {PRG: IF PRE_1=1 DISPLAY "arthritis"; OTHERWISE DISPLAY "arthritis/immune disorders"} that each of the following relatives has/had.
(Please select all that apply)

1 Osteoarthritis ("wear and tear" on joints)
2 Rheumatoid arthritis (joint swelling and stiffness)
3 Other
99 Don't know

{PRG: SHOW C5_1a_1 IF C5_1=1}
C5_1a_1. A parent
{PRG: SHOW C5_1a_2 IF C5_1=2}
C5_1a_2. A brother or sister
{PRG: SHOW C5p_1a_3 IF C5p_1=3}
C5_1a_3. A child
{PRG: SHOW C5_1a_4 IF C5_1=4}
C5_1a_4. A grandparent
{PRG: SHOW C5_1a_5 IF C5_1=5}
C5_1a_5. An aunt, uncle, or first cousin

{PRG: SHOW C5_3a_1 - C5_3a_5 GRID IF C5_3= 1-5, OTHERWISE SKIP TO FILTER BEFORE C5_5a_1- C5_5a_5 GRID}

{DESIGN: GRID C5_3a_1 - C5_3a_5}
{PRG: C5_3a_1 - C5_3a_5 SELECT ALL THAT APPLY}

Please select the type(s) of **cancer** that each of the following relatives has/had.
(Please select all that apply)

1	Breast cancer
2	Colorectal cancer
3	Esophageal cancer
4	Leukemia
5	Lung cancer
6	Prostate cancer
7	Skin cancer (Melanoma)
8	Stomach cancer
9	Other
99	Don't know

{PRG: SHOW C5_3a_1 IF C5_3=1}
C5_3a_1. A parent
{PRG: SHOW C5_3a_2 IF C5_3=2}
C5_3a_2. A brother or sister
{PRG: SHOW C5_3a_3 IF C5_3=3}
C5_3a_3. A child
{PRG: SHOW C5_3a_4 IF C5_3=4}
C5_3a_4. A grandparent
{PRG: SHOW C5_3a_5 IF C5_3=5}
C5_3a_5. An aunt, uncle, or first cousin

{PRG: SHOW C5_5a_1 - C5_5a_5 GRID IF C5_5 = 1-5, OTHERWISE SKIP TO FILTER BEFORE C5_6a_1- C5_6a_5 GRID}

{DESIGN: GRID C5_5a_1 - C5_5a_5}
{PRG: C5_5a_1 - C5_5a_5 SELECT ALL THAT APPLY}

Please select the type(s) of **diabetes** that each of the following relatives has/had.
(Please select all that apply)

1	Type 1 (insulin dependent, juvenile onset)
2	Type 2 (non-insulin dependent)
99	Don't know

{PRG: SHOW C5_5a_1 IF C5_5=1}
C5_5a_1. A parent
{PRG: SHOW C5_5a_2 IF C5_5=2}
C5_5a_2. A brother or sister
{PRG: SHOW C5_5a_3 IF C5_5=3}
C5_5a_3. A child
{PRG: SHOW C5_5a_4 IF C5_5=4}
C5_5a_4. A grandparent
{PRG: SHOW C5_5a_5 IF C5_5=5}
C5_5a_5. An aunt, uncle, or first cousin

{PRG: SHOW C5_6a_1 - C5_6a_5 GRID IF C5_6= 1-5, OTHERWISE SKIP TO FILTER BEFORE C5_7a_1- C5_7a_5 GRID}

{DESIGN: GRID C5_6a_1 - C5_6a_5}
{PRG: C5_6a_1 - C5_6a_5 SELECT ALL THAT APPLY}

Please select the type(s) of **eye conditions** that each of the following relatives has/had.
(Please select all that apply)

1 Age-related macular degeneration
2 Glaucoma
3 Cataracts
4 Other
99 Don't know

{PRG: SHOW C5_6a_1 IF C5_6=1}
C5_6a_1. A parent
{PRG: SHOW C5_6a_2 IF C5_6=2}
C5_6a_2. A brother or sister
{PRG: SHOW C5_6a_3 IF C5_6=3}
C5_6a_3. A child
{PRG: SHOW C5_6a_4 IF C5_6=4}
C5_6a_4. A grandparent
{PRG: SHOW C5_6a_5 IF C5_6=5}
C5_6a_5. An aunt, uncle, or first cousin

{PRG: SHOW C5_7a_1 - C5_7a_5 GRID IF C5_7 = 1-5, OTHERWISE SKIP TO FILTER BEFORE C5_8a_1- C5_8a_5 GRID}

{DESIGN: GRID C5_7a_1 - C5_7a_5}
{PRG: C5_7a_1 - C5_7a_5 SELECT ALL THAT APPLY}

Please select the type(s) of **gastrointestinal (GI) conditions** that each of the following relatives has/had.
(Please select all that apply)

1 Celiac disease
2 Crohn's disease
3 Ulcerative colitis
4 Other
99 Don't know

{PRG: SHOW C5_7a_1 IF C5_7=1}
C5_7a_1. A parent
{PRG: SHOW C5_7a_2 IF C5_7=2}
C5_7a_2. A brother or sister
{PRG: SHOW C5_7a_3 IF C5_7=3}
C5_7a_3. A child
{PRG: SHOW C5_7a_4 IF C5_7=4}
C5_7a_4. A grandparent
{PRG: SHOW C5_7a_5 IF C7_4=5}
C5_7a_5. An aunt, uncle, or first cousin

{PRG: SHOW C5_8a_1 - C5_8a_5 GRID IF C5_8 = 1-5, OTHERWISE SKIP TO FILTER BEFORE C5_11a_1- C5_11a_5 GRID}

{DESIGN: GRID C5_8a_1 - C5_8a_5}
{PRG: C5_8a_1 - C5_8a_5 SELECT ALL THAT APPLY}

Please select the type(s) of **heart conditions** that each of the following relatives has/had.
(Please select all that apply)

1 Irregular heartbeat (Atrial fibrillation)
2 Coronary artery disease
3 Peripheral arterial disease
4 Blood clotting (Venous thromboembolism)
5 Other
99 Don't know

{PRG: SHOW C5_8a_1 IF C5_8=1}
C5_8a_1. A parent
{PRG: SHOW C5_8a_2 IF C5_8=2}
C5_8a_2. A brother or sister
{PRG: SHOW C5_8a_3 IF C5_8=3}
C5_8a_3. A child
{PRG: SHOW C5_8a_4 IF C5_8=4}
C5_8a_4. A grandparent
{PRG: SHOW C5_8a_5 IF C5_8=5}
C5_8a_5. An aunt, uncle, or first cousin

{PRG: SHOW C5_11a_1 - C5_11a_5 GRID IF C5_11= 1-5, OTHERWISE SKIP TO FILTER BEFORE C5_12a_1 - C5_12a_5 GRID}

{DESIGN: GRID C5_11a_1 - C5_11a_5}
{PRG: C5_11a_1 - C5_11a_5 SELECT ALL THAT APPLY}

Please select the type(s) of **mental illness/psychiatric conditions** that each of the following relatives has/had.
(Please select all that apply)

1 Bipolar disorder
2 Depression
3 Anxiety disorder
4 Other
99 Don't know

{PRG: SHOW C5_11a_1 IF C5_11=1}
C5_11a_1. A parent
{PRG: SHOW C5_11a_2 IF C5_11=2}
C5_11a_2. A brother or sister
{PRG: SHOW C5_11a_3 IF C5_11=3}
C5_11a_3. A child
{PRG: SHOW C5_11a_4 IF C5_11=4}

C5_11a_4. A grandparent
{PRG: SHOW C5_11a_5 IF C5_11=5}
C5_11a_5. An aunt, uncle, or first cousin

{PRG: SHOW C5_12a_1 - C5_12a_5 GRID IF C5_12= 1-5, OTHERWISE SKIP TO D1}

{DESIGN: GRID C5_12a_1 - C5_12a_5}
{PRG: C5_12a_1 - C5_12a_5 SELECT ALL THAT APPLY}

Please select the type(s) of **neurological conditions** that each of the following relatives has/had.
(Please select all that apply)

1	Alzheimer's disease
2	ALS (Lou Gehrig's disease)
3	Multiple sclerosis
4	Parkinson's disease
5	Other
99	Don't know

{PRG: SHOW C5_12a_1 IF C5_12=1}
C5_12a_1. A parent
{PRG: SHOW C5_12a_2 IF C5_12=2}
C5_12a_2. A brother or sister
{PRG: SHOW C5_12a_3 IF C5_12=3}
C5_12a_3. A child
{PRG: SHOW C5_12a_4 IF C5_12=4}
C5_12a_4. A grandparent
{PRG: SHOW C5_12a_5 IF C5_12=5}
C5_12a_5. An aunt, uncle, or first cousin

{Note: Section Header: “How You’re Feeling”}

{DESIGN: GRID D1_1-D1_2}

SECT3_TEXT. Section 3 of 7: How You’re Feeling

D1. Over the **past two weeks**, how often have you:

1 Not at all
2 Several days
3 More than half of the days
4 Nearly every day

D1_1. Felt nervous, anxious, or on edge?
D1_2. Been unable to stop or control worrying?
D1_3. Felt calm and peaceful?
D1_4. Been a happy person?
D1_5. Had little interest or pleasure in doing things?
D1_6. Felt down, depressed or hopeless?

{Note: Section Header: “Risk Perceptions}

{DESIGN: GRID E1_1- E1_C2_32}

SECT4_TEXT. Section 4 of 7: Risk Perceptions

E1. Compared to the average [DISPLAY “man” IF DEM3=1 OR “woman” IF DEM3=2] of your age, what would you say your chances are of developing the conditions below sometime in the future?

Compared to the average {VALUE:CALC_E1_1-E1_C2_32_TEXT} of your age, what would you say your chances are of developing the conditions below sometime in the future?

1 Much lower than average
2 Lower than average
3 Average
4 Higher than average
5 Much higher than average
9 I have been diagnosed with this condition

E1_1. Alzheimer’s disease
{PRG: SHOW IF DEM3=2}
E1_2. Breast cancer
{PRG: SHOW IF DEM3=1}
E1_3. Prostate cancer
E1_4. Colorectal cancer
E1_5. Lung cancer
E1_6. Diabetes
E1_7. Heart disease (Coronary artery disease)
E1_8. Obesity
E1_9. Parkinson’s disease

{PRG: SHOW E1_C2_1 IF C2.1=3}
E1_C2_1 Osteoarthritis
{PRG: SHOW E1_C2_2 IF C2.2=3}
E1_C2_2 Rheumatoid arthritis
{PRG: SHOW E1_C2_3 IF C2.3=3}
E1_C2_3 Asthma
{PRG: SHOW E1_C2_6 IF C2.6=3}
E1_C2_6 Celiac disease
{PRG: SHOW E1_C2_8 IF C2.8=3}
E1_C2_8 Ulcerative colitis
{PRG: SHOW E1_C2_12 IF C2.12=3}
E1_C2_12 Leukemia
{PRG: SHOW E1_C2_15 IF C2.15=3}
E1_C2_15 Skin cancer (Melanoma)
{PRG: SHOW E1_C2_20 IF C2.20=3}
E1_C2_20 Blood clotting (Venous thromboembolism)
{PRG: SHOW E1_C2_21 IF C2.21=3}
E1_C2_21 Chronic kidney disease
{PRG: SHOW E1_C2_22 IF C2.22=3}
E1_C2_22 High cholesterol
{PRG: SHOW E1_C2_24 IF C2.24=3}

E1_C2_24	Age-related macular degeneration
{PRG: SHOW E1_C2_25 IF C2.25=3}
E1_C2_25	Glaucoma
{PRG: SHOW E1_C2_26 IF C2.26=3}
E1_C2_26	Bipolar disorder
{PRG: SHOW E1_C2_28 IF C2.28=3}
E1_C2_28	ALS (Lou Gehrig's disease)
{PRG: SHOW E1_C2_29 IF C2.29=3}
E1_C2_29	Multiple sclerosis

{Note: Section Header: “Health, Behaviors and Insurance”}

{DESIGN: GRID F1_1-F1_7}

SECT5_TEXT. Section 5 of 7: Health, Behaviors and Insurance

F1. Are you currently taking prescription medications:

1	Yes
0	No

F1_1. To thin the blood or to prevent blood clots?
F1_2. For high blood pressure or heart disease?
F1_3. For depression or anxiety?
F1_4. For diabetes?
F1_5. For high cholesterol?
{PRG: SHOW IF DEM3=2}
F1_6. For menopause symptoms?
{PRG: SHOW IF DEM3=2}
F1_7. For birth control?

{PRG: SHOW IF C3_1_5=1; OTHERWISE SKIP TO FILTER BEFORE F3}
F2. Are you currently controlling diabetes by diet?

1	Yes
0	No

{PRG: SHOW F3 IF C3_1_9=1, OTHERWISE SKIP TO F4}

F3. Are you currently controlling high cholesterol with diet?

1	Yes
0	No

F4. Are you currently taking any vitamins on a regular basis (most days)?

1	Yes
0	No

{PRG: SHOW IF F4=1; OTHERWISE SKIP TO F5}

F4a. About how many vitamin pills do you take per day?

[NUMERIC RANGE 0-30]

F5. Are you currently taking any herbal supplements?

1	Yes
0	No

{PRG: SHOW IF F5=1; OTHERWISE SKIP TO F6}

F5a. About how many herbal supplements do you take per day?

[NUMERIC RANGE 0-30]

{DESIGN: GRID F6-F7}

0	None
1	1 or less
2	2
3	3
4	4
5	5 or more

F6. On a typical day, how many servings of **fruit** do you eat? *(A serving size equals 1 piece of fruit or melon wedge, 3/4 cup of 100% juice, 1/2 cup canned fruit, or 1/4 cup dried fruit.)*

F7. On a typical day, how many servings of **vegetables** do you eat? *(A serving size equals 1/2 cup chopped raw or cooked vegetables, 1cup leafy raw vegetables, or 3/4 cup 100% vegetable juice.)*

{PRG: DISPLAY F8a AND F8b HORIZONTALLY ON SCREEN}

F8a-F8b. What is your height?

F8a. [NUMERIC RANGE 4-7] Feet F8b. [NUMERIC RANGE, 0-11, ALLOW 2 DECIMALS] Inches

---

F9. What is your weight?

[NUMERIC RANGE 70-400] Pounds

F10. Do you belong to a gym or health club?

1	Yes
0	No

{PRG: F11a_1 AS CHECKBOX}
{PRG: RESPONDENT CANNOT SELECT F11a_1 AND ENTER A RESPONSE IN F11a}

F11_TEXT. The next questions are about physical activities that you may do in your **leisure** time.

F11. How many days per week do you do **vigorous leisure-time** physical activities for **at least 10 minutes** that cause **heavy** sweating or **large** increases in breathing or heart rate?

F11a. [NUMERIC RANGE, ALLOW 0-7] day(s) per week

{PRG: SHOW IF F11a>0; OTHERWISE SKIP TO F12}

F11c. About how long do you do these vigorous leisure-time physical activities each time?

[NUMERIC RANGE, ALLOW 1-240] minutes

{PRG: F12a_1 AS CHECKBOX}
{PRG: RESPONDENT CANNOT SELECT F12a_1 AND ENTER A RESPONSE IN F12a}

F12. How many days per week do you do **light or moderate leisure-time** physical activities for **at least 10 minutes** that cause **only light** sweating or a **slight** to **moderate** increase in breathing or heart rate?

F12a. [NUMERIC RANGE; ALLOW 0-7] day(s) per week

{PRG: SHOW IF F12a>0; OTHERWISE SKIP TO F13}

F12c. About how long do you do these light or moderate leisure-time physical activities each time?

[NUMERIC RANGE, ALLOW 1-240] minutes

{PRG: F13a_1 AS CHECKBOX}
{PRG: RESPONDENT CANNOT SELECT F13a_1 AND ENTER A RESPONSE IN F13a}

F13. How many days per week do you do **leisure-time** physical activities **specifically designed to strengthen your muscles** such as lifting weights or doing calisthenics?
(Include all such activities even if you have mentioned them before.)

F13a. [NUMERIC RANGE, ALLOW 0-7] day(s) per week

{PRG: SHOW IF F13a>0; OTHERWISE SKIP TO F14}

F13c. About how long do you do these strengthening leisure-time physical activities each time?

[NUMERIC RANGE, 1-240] minutes

{PRG: SHOW IF F11a >= 3; OTHERWISE SKIP TO FILTER BEFORE F15}

F14. How long have you done **vigorous leisure-time** physical activities for 3 or more times a week?

1 I have been doing them for **more** than 6 months
2 I have been doing them for **less** than 6 months

{PRG: SHOW IF F11a < 3; OTHERWISE SKIP TO G1}

F15. Do you intend to increase your level of physical activity?

1 I intend to in the next 30 days
2 I intend to in the next 6 months
3 I do **not** intend to in the next 6 months

G1. Have you smoked at least 100 cigarettes in your entire life?

1 Yes
0 No

{PRG: SHOW IF G1=1; OTHERWISE SKIP TO H1}

G2. Do you **now** smoke cigarettes not at all, some days, or every day?

1 Not at all
2 Some days
3 Every day

{PRG: SHOW IF G2=2 OR 3; OTHERWISE SKIP TO H1}

G2a. How many cigarettes per day do you smoke?

1 10 or less
2 11-20
3 21-30
4 31 or more

H1. Blood cholesterol is a fatty substance found in the blood. Blood can be taken and used to determine your cholesterol level.

Have you had a blood test to check your cholesterol?

0 No, I've never had one
1 Yes, within the past year
2 Yes, more than a year ago
99 Don't know or not sure

H2. A blood sugar test is a blood test that measures your blood glucose or blood sugar.

Have you had a blood sugar test?

0 No, I've never had one
1 Yes, within the past year
2 Yes, more than a year ago
99 Don't know or not sure

H3. A colonoscopy is when a tube is inserted in the rectum to view the bowel for signs of cancer or other health problems. In this exam, the entire colon is checked. Anesthesia or pain medication is usually required.

Have you had a colonoscopy?

0	No, I've never had one
1	Yes, within the past year
2	Yes, more than a year ago
99	Don't know or not sure

H4. Have you had any tests in which a physician or healthcare professional looked for signs of heart disease?

0	No, I've never had these
1	Yes, within the past year
2	Yes, more than a year ago
99	Don't know or not sure

{PRG: SHOW H5 IF DEM3=2; OTHERWISE SKIP TO H7}

H5. A mammogram is an x-ray of each breast to look for early signs of breast cancer.

Have you had a mammogram?

0	No, I've never had one
1	Yes, within the past year
2	Yes, more than a year ago
99	Don't know or not sure

{PRG: SHOW H5A IF H5 = 1 OR 2 OR 99; OTHERWISE SKIP TO H6}

H5A. MRI scans use magnets and radio waves instead of x-rays to produce very detailed, cross-sectional images of the body. MRI scans can take a long time -- often up to an hour. You have to lie inside a narrow tube. For breast imaging, doctors inject a dye into a small vein in the arm before or during the exam.

Have you had a breast MRI scan?

0	No, I've never had one
1	Yes, within the past year
2	Yes, more than a year ago
99	Don't know or not sure

{PRG: SHOW IF DEM3=2; OTHERWISE SKIP TO H7}

H6. A clinical breast exam is when a physician, nurse, or other health professional feels your breasts for lumps.

Have you had a clinical breast exam?

0	No, I've never had one

1 Yes, within the past year
2 Yes, more than a year ago
99 Don't know or not sure

{PRG: SHOW IF DEM3=2; OTHERWISE SKIP TO H8}

H7. A Pap test, also called a Pap smear, is when a physician or other healthcare professional uses a special stick or brush to take a few cells from inside and around the cervix.

Have you had a Pap test?

0 No, I've never had one
1 Yes, within the past year
2 Yes, more than a year ago
99 Don't know or not sure

{PRG: SHOW H8 IF DEM3=1; OTHERWISE SKIP TO H9}

H8. A Prostate-Specific Antigen test, also called a PSA test, is a blood test used to check men for prostate cancer.

Have you had a PSA test?

0 No, I've never had one
1 Yes, within the past year
2 Yes, more than a year ago
99 Don't know or not sure

H9. Have you had any additional exams in which a physician or healthcare professional looked for signs of cancer?

0 No, I've never had these
1 Yes, within the past year
2 Yes, more than a year ago
99 Don't know or not sure

H10. In what calendar year did you have your last physical check-up?

[NUMERIC RANGE 1950-2012]

{PRG: SHOW H11 IF H10 = 2011 OR 2012; OTHERWISE SKIP TO J1}

H11. In the past year, how many visits with a physician or healthcare provider have you had? (Please do **not** include visits with a mental health professional such as a psychiatrist or psychotherapist.)

[NUMERIC RANGE 0-100] visits in the past year

J1. Do you have health insurance?

1 Yes
0 No
99 Don’t know

{PRG: SHOW IF J1=1; OTHERWISE SKIP TO J2}
{PRG: SELECT ALL THAT APPLY}

J1a. Is your insurance through:
(Please select all that apply)

1 Employer
2 Spouse’s employer
3 Self-purchased
4 Medicare/Medicaid
5 Parent’s employer
99 Don’t know

J2-J4. Do you have…

1 Yes
0 No
99 Don’t know

J2. Life insurance?
J3. Disability insurance?
J4. Long term care insurance?

{PRG: SHOW J3a IF J3=1, OTHERWISE SKIP TO J4}

J3a. Is your disability insurance short term, long term, or both?

1 Short term
2 Long term
3 Both short and long term
99 Don’t know

{Note: Section Header: “Genetics and Numbers”}

{DESIGN: GRID L1_1-L1_9}

SECT6_TEXT. Section 6 of 7: Genetics and Numbers

L1. Please indicate whether you think the following statements are true or false.

1 True
0 False

L1_1. Healthy parents can have a child with an inherited disease
L1_2. If your close relatives have diabetes or heart disease, you are more likely to develop these conditions
L1_3. Some genetic disorders occur more often within particular ethnic groups
L1_4. Most genetic disorders are caused by only a single gene
L1_5. Once a genetic marker for a disorder is identified in a person, the disorder can usually be prevented or cured
L1_6. A disease is only genetically determined if more than one family member is affected
L1_7. Some of the genetic disorders occur later in adult life
L1_8. A healthy lifestyle can prevent or lessen the negative consequences of having genetic predispositions to some diseases
L1_9. The environment has little or no effect on how genes contribute to disease

---

{DESIGN: GRID L2_1-L2_5}

L2_1-L2_5. How much do you agree or disagree with the following statements?

1 Strongly disagree
2 Disagree
3 Somewhat disagree
4 Neither agree nor disagree
5 Somewhat agree
6 Agree
7 Strongly agree

L2_1. I am confident in my ability to understand information about genetics.
L2_2. I am able to understand information about how genes can affect my health.
L2_3. I have a good idea about how genetics may influence risk for disease generally.
L2_4. I have a good idea about how my own genetic make-up might affect my risk for disease.
L2_5. I am able to explain to others how genes affect one’s health.

M1. How much do you agree or disagree with the following statement?

In general, I depend on numbers and statistics to help me make decisions about my health.

1 Strongly disagree
2 Somewhat disagree
3 Somewhat agree
4 Strongly agree

M2. Which of the following numbers represents the biggest risk of getting a disease?

1	1 in 100
2	1 in 1000
3	1 in 10

M3. If person A's risk of getting a disease is 1% in ten years, and person B's risk is double that of person A's, what is B's risk?

Person B's risk is: [NUMERIC RESPONSE, 0-100] %

M4. If the chance of getting a disease is 10%, how many people would be expected to get the disease:

M4_1. Out of 100 people?	[NUMERIC RESPONSE, 0-100]
M4_2. Out of 1000 people?	[NUMERIC RESPONSE, 0-1000]

M5. The chance of getting a viral infection is .0005. Out of 10,000 people, about how many of them are expected to get infected?

[NUMERIC RESPONSE, 0-10000] people

{Note: Section Header: “About You”}

SECT7_TEXT. Section 7 of 7: About You

N1. Have you ever met clinically with a genetic counselor or a doctor who specializes in genetics?

1 Yes
0 No
99 Unsure

N2. Have you had any genetic testing done in the past (other than newborn screening)?

1 Yes
0 No
99 Unsure

{PRG: SHOW IF N2=1; OTHERWISE SKIP TO K1}
{PRG: N2a SELECT ALL THAT APPLY}

N2a. What type of genetic testing did you have done?
(Please select all that apply)

1 Carrier testing (e.g., Tay-Sachs, cystic fibrosis, thalassemia, sickle cell anemia)
2 Diagnostic genetic testing (to diagnose or rule-out a specific genetic condition)
3 Prenatal genetic testing (e.g., chorionic villus sampling, amniocentesis)
4 Predictive or presymptomatic genetic testing (e.g., to learn your risk of a certain disease or condition)
5 Testing for medication response or medication treatment tailoring
6 Ancestry genetic testing
7 Trait genetic testing (e.g. athletic ability)
8 Nutrigenomic testing
9 Other *(Please specify)* [TEXT RESPONSE]

K1. Are you adopted?

1 Yes
0 No

K2. What is your marital status?

1 Single
2 Married
3 Widowed
4 Divorced/separated
5 In a long-term relationship or living with a partner

K3. Do you have any biological children?

1 Yes
0 No

{PRG: SHOW K3a if K3=1, OTHERWISE SKIP TO K4}

K3a. How many biological children do you have?

[NUMERIC RESPONSE, 0-30]

K3b. Are any under age 18?

1 Yes
0 No

K4. Do you consider yourself to be Hispanic or Latino?

1 Yes
0 No

{PRG: K5SELECT ALL THAT APPLY}

K5. How do you describe your race?
(*Please select all that apply*.)

1 American Indian/Native Alaskan
2 Asian
3 Black or African American
4 Hawaiian or Pacific Islander
5 White
6 Other (*Please specify):* [TEXT RESPONSE]

K6. What is the highest level of education you have completed?

1 No formal education
2 Grade school
3 High school diploma or GED
4 Some college
5 College degree
6 Some graduate school
7 Master's degree
8 Some doctoral work
9 Doctorate degree (e.g., PhD, DSc, EdD)
10 Doctor of Medicine (MD)
11 Other doctorate-equivalent professional degree (e.g., JD, LLB, DDS, DVM)

{PRG: K7 SELECT ALL THAT APPLY}

K7. What is your current employment status?
(Please select all that apply.)

1	Full-time
2	Part-time
3	Retired
4	Self-employed
5	Unemployed
6	Student
7	Not working by choice

K8. In which field is your current occupation (or previous occupation if retired)?

1	Business, Financial, Management, Sales and Related Occupations
2	Computer, Engineering and Mathematical Science
3	Life, Physical, and Social Science
4	Legal
5	Education, Training, and Library
6	Arts, Design, Entertainment, Sports, and Media
7	Healthcare Practitioner
8	Office and Administrative Support
9	Construction, Maintenance, and Natural Resources
10	Production and Transportation
11	Other *(Please specify):* [TEXT RESPONSE]

K9. What is your household's total combined income during the past 12 months?
(This includes money from pensions, social security payments, jobs, net income from business, farm or rent, dividends, interest and any other income received by family members who are 15 years of age or older.)

1	<$40,000
2	$40,000 – $69,999
3	$70,000 – $99,999
4	$100,000 – $199,999
5	$200,000 – $500,000
6	>$500,000

K10. Please select the statement that best describes the role you have actually taken in dealing with your healthcare:

1	I prefer to make the final selection about which treatment I will receive.
2	I prefer to make the final selection of my treatment after seriously considering my doctor's opinion.
3	I prefer that my doctor and I share responsibility for deciding which treatment is best for me.
4	I prefer that my doctor make the final decision about which treatment will be used, but seriously considers my opinion.
5	I prefer to leave all decisions regarding my treatment to my doctor.

{PRG: NO SOFT PROMPT ON K11}

K11. Would you say that in general your health is:

1	Excellent
2	Very good
3	Good
4	Fair
5	Poor
