# Supplementary material for: Design, methods, and participant characteristics of the Impact of Personal Genomics (PGen) Study, a prospective cohort study of direct-to-consumer personal genomic testing customers
Source: Genome Med. 2014 Dec 3;6(12):96. doi: 10.1186/s13073-014-0096-0 (PMC4256737; doi:10.1186/s13073-014-0096-0)
Supplement: Additional file 2: — 2 week follow-up PGen Study survey. [file 13073_2014_96_MOESM2_ESM.pdf]

2-3 Week Follow Up Questionnaire Specifications / S10028 (D9_SK)

Short URL to direct towards production survey	https://www.ssgresearch.com/pgen2
Support email address to include in header	pgen@ssgresearch.com
Support phone number to include in header (if needed)	

Logo to use if other than SSG logo
Please list network location of other logo to use:

Mandatoriness (check the appropriate setting)

	All questions are optional unless otherwise noted
x	All questions are optional with a soft prompt included if no answer is provided
	All questions are mandatory

Please provide text to use for Mandatoriness prompt if being used (Default text to use is provided below):
General:

We noticed that you did not answer a question on the previous page. It is important to us that we get a complete set of responses from you. Please return to the previous page by clicking "Previous" and select an answer for each question. If you would rather not select an answer, you may instead continue to the next page by clicking "Next."

Other specify:

You selected 'Other' but did not specify your answer. Please return to the last question by clicking “Previous” and type in your specific answer. If you would rather not specify an answer, you may instead continue to the next page by clicking “Next.”

Header Sections (if being used)

Section Label	Questions in Section	Section Label	Questions in Section
How You’re Feeling	A1a-A1f		
Reactions to Genetic Results	A3-A6h		
Sample Results Scenarios	Q1a-Q4c_d		
Utility of Genetic Results	P1-P8		

Survey Title to appear in header (appears above the section header bar)

The Impact of Personal Genomics (PGen) Study

Welcome Page text (please modify the following as needed)

Welcome to the Impact of Personal Genomics (PGen) Study!
This is the second of three surveys for this study.

Please enter your User ID, then click START SURVEY to begin! If you do not know what your ID is, please email pgen@ssgresearch.com.

User ID _______________

Resume Page text (please modify the following as needed)

Thank you for returning to the survey. Please click “RESUME SURVEY” to begin where you last left off…

End Page Text
{SHOW END 1 IF QUAL1=1}
END1.

Thank you for your participation! This concludes the second study survey.

Within the next 2 weeks, you will receive a $20 Amazon.com gift certificate via email.

Six months after you receive your 23andMe results, we will send you an email with a link to the third study survey.

You may now close your browser.

{SHOW END2 IF QUAL1≠1 AND PRE_3=1}
END2.

{PRG: SHOW IF PRE_3=1}
This survey is appropriate to complete after viewing your health-related genetic results from {DISPLAY PRE_1}. Thank you for your interest in this research study!

{SHOW END2 IF QUAL1≠1 AND PRE_3=2}
END3
{PRG: SHOW IF PRE_3=2}
This survey is meant for individuals who choose to view their health-related genetic results from {DISPLAY PRE_1}. Since you have chosen not to view your health-related results, you will not receive any further emails about this research study. We thank you again for your interest!

Survey Title appearing in browser window

PGen Study

GENERAL PROGRAMMING NOTES

All emphasis should be programmed in black, all caps text instead of lowercase blue text.

Preloads

PRE_1 Genetics Company
1 Pathway Genomics
2 23andMe

{Note: Preload from baseline survey}
PRE_2 Gender
1 Male
2 Female

PRE_3. Instance of survey
1 Attempt 1
2 Attempt 2

{PRG: PRE_C2 AS MULTI-VALUE, WILL PRELOAD BASED ON SELECTIONS FROM BASELINE SURVEY}
PRE_C2. Conditions of interest from baseline survey

1 Osteoarthritis
2 Rheumatoid arthritis
3 Asthma
6 Celiac disease
8 Ulcerative colitis
9 Breast cancer (females only)
10 Colorectal cancer
12 Leukemia
13 Lung Cancer
14 Prostate cancer (males only)
15 Skin cancer (Melanoma)
17 Heart disease (Coronary artery disease)
20 Blood clotting (Venous thromboembolism)
21 Chronic kidney disease
22 High cholesterol
23 Diabetes
24 Age-related macular degeneration
25 Glaucoma
26 Bipolar disorder
27 Alzheimer's disease
28 ALS (Lou Gehrig's disease)
29 Multiple sclerosis
30 Parkinson's disease
31 Obesity

RANDOM2.
1 PKU scenario (3b, 3c)/ [do not show CF scenario)
2 CF scenario (3e, 3f)/ [do not show PKU scenario)

Note on Images:

Everyone gets increased risk of AD
Everyone gets decreased genetic risk of diabetes
Everyone gets carrier screening list
Randomize to either PKU carrier result, or negative CF screening result
Everyone gets increased risk of statin-related myopathy.

INTRO_1: Section 1 of 4: How You're Feeling
{PRG: QUAL1 SELECT ALL THAT APPLY}

QUAL1. Which of the following types of genetic results from {DISPLAY PRE_1} have you viewed?
(Select all that apply)

1 Your health-related results
{PRG: SHOW QUAL1.3 IF PRE_1=2}
2 Your ancestry/genealogy results {show for 23andMe only}
{PRG: SHOW QUAL1.3 IF PRE_1=2}
3 Your traits {show for 23andMe only}
4 I have not viewed my genetic results from {DISPLAY PRE_1}

{PRG: IF QUAL1 ≠ 1, JUMP TO END2}
{PRG (DISCOVERY): IF QUAL1 ≠ 1 ADD ONE ADDITIONAL INSTANCE OF SURVEY, SEND INVITATION AFTER TWO WEEKS}

After 2 weeks have passed, if respondent did not select "Your health-related results" AND participant has not yet completed the survey, send another email invitation for 2-3 week survey, which presents the following question again:

{PRG: SHOW QUAL2 IF PRE_3=2 AND QUAL1 ≠ 1, OTHERWISE SKIP TO FILTER BEFORE A1}
{PRG: QUAL2 SELECT ALL THAT APPLY}

QUAL2. What are your reasons for not viewing your health-related genetic results from {Display:PRE_1]?
(Select all that apply)

1 I am not interested in my health-related genetic information
2 I am not sure if I want to know my health-related genetic information
3 I have not had time to review my health-related genetic results
4 Other *(Please specify)* [TEXT RESPONSE]

{PRG: SHOW A1 IF QUAL1 = 1, OTHERWISE SKIP TO END2}

{DESIGN: GRID A1a-A1f}

A1. Over the **past two weeks**, how often have you:

1 Not at all
2 Several days
3 More than half of the days
4 Nearly every day

A1a. Felt nervous, anxious, or on edge?
A1b. Been unable to stop or control worrying?
A1c. Felt calm and peaceful?
A1d Been a happy person?
A1e. Had little interest or pleasure in doing things?
A1f. Felt down, depressed or hopeless?

{NOTE: SECTION HEADER "Reactions to Genetic Results"}

INTRO_2: Section 2 of 4: Reactions to Genetic Results

{PRG: NO SOFT PROMPT ON A4}

A3_TEXT. Please read the following statements and select the response that best applies to you.

{DESIGN: GRID A3}
{PRG: SAME SCREEN A3 AND A4}

A3. Since receiving my genetic results from {DISPLAY PRE_1}, I have…

0 Never
1 Rarely
2 Sometimes
3 Often

A3l. Felt surprised about my results
A3m. Felt disappointed about my results
A3a. Felt upset about my results
A3b. Felt relieved about my results
A3c. Felt happy about my results
A3d. Felt motivated to change my lifestyle because of my results
A3e. Worried about my risk of getting diseases
A3f. Been uncertain about what my results mean for my risk of developing diseases
A3g. Been uncertain about what my results mean for my child(ren)'s and/or family's disease risk
A3h. Felt unsure about what to do to prevent diseases
A3i. Felt concerned about how my results will affect my insurance status
A3j. Had difficulty talking about my results with others
A3k. Wanted to tell others about my results

{PRG: A4 and A4a ON SAME PAGE}

A4. Which result or results were most important to you?

[OPEN END RESPONSE]

A4a. Please explain why this result or results were important to you.

[OPEN END RESPONSE]

{DESIGN: GRID A5_1-A5_m24}

A5. Compared to the average [DISPLAY "man" IF PRE_2=1 OR "woman" IF PRE_2=2] of your age, what would you say your chances are of developing the conditions below sometime in the future?

1 Much lower than average
2 Lower than average
3 Average
4 Higher than average
5 Much higher than average
6 I already have this condition

A5_1. Alzheimer's disease
{PRG: SHOW IF PRE_2=2}
A5_2. Breast cancer
{PRG: SHOW IF PRE_2=1}
A5_3. Prostate cancer
A5_4. Colorectal cancer
A5_5. Lung cancer
A5_6. Diabetes
A5_7. Heart disease (Coronary artery disease)
A5_8. Obesity
A5_9. Parkinson's disease
{PRG: SHOW A5_C2_1 IF PRE_C2 = 1}

A5_C2_1 Osteoarthritis
{PRG: SHOW A5_C2_2 IF PRE_C2 = 2}
A5_C2_2 Rheumatoid arthritis
{PRG: SHOW A5_C2_3 IF PRE_C2 = 3}
A5_C2_3 Asthma
{PRG: SHOW A5_C2_6 IF PRE_C2 = 6}
A5_C2_6 Celiac disease
{PRG: SHOW A5_C2_8 IF PRE_C2 = 8}
A5_C2_8 Ulcerative colitis
{PRG: SHOW A5_C2_12 IF PRE_C2 = 12}
A5_C2_12 Leukemia
{PRG: SHOW A5_C2_15 IF PRE_C2 = 15}
A5_C2_15 Skin cancer (Melanoma)
{PRG: SHOW A5_C2_20 IF PRE_C2 = 20}
A5_C2_20 Blood clotting (Venous thromboembolism)
{PRG: SHOW A5_C2_21 IF PRE_C2 = 21}
A5_C2_21 Chronic kidney disease
{PRG: SHOW A5_C2_22 IF PRE_C2 = 22}
A5_C2_22 High cholesterol
{PRG: SHOW A5_C2_24 IF PRE_C2 = 24}
A5_C2_24 Age-related macular degeneration
{PRG: SHOW A5_C2_25 IF PRE_C2 = 25}
A5_C2_25 Glaucoma
{PRG: SHOW A5_C2_26 IF PRE_C2 = 26}
A5_C2_26 Bipolar disorder
{PRG: SHOW A5_C2_28 IF PRE_C2 = 28}
A5_C2_28 ALS (Lou Gehrig's disease)
{PRG: SHOW A5_C2_29 IF PRE_C2 = 29}
A5_C2_29 Multiple sclerosis

{DESIGN: GRID A6a-A6h}

A6. Out of all the genetic results you received from {DISPLAY PRE_1}, how many were of the following types?

0 None of them
1 A few of them
2 Many of them
3 All of them
99 Don't know or not sure

A6a. Results you found interesting
A6b. Results showing you are at higher than average risk for a certain condition
A6c. Results showing you are at lower than average risk for a certain condition
A6d. Results that show you have average or near average risks for a certain condition
A6e. Results showing that you are a carrier for a genetic condition/disease
A6f. Results that show you may respond differently to certain medications
A6g. Results you can use to improve your health
A6h. Results you don't understand

{NOTE: SECTION HEADER “Sample Results Scenarios”}

{PRG: INTRO_3 TEXT ON ITS OWN SCREEN}
NTRO_3:
Section 3 of 4: Sample Results Scenarios

On the next few screens, we’re going to show you some examples of results from {DISPLAY PRE_1} and ask you a series of questions based on the results.

SAMPLE1_TEXT. Imagine your friend Lindsay received the following information about her risk of developing Alzheimer’s disease. Your friend Lindsay has European ancestry and she is 55 years old.

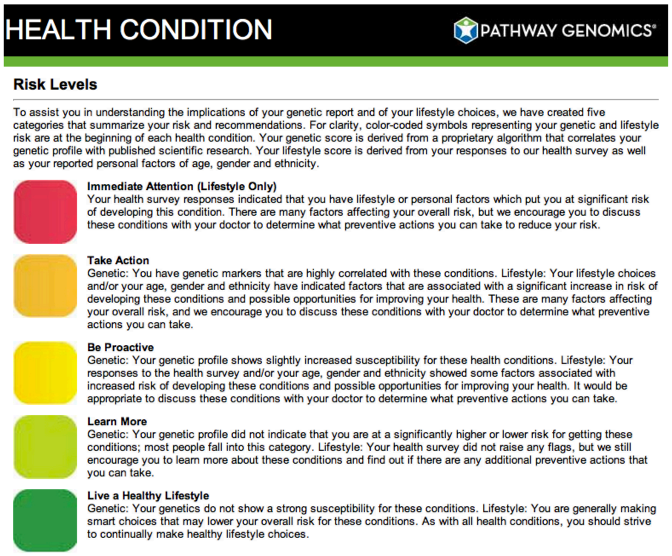

HEALTH CONDITION — PATHWAY GENOMICS®

Risk Levels

To assist you in understanding the implications of your genetic report and of your lifestyle choices, we have created five categories that summarize your risk and recommendations. For clarity, color-coded symbols representing your genetic and lifestyle risk are at the beginning of each health condition. Your genetic score is derived from a proprietary algorithm that correlates your genetic profile with published scientific research. Your lifestyle score is derived from your responses to our health survey as well as your reported personal factors of age, gender and ethnicity.

Immediate Attention (Lifestyle Only)
Your health survey responses indicated that you have lifestyle or personal factors which put you at significant risk of developing this condition. There are many factors affecting your overall risk, but we encourage you to discuss these conditions with your doctor to determine what preventive actions you can take to reduce your risk.

Take Action
Genetic: You have genetic markers that are highly correlated with these conditions. Lifestyle: Your lifestyle choices and/or your age, gender and ethnicity have indicated factors that are associated with a significant increase in risk of developing these conditions and possible opportunities for improving your health. These are many factors affecting your overall risk, and we encourage you to discuss these conditions with your doctor to determine what preventive actions you can take.

Be Proactive
Genetic: Your genetic profile shows slightly increased susceptibility for these health conditions. Lifestyle: Your responses to the health survey and/or your age, gender and ethnicity showed some factors associated with increased risk of developing these conditions and possible opportunities for improving your health. It would be appropriate to discuss these conditions with your doctor to determine what preventive actions you can take.

Learn More
Genetic: Your genetic profile did not indicate that you are at a significantly higher or lower risk for getting these conditions; most people fall into this category. Lifestyle: Your health survey did not raise any flags, but we still encourage you to learn more about these conditions and find out if there are any additional preventive actions that you can take.

Live a Healthy Lifestyle
Genetic: Your genetics do not show a strong susceptibility for these conditions. Lifestyle: You are generally making smart choices that may lower your overall risk for these conditions. As with all health conditions, you should strive to continually make healthy lifestyle choices.

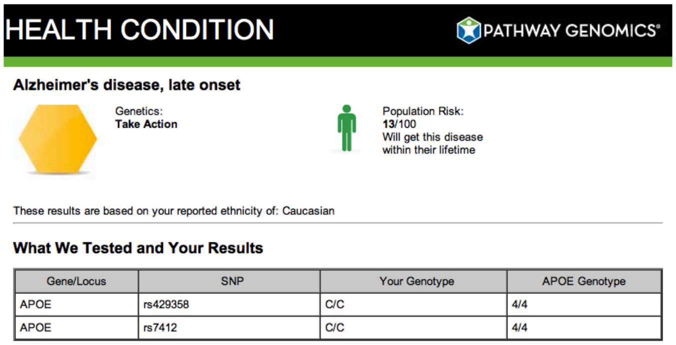

HEALTH CONDITION — PATHWAY GENOMICS®

Alzheimer's disease, late onset

Genetics:
Take Action

Population Risk:
13/100
Will get this disease within their lifetime

These results are based on your reported ethnicity of: Caucasian

What We Tested and Your Results

Gene/Locus	SNP	Your Genotype	APOE Genotype
APOE	rs429358	C/C	4/4
APOE	rs7412	C/C	4/4

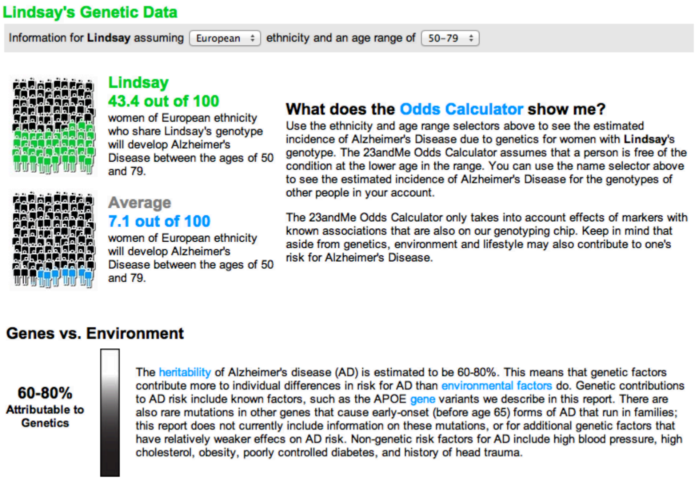

Lindsay's Genetic Data

Information for **Lindsay** assuming European ethnicity and an age range of 50–79

Lindsay
43.4 out of 100
women of European ethnicity who share Lindsay's genotype will develop Alzheimer's Disease between the ages of 50 and 79.

Average
7.1 out of 100
women of European ethnicity will develop Alzheimer's Disease between the ages of 50 and 79.

What does the Odds Calculator show me?
Use the ethnicity and age range selectors above to see the estimated incidence of Alzheimer's Disease due to genetics for women with **Lindsay's** genotype. The 23andMe Odds Calculator assumes that a person is free of the condition at the lower age in the range. You can use the name selector above to see the estimated incidence of Alzheimer's Disease for the genotypes of other people in your account.

The 23andMe Odds Calculator only takes into account effects of markers with known associations that are also on our genotyping chip. Keep in mind that aside from genetics, environment and lifestyle may also contribute to one's risk for Alzheimer's Disease.

Genes vs. Environment

60-80%
Attributable to Genetics

The heritability of Alzheimer's disease (AD) is estimated to be 60-80%. This means that genetic factors contribute more to individual differences in risk for AD than environmental factors do. Genetic contributions to AD risk include known factors, such as the APOE gene variants we describe in this report. There are also rare mutations in other genes that cause early-onset (before age 65) forms of AD that run in families; this report does not currently include information on these mutations, or for additional genetic factors that have relatively weaker effects on AD risk. Non-genetic risk factors for AD include high blood pressure, high cholesterol, obesity, poorly controlled diabetes, and history of head trauma.

{PRG: SAME SCREEN Q1a-Q1d}

Q1a. Based on these results, what are Lindsay’s chances of developing Alzheimer’s disease compared to the average woman of her age and ethnicity?

1 Much higher
2 Somewhat higher
3 About the same
4 Somewhat lower
5 Much lower

Q1d. Based on her results, which of the following statements best describes Lindsay's chances of developing Alzheimer's disease?

{if company is 23andMe}
1 She will almost certainly develop Alzheimer's disease
2 She has a 43% chance of developing Alzheimer's disease by age 79
3 She has a 7% chance of developing Alzheimer's disease by age 79
4 She will definitely NOT develop Alzheimer's disease

{if company is Pathway}
1 She will almost certainly develop Alzheimer's disease
2 She has a greater than 13% chance of developing Alzheimer's disease
3 She has a 13% chance of developing Alzheimer's disease
4 She will definitely NOT develop Alzheimer's disease

{PRG: INTRO_TEXT TO APPEAR ABOVE IMAGES}

Still thinking about Lindsay's results…

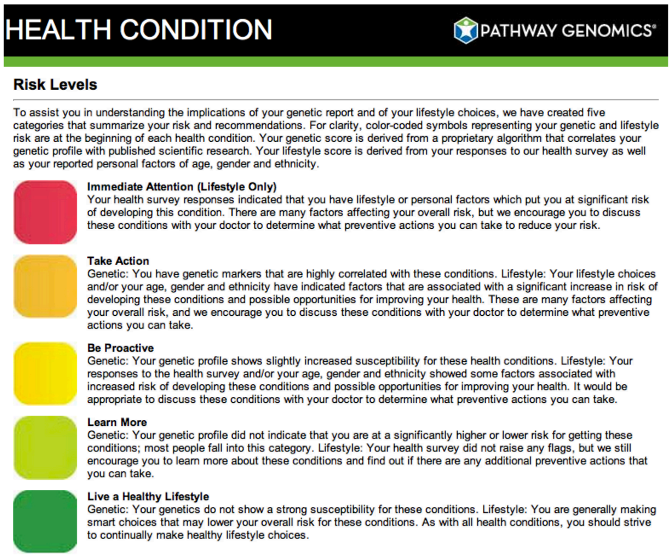

HEALTH CONDITION

PATHWAY GENOMICS®

Risk Levels

To assist you in understanding the implications of your genetic report and of your lifestyle choices, we have created five categories that summarize your risk and recommendations. For clarity, color-coded symbols representing your genetic and lifestyle risk are at the beginning of each health condition. Your genetic score is derived from a proprietary algorithm that correlates your genetic profile with published scientific research. Your lifestyle score is derived from your responses to our health survey as well as your reported personal factors of age, gender and ethnicity.

Immediate Attention (Lifestyle Only)
Your health survey responses indicated that you have lifestyle or personal factors which put you at significant risk of developing this condition. There are many factors affecting your overall risk, but we encourage you to discuss these conditions with your doctor to determine what preventive actions you can take to reduce your risk.

Take Action
Genetic: You have genetic markers that are highly correlated with these conditions. Lifestyle: Your lifestyle choices and/or your age, gender and ethnicity have indicated factors that are associated with a significant increase in risk of developing these conditions and possible opportunities for improving your health. These are many factors affecting your overall risk, and we encourage you to discuss these conditions with your doctor to determine what preventive actions you can take.

Be Proactive
Genetic: Your genetic profile shows slightly increased susceptibility for these health conditions. Lifestyle: Your responses to the health survey and/or your age, gender and ethnicity showed some factors associated with increased risk of developing these conditions and possible opportunities for improving your health. It would be appropriate to discuss these conditions with your doctor to determine what preventive actions you can take.

Learn More
Genetic: Your genetic profile did not indicate that you are at a significantly higher or lower risk for getting these conditions; most people fall into this category. Lifestyle: Your health survey did not raise any flags, but we still encourage you to learn more about these conditions and find out if there are any additional preventive actions that you can take.

Live a Healthy Lifestyle
Genetic: Your genetics do not show a strong susceptibility for these conditions. Lifestyle: You are generally making smart choices that may lower your overall risk for these conditions. As with all health conditions, you should strive to continually make healthy lifestyle choices.

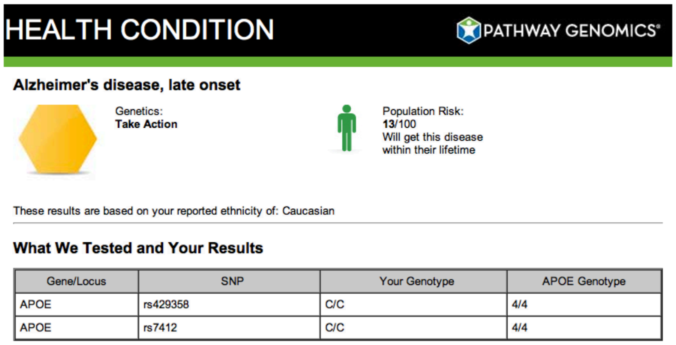

HEALTH CONDITION

PATHWAY GENOMICS®

Alzheimer's disease, late onset

Genetics:
Take Action

Population Risk:
13/100
Will get this disease within their lifetime

These results are based on your reported ethnicity of: Caucasian

What We Tested and Your Results

Gene/Locus	SNP	Your Genotype	APOE Genotype
APOE	rs429358	C/C	4/4
APOE	rs7412	C/C	4/4

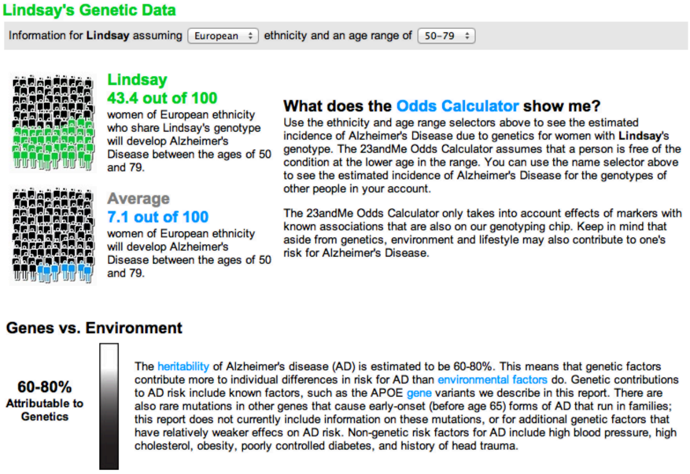

Lindsay's Genetic Data

Information for **Lindsay** assuming European ethnicity and an age range of 50-79

Lindsay
43.4 out of 100
women of European ethnicity who share Lindsay's genotype will develop Alzheimer's Disease between the ages of 50 and 79.

Average
7.1 out of 100
women of European ethnicity will develop Alzheimer's Disease between the ages of 50 and 79.

What does the Odds Calculator show me?
Use the ethnicity and age range selectors above to see the estimated incidence of Alzheimer's Disease due to genetics for women with **Lindsay**'s genotype. The 23andMe Odds Calculator assumes that a person is free of the condition at the lower age in the range. You can use the name selector above to see the estimated incidence of Alzheimer's Disease for the genotypes of other people in your account.

The 23andMe Odds Calculator only takes into account effects of markers with known associations that are also on our genotyping chip. Keep in mind that aside from genetics, environment and lifestyle may also contribute to one's risk for Alzheimer's Disease.

Genes vs. Environment

60-80%
Attributable to Genetics

The heritability of Alzheimer's disease (AD) is estimated to be 60-80%. This means that genetic factors contribute more to individual differences in risk for AD than environmental factors do. Genetic contributions to AD risk include known factors, such as the APOE gene variants we describe in this report. There are also rare mutations in other genes that cause early-onset (before age 65) forms of AD that run in families; this report does not currently include information on these mutations, or for additional genetic factors that have relatively weaker effecs on AD risk. Non-genetic risk factors for AD include high blood pressure, high cholesterol, obesity, poorly controlled diabetes, and history of head trauma.

{DESIGN: GRID Q1c CHECK ALL THAT APPLY}
{PRG: Q1c_e mutually exclusive}

Q1c. Based on these results, what should Lindsay do next?
(Please select all that apply.)

Q1c_a. Try to engage more in mental exercises (e.g., Sudoku, crossword puzzles)
Q1c_b. Discuss these results with a physician
Q1c_c. Review or make changes to her insurance and/or financial plans
Q1c_d. Review or make changes to her living will, power of attorney or other advance directives
Q1c_e. No actions are necessary based on Lindsay's results

SAMPLE2.TEXT.

Now for the next scenario…

Imagine your friend Dan received the following information about his risk for type 2 diabetes. Dan is a 35-year-old man of European ancestry who is 5 feet 8 inches tall and weighs 210 lbs. His doctor told him that according to his Body Mass Index or BMI, he is considered to be obese. Dan does not currently have type 2 diabetes.

HEALTH CONDITION

PATHWAY GENOMICS®

Risk Levels

To assist you in understanding the implications of your genetic report and of your lifestyle choices, we have created five categories that summarize your risk and recommendations. For clarity, color-coded symbols representing your genetic and lifestyle risk are at the beginning of each health condition. Your genetic score is derived from a proprietary algorithm that correlates your genetic profile with published scientific research. Your lifestyle score is derived from your responses to our health survey as well as your reported personal factors of age, gender and ethnicity.

Immediate Attention (Lifestyle Only)
Your health survey responses indicated that you have lifestyle or personal factors which put you at significant risk of developing this condition. There are many factors affecting your overall risk, but we encourage you to discuss these conditions with your doctor to determine what preventive actions you can take to reduce your risk.

Take Action
Genetic: You have genetic markers that are highly correlated with these conditions. Lifestyle: Your lifestyle choices and/or your age, gender and ethnicity have indicated factors that are associated with a significant increase in risk of developing these conditions and possible opportunities for improving your health. These are many factors affecting your overall risk, and we encourage you to discuss these conditions with your doctor to determine what preventive actions you can take.

Be Proactive
Genetic: Your genetic profile shows slightly increased susceptibility for these health conditions. Lifestyle: Your responses to the health survey and/or your age, gender and ethnicity showed some factors associated with increased risk of developing these conditions and possible opportunities for improving your health. It would be appropriate to discuss these conditions with your doctor to determine what preventive actions you can take.

Learn More
Genetic: Your genetic profile did not indicate that you are at a significantly higher or lower risk for getting these conditions; most people fall into this category. Lifestyle: Your health survey did not raise any flags, but we still encourage you to learn more about these conditions and find out if there are any additional preventive actions that you can take.

Live a Healthy Lifestyle
Genetic: Your genetics do not show a strong susceptibility for these conditions. Lifestyle: You are generally making smart choices that may lower your overall risk for these conditions. As with all health conditions, you should strive to continually make healthy lifestyle choices.

HEALTH CONDITION

PATHWAY GENOMICS®

Diabetes, type 2

Genetics: **Live a Healthy Lifestyle**

Lifestyle: **Immediate Attention**

Population Risk: **33.9** /100
Will get this disease within their lifetime

These results are based on your reported ethnicity of: Caucasian

What We Tested and Your Results

Gene/Locus	SNP	Your Genotype	Odds Ratio	Associated Allele	Population Frequency	Validated Marker	PMID
CDKAL1	rs10946398	A/A	1.00	C	34%	Validated	17463249
CDKN2B	rs10811661	C/T	1.20	T	80%	Validated	17463246
HHEX	rs1111875	A/A	1.00	G	57%	Validated	17463246
IGF2BP2	rs1470579	A/C	1.19	C	30%	Validated	17463246
KCNJ11	rs5219	C/C	1.00	T	36%	Validated	17463246
KCNQ1	rs2237892	C/C	1.66	C	93%	Validated	18711367
MTNR1B	rs10830963	G/G	1.19	G	30%	Validated	19060907
PPARG	rs1801282	C/G	1.23	C	90%	Validated	17463249
SLC30A8	rs13266634	T/T	1.00	C	76%	Validated	17463249
TCF7L2	rs7903146	C/C	1.00	T	28%	Validated	17463246

What Should I Do?

Your genetic profile does not show susceptibility for type 2 diabetes. Other factors, such as lifestyle choices, may play a larger role in whether or not you get type 2 diabetes. Stay healthy with a low-fat diet, plenty of exercise, and routine visits to your doctor.

Dan's Genetic Data

Information for **Dan** assuming European ethnicity and an age range of 20–79

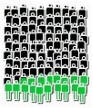

Dan
17.8 out of 100
men of European ethnicity who share Dan's genotype will develop Type 2 Diabetes between the ages of 20 and 79.

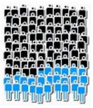

Average
25.7 out of 100
men of European ethnicity will develop Type 2 Diabetes between the ages of 20 and 79.

What does the Odds Calculator show me?
Use the ethnicity and age range selectors above to see the estimated incidence of Type 2 Diabetes due to genetics for men with **Dan's** genotype. The 23andMe Odds Calculator assumes that a person is free of the condition at the lower age in the range. You can use the name selector above to see the estimated incidence of Type 2 Diabetes for the genotypes of other people in your account.

The 23andMe Odds Calculator only takes into account effects of markers with known associations that are also on our genotyping chip. Keep in mind that aside from genetics, environment and lifestyle may also contribute to one's chances of developing type 2 diabetes.

Genes vs. Environment

26%
Attributable to Genetics

The heritability of type 2 diabetes is estimated to be 26%. This means that environmental factors contribute more to differences in risk for this condition than genetic factors. Genetic factors that play a role in type 2 diabetes include both unknown factors and known factors such as the SNPs we describe here. Environmental factors include obesity, gestational diabetes, giving birth to at least one baby weighing nine pounds or more, high blood pressure, abnormal cholesterol levels, physical inactivity, polycystic ovarian syndrome, other clinical conditions associated with insulin resistance, a history of impaired glucose tolerance or impaired fasting glucose, and a history of cardiovascular disease.

{PRG: SAME SCREEN Q2a-Q2e}

Q2a. Based on his GENETIC results, what are Dan's chances of developing diabetes compared to the average man of his age and ethnicity?

1 Much higher
2 Somewhat higher
3 About the same
4 Somewhat lower
5 Much lower

Q2d. Based on his GENETIC results, do you think that Dan:

1 Definitely will develop diabetes
2 Probably will develop diabetes
3 Probably will NOT develop diabetes
4 Definitely will NOT develop diabetes

Q2e. Which of the following is a true statement about Dan's risk of diabetes?

1 Dan's obesity is an IMPORTANT risk factor for diabetes regardless of his genetic results
2 Dan's obesity is LESS of a risk factor for diabetes because of his genetic results
3 Dan's obesity is NOT a risk factor for diabetes

INTRO_TEXT2. Still thinking about Dan's results…

HEALTH CONDITION

PATHWAY GENOMICS®

Risk Levels

To assist you in understanding the implications of your genetic report and of your lifestyle choices, we have created five categories that summarize your risk and recommendations. For clarity, color-coded symbols representing your genetic and lifestyle risk are at the beginning of each health condition. Your genetic score is derived from a proprietary algorithm that correlates your genetic profile with published scientific research. Your lifestyle score is derived from your responses to our health survey as well as your reported personal factors of age, gender and ethnicity.

Immediate Attention (Lifestyle Only)
Your health survey responses indicated that you have lifestyle or personal factors which put you at significant risk of developing this condition. There are many factors affecting your overall risk, but we encourage you to discuss these conditions with your doctor to determine what preventive actions you can take to reduce your risk.

Take Action
Genetic: You have genetic markers that are highly correlated with these conditions. Lifestyle: Your lifestyle choices and/or your age, gender and ethnicity have indicated factors that are associated with a significant increase in risk of developing these conditions and possible opportunities for improving your health. These are many factors affecting your overall risk, and we encourage you to discuss these conditions with your doctor to determine what preventive actions you can take.

Be Proactive
Genetic: Your genetic profile shows slightly increased susceptibility for these health conditions. Lifestyle: Your responses to the health survey and/or your age, gender and ethnicity showed some factors associated with increased risk of developing these conditions and possible opportunities for improving your health. It would be appropriate to discuss these conditions with your doctor to determine what preventive actions you can take.

Learn More
Genetic: Your genetic profile did not indicate that you are at a significantly higher or lower risk for getting these conditions; most people fall into this category. Lifestyle: Your health survey did not raise any flags, but we still encourage you to learn more about these conditions and find out if there are any additional preventive actions that you can take.

Live a Healthy Lifestyle
Genetic: Your genetics do not show a strong susceptibility for these conditions. Lifestyle: You are generally making smart choices that may lower your overall risk for these conditions. As with all health conditions, you should strive to continually make healthy lifestyle choices.

HEALTH CONDITION

PATHWAY GENOMICS®

Diabetes, type 2

Genetics: **Live a Healthy Lifestyle**

Lifestyle: **Immediate Attention**

Population Risk: **33.9** /100
Will get this disease within their lifetime

These results are based on your reported ethnicity of: Caucasian

What We Tested and Your Results

Gene/Locus	SNP	Your Genotype	Odds Ratio	Associated Allele	Population Frequency	Validated Marker	PMID
CDKAL1	rs10946398	A/A	1.00	C	34%	Validated	17463249
CDKN2B	rs10811661	C/T	1.20	T	80%	Validated	17463246
HHEX	rs1111875	A/A	1.00	G	57%	Validated	17463246
IGF2BP2	rs1470579	A/C	1.19	C	30%	Validated	17463246
KCNJ11	rs5219	C/C	1.00	T	36%	Validated	17463246
KCNQ1	rs2237892	C/C	1.66	C	93%	Validated	18711367
MTNR1B	rs10830963	G/G	1.19	G	30%	Validated	19060907
PPARG	rs1801282	C/G	1.23	C	90%	Validated	17463249
SLC30A8	rs13266634	T/T	1.00	C	76%	Validated	17463249
TCF7L2	rs7903146	C/C	1.00	T	28%	Validated	17463246

What Should I Do?

Your genetic profile does not show susceptibility for type 2 diabetes. Other factors, such as lifestyle choices, may play a larger role in whether or not you get type 2 diabetes. Stay healthy with a low-fat diet, plenty of exercise, and routine visits to your doctor.

Dan's Genetic Data

Information for **Dan** assuming European ethnicity and an age range of 20–79

Dan
17.8 out of 100
men of European ethnicity who share Dan's genotype will develop Type 2 Diabetes between the ages of 20 and 79.

Average
25.7 out of 100
men of European ethnicity will develop Type 2 Diabetes between the ages of 20 and 79.

What does the Odds Calculator show me?
Use the ethnicity and age range selectors above to see the estimated incidence of Type 2 Diabetes due to genetics for men with **Dan's** genotype. The 23andMe Odds Calculator assumes that a person is free of the condition at the lower age in the range. You can use the name selector above to see the estimated incidence of Type 2 Diabetes for the genotypes of other people in your account.

The 23andMe Odds Calculator only takes into account effects of markers with known associations that are also on our genotyping chip. Keep in mind that aside from genetics, environment and lifestyle may also contribute to one's chances of developing type 2 diabetes.

Genes vs. Environment

26%
Attributable to Genetics

The heritability of type 2 diabetes is estimated to be 26%. This means that environmental factors contribute more to differences in risk for this condition than genetic factors. Genetic factors that play a role in type 2 diabetes include both unknown factors and known factors such as the SNPs we describe here. Environmental factors include obesity, gestational diabetes, giving birth to at least one baby weighing nine pounds or more, high blood pressure, abnormal cholesterol levels, physical inactivity, polycystic ovarian syndrome, other clinical conditions associated with insulin resistance, a history of impaired glucose tolerance or impaired fasting glucose, and a history of cardiovascular disease.

{DESIGN: CHECK ALL THAT APPLY}
{PRG: Q2c_d is MUTUALLY EXCLUSIVE}

Q2c. Based on what you know about Dan, what should he do next?
(*Please select all that apply.)*

Q2c_a. Discuss these results with a physician
Q2c_b. Make an appointment to get his blood sugar checked
Q2c_c. Improve his diet and exercise habits
Q2c_d. No actions are necessary based on Dan's results

{NOTE: Carrier Screening Scenarios}

Now imagine your friend Erin received the following carrier screening results:

CARRIER STATUS

PATHWAY GENOMICS®

Condition Name	Mutations Detected	No Mutations Detected
Gaucher disease		✓
Glutaric acidemia, type 1		✓
Glycogen storage disease, type 1A		✓
GM1-gangliosidosis		✓
Hearing loss, DFNB1 and DFNB9 nonsyndromic		✓
Hearing loss, DFNB59 nonsyndromic		✓
Hemochromatosis		✓
Hemoglobin C		✓
Hemoglobin E		✓
HMG-CoA lyase deficiency		✓
Homocystinuria, cblE		✓
Homocystinuria, classic		✓
Hurler syndrome		✓
Krabbe disease		✓
Lipoprotein lipase deficiency, familial		✓
Maple syrup urine disease		✓
Medium-chain acyl-CoA dehydrogenase deficiency		✓
Methylmalonic acidemia		✓
MTHFR deficiency	✓	
Mucolipidosis II		✓
Mucolipidosis III		✓
Mucolipidosis IV		✓
Multiple carboxylase deficiency		✓
Nephrotic syndrome, steroid-resistant		✓
Niemann-Pick disease		✓
Phenylketonuria	✓	
Polycystic kidney disease		✓
Pompe disease		✓
Prekallikrein deficiency		✓
Propionic acidemia		✓
Prothrombin deficiency		✓

What it means to be a carrier

Some diseases have a recessive inheritance pattern, meaning that in order to develop the disease a person must have two disease-causing mutations (also called alleles, or variants), one on each copy of the gene involved in the condition. Since we inherit two copies of each gene (one from each of our parents), usually a recessive disease occurs when a person inherits one disease-causing variant from each parent.

In a very small percentage of cases, a person inherits a disease-causing allele from one parent and has a spontaneous mutation in the normal copy of the gene inherited from the other parent, thus giving rise to two disease-causing alleles.

A person who has only one disease-causing allele is a carrier, but does not develop the disease. Carriers can pass the disease-causing variant on to their children, who will also be carriers if they inherit a variant from one parent.

If both parents are carriers, then each child from the couple has a 25% chance of inheriting two copies of the disease-causing variant and developing the disease, a 25% chance of inheriting no disease alleles and being free of the condition, and a 50% chance of being a carrier. If only one parent is a carrier, then each child has a 50% chance of inheriting one copy of the variant and being carriers themselves.

People affected with the disease will pass on one of their disease-causing alleles to each child.

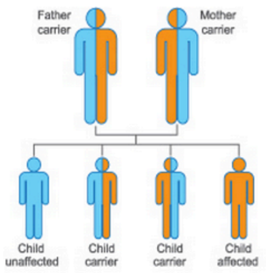

carrier status

Show results for Erin

Name	Confidence	Status ▼
Alpha-1 Antitrypsin Deficiency	★★★★	Variant Present
Phenylketonuria	★★★★	Variant Present
Bloom's Syndrome	★★★★	Variant Absent
Canavan Disease	★★★★	Variant Absent
Cystic Fibrosis	★★★★	Variant Absent
Familial Dysautonomia	★★★★	Variant Absent
Factor XI Deficiency	★★★★	Variant Absent
Fanconi Anemia (FANCC-related)	★★★★	Variant Absent
Familial Hypercholesterolemia Type B	★★★★	Variant Absent
Familial Mediterranean Fever	★★★★	Variant Absent
G6PD Deficiency	★★★★	Variant Absent
Gaucher Disease	★★★★	Variant Absent
Glycogen Storage Disease Type 1a	★★★★	Variant Absent
Hemochromatosis	★★★★	Variant Absent
Limb-girdle Muscular Dystrophy	★★★★	Variant Absent
Maple Syrup Urine Disease Type 1B	★★★★	Variant Absent
Mucolipidosis IV	★★★★	Variant Absent
Niemann-Pick Disease Type A	★★★★	Variant Absent
Connexin 26-Related Sensorineural Hearing Loss	★★★★	Variant Absent
Rhizomelic Chondrodysplasia Punctata Type 1 (RCDP1)	★★★★	Variant Absent
Sickle Cell Anemia & Malaria Resistance	★★★★	Variant Absent
Tay-Sachs Disease	★★★★	Variant Absent

close

Research Confidence

★★★★ **Established Research**. At least two studies examined more than 750 people with the trait or condition and/or the associations are widely accepted in the scientific community. The reports may cover rare conditions or include variants that do not greatly influence a person's absolute lifetime risk for a condition.

★★★ **Preliminary Research**. More than 750 people with the condition were studied, but the findings still need to be confirmed by the scientific community in an independent study of similar size.

★★ **Preliminary Research**. Fewer than 750 people were studied. Multiple large studies are needed to confirm these findings.

★ **Preliminary Research**. Fewer than 100 people were studied. Multiple large studies are needed to confirm these findings.

{DESIGN: GRID Q3a_a-Q3a_d}

Q3a. Based on these results, what would you say about Erin?

1 True

2 False

Q3a_a. Erin does not carry any variants/mutations for the diseases listed in the report

Q3a_c. Erin herself likely has one of the diseases or conditions listed in the report

Q3a_d. Erin's children could inherit a variant or mutation for one of the conditions listed in the report

{SHOW PKU SCENARIO (Q3b-Q3c IF PRE_RANDOM2=1}

Imagine your friend Erin received the following phenylketonuria (PKU) result:

CARRIER STATUS

PATHWAY GENOMICS®

Phenylketonuria

Your Results
We scanned your DNA for 14 variants related to phenylketonuria. Your DNA gave positive results for 1:

R158Q in PAH

This means that you are a carrier for this condition, but you are not likely to develop the disease yourself. To find out more about carrier status and what this means for your children, please contact our genetic counselors.

Residual risk: Since there are many rare mutations, it is possible to carry a mutation that is not on our test in addition to the variant that we found in your DNA. If you have a family history or are concerned about your status for this disease and wish to find out more, please contact our genetic counselors.

Pathway Genomics has scanned your DNA for markers related to phenylketonuria and found that you carry the following:

R158Q	Present

About the Gene

Mutations that cause PKU are found in the PAH gene, which encodes an enzyme called phenylalanine hydroxylase. The enzyme converts phenylalanine to another amino acid called tyrosine. This reaction is a critical step in removing excess phenylalanine.

Disease Description

Phenylketonuria (PKU) is the most common hereditary disorder of amino acid metabolism, inherited in an autosomal recessive manner. It is diagnosed when a harmful level of an amino acid called phenylalanine is measured in the blood. The disease is named for the finding of increased levels of phenylketone that is converted from accumulated phenylalanine and detected in the urine. Amino acids are building blocks of proteins and phenylalanine is one of the essential amino acids for humans that can only be acquired from the diet. However, excessive phenylalanine is toxic to the brain and impairs cognitive development.
Classical PKU is the most severe form of the disease. Untreated newborns with classical PKU can develop irreversible mental retardation, psychiatric problems and seizures. Symptoms are usually noticed several months after birth. Milder forms of the disease, including non-PKU hyperphenylalaninemia and variant PKU, also present high blood levels of phenylalanine, but they have lower risks for neuropsychiatric disorders.
The implementation of newborn screening programs for PKU has significantly reduced the damage of the disease. PKU can be successfully diagnosed during newborn screening and treated by restricting phenylalanine intake. However, haphazard compliance of dietary restriction, especially during childhood, can still result in cognitive impairment.

About Phenylketonuria

Phenylketonuria (PKU) is caused by mutations in the phenylalanine hydroxylase (PAH) gene. These mutations impair the PAH enzyme and prevent the body from properly metabolizing phenylalanine, leading to a toxic build up of the amino acid. Although phenylalanine is an essential protein building block, abnormally high levels lead to severe and irreversible brain damage. PKU is inherited in a recessive manner, meaning that only a child who receives two mutated copies of the PAH gene (one from each parent) will get the disease. The severity of the condition, and what measures are needed to manage it, depend on the specific mutations inherited. Thanks to widespread newborn screening, almost every child born with PKU in developed nations is identified very early on. A low-phenylalanine diet, if initiated early in life, is effective in preventing the serious disability seen in classical PKU.

Erin's Genetic Data

Who	What It Means
	Has two mutations in the PAH gene linked to severe PKU. A person with two of these mutations typically has severe PKU.
	Has two or more mutations in the PAH gene, one of which is linked to mild PKU. A person with these mutations typically has mild PKU, but may have severe PKU due to other mutations in the PAH gene (not reported here).
Erin	Has one mutation in the PAH gene linked to PKU. A person with one of these mutations typically does not have PKU, but can pass the mutation to offspring. May have other mutations in the PAH gene (not reported here).
	Does not have any of the PAH mutations reported by 23andMe. May still have other mutations in the PAH gene (not reported here).

Genes vs. Environment

PKU is inherited in a recessive manner, meaning that only a child who receives two mutated copies of the PAH gene (one from each parent) will get the disease. Many mutations in the PAH gene have been documented, and the severity of PKU depends on the particular combination of mutations a person inherits, along with other genetic and non-genetic factors. A strict low-protein diet with limited phenylalanine intake is used to prevent the developmental delay and cognitive defects caused by PAH deficiency. You may still have a PAH mutation even if your data indicates that you are a non-carrier. If you are concerned about PKU, consult a health professional.

Phenylketonuria and Your Genes

Phenylketonuria (PKU) is caused by mutations in the phenylalanine hydroxylase (PAH) gene. These mutations impair the PAH enzyme and prevent the body from properly metabolizing phenylalanine, leading to a toxic build up of the amino acid. Although phenylalanine is an essential protein building block, abnormally high levels lead to severe and irreversible brain damage. A low-phenylalanine diet, if initiated early in life, is effective in preventing the serious disability seen in classical PKU.

A person must inherit a mutated copy of PAH from each parent in order to have PKU. If two parents are carriers of a mutation, there is a 25% chance their child will be born with the disorder. There is a 50% chance that their child will be an unaffected carrier for PKU. Each unaffected sibling of an affected child has a two in three chance of being a carrier.

{PRG: SAME SCREEN Q3b-Q3d}

Q3b. Based on these results, what are the chances that Erin has phenylketonuria (PKU)?

1 Definitely has PKU
2 Most likely has PKU
3 Most likely does NOT have PKU
4 Definitely does NOT have PKU

Q3c. The father of Erin's child is a carrier of a PKU mutation. Based on these results, what is the chance for Erin's child to have PKU?

1 100% chance that Erin's child will have PKU
2 50% chance that Erin's child will have PKU
3 25% chance that Erin's child will have PKU
4 0% chance that Erin's child will have PKU

{SHOW CF SCENARIO (Q3e-Q3f IF PRE_RANDOM2=2}

Now imagine your friend Erin received the following cystic fibrosis (CF) result:

CARRIER STATUS

PATHWAY GENOMICS®

Condition Name	Mutations Detected	No Mutations Detected
3-Methylcrotonyl-CoA carboxylase deficiency		✓
Acrodermatitis enteropathica		✓
Alpha-1 antitrypsin deficiency		✓
Amyotrophic lateral sclerosis		✓
Argininosuccinate lyase deficiency		✓
Autoimmune polyglandular syndrome, type I		✓
Bartter syndrome type 4A		✓
Beta-ketothiolase deficiency		✓
Beta-thalassemia		✓
Biotinidase deficiency		✓
Bloom syndrome		✓
Canavan disease		✓
Carnitine deficiency, primary systemic		✓
Cerebrotendinous xanthomatosis		✓
Citrullinemia type I		✓
Corticosterone methyl oxidase deficiency		✓
Crigler-Najjar syndrome		✓
Cystic fibrosis		✓
Diabetes, permanent neonatal		✓
Dihydropyrimidine dehydrogenase deficiency		✓
Dubin-Johnson syndrome		✓
Ehlers-Danlos syndrome, dermatosparaxis		✓
Ehlers-Danlos syndrome, hypermobility		✓
Ehlers-Danlos syndrome, kyphoscoliotic		✓
Ethylmalonic aciduria		✓
Factor XI deficiency		✓
Familial dysautonomia		✓
Familial Mediterranean fever		✓
Fanconi anemia		✓
Galactokinase deficiency		✓
Galactosemia		✓

Residual risk: Since there are many rare mutations, it is possible to carry a mutation that is not on our test. If you have a family history or are concerned about your status for this disease and wish to find out more, please contact your physician or a genetic counselor.

About Cystic Fibrosis

Cystic fibrosis (CF) is a serious disease caused by mutations in the Cystic Fibrosis Transmembrane Conductance Regulator (CFTR) gene that affect the ability of cells lining the inner surfaces of organs to properly transport salt and water. Most significantly, these mutations can cause mucus to build up in the lungs, which leads to infections and damage. There are also effects on the pancreas and reproductive system. A person must inherit a mutated copy of the gene from each parent to develop the disease. Approximately 30,000 children and adults in the United States (70,000 worldwide) are living with CF and an estimated eight million people carry one copy of a CFTR mutation.

Erin's Genetic Data

Who	What It Means
	Has two copies of the DeltaF508 mutation in the CFTR gene linked to cystic fibrosis. A person with two DeltaF508 mutations typically has cystic fibrosis.
	Has two mutations in the CFTR gene linked to cystic fibrosis. A person with two of these mutations typically has cystic fibrosis, but severity of symptoms may vary.
	Has one mutation in the CFTR gene linked to cystic fibrosis. A person with one of these mutations typically does not have cystic fibrosis, but may pass the mutation to offspring. May still have other mutations in the CFTR gene (not reported here).
Erin	Does not have any of the 31 CFTR mutations reported by 23andMe. May still have other mutations in the CFTR gene (not reported here).

Genes vs. Environment

Cystic fibrosis is inherited in a recessive manner, meaning that only a child who receives two mutated copies of the CFTR gene (one from each parent) will get the disease. More than 500 known mutations of the CFTR gene have been documented to cause CF, though most are extremely rare. In this report, 23andMe determines the presence or absence of 31 of the most common mutations. This means you may still have a CFTR mutation or be affected by cystic fibrosis even if your data indicates you are a non-carrier. If you are concerned about cystic fibrosis, consult a health professional.

{PRG: SAME SCREEN Q3e-Q3g}

Q3e. Based on these results, what are the chances that Erin has cystic fibrosis (CF)?

1 Definitely has cystic fibrosis
2 Most likely has cystic fibrosis
3 Most likely does NOT have cystic fibrosis
4 Definitely does NOT have cystic fibrosis

Q3f. Based on these results, what is the chance that Erin is a carrier of a CF mutation?

1 Definitely is a carrier
2 Most likely is a carrier
3 Most likely is NOT a carrier
4 Definitely is NOT a carrier

{NOTE: Drug Response Scenarios}

Now for the last scenario…

Imagine your friend Frank takes a statin drug called simvastatin to reduce his cholesterol level. Frank receives the following drug response results:

DRUG RESPONSE

PATHWAY GENOMICS®

Statins : Myopathy

Results

Patient has a genetic marker that significantly increases the risk of statin-induced myopathy (muscular pain and damage). About 5-10% of patients taking statins experience myopathy. The potential risk of myopathy should be weighed against the benefits of statins. If the patient is being treated with a statin, muscle symptoms should be carefully monitored. A reduced statin dose or non-statin therapeutic could be considered.

About this medication

Statins (atorvastatin, fluvastatin, lovastatin, pitavastatin, pravastatin, rosuvastatin, simvastatin) are a type of widely prescribed cholesterol-lowering medicines. They block the production of cholesterol in cells by inhibiting a certain enzyme that is critical in the synthesis of cholesterol.

We evaluated the following markers

Gene	Marker	Your Genotype
SLCO1B1	rs4149056	T/C

Genetics of this response

The major adverse effect of statins is pain and damage in the skeletal muscles (myopathy). About 5-10% of patients taking statins experience muscle pain (myalgia). A small portion of patients may develop more severe symptoms including muscle weakness, muscle cramps, myositis (inflammation of muscles, may be accompanied by increased creatine kinase levels in the blood), and the rare but potentially lethal rhabdomyolysis. In rare cases, myalgia and creatine kinase elevations persist after statin withdrawal. When rhabdomyolysis occurs, skeletal muscles rapidly break down, releasing large quantities of muscle cell contents into the blood. Some of those contents, such as myoglobin, cannot be properly processed by the kidneys and may lead to acute renal failure and death. In randomized, controlled trials, reported incidence of statin-induced myopathy ranges from 1.5% to 5.0%. The rate of statin-induced rhabdomyolysis is approximately 0.1 to 0.2 cases per 1000 person-years. The risk of myopathy varies with the type of statin and is dose-related. The incidence of myopathy while taking 80 mg simvastatin daily is more than 25 times the incidence of a daily dose of 20 mg.

drug response

Statin Response ★★

Preliminary Research report.

Frank's Data

About Statin Response

Statin drugs are prescribed to reduce cholesterol levels in people who have a high risk of cardiovascular disease. Though generally very safe, statins can cause some adverse effects, including liver problems, muscular soreness and an extremely rare condition known as rhabdomyolysis in which the muscles begin to disintegrate. Physicians can use blood tests to detect the drugs' most serious adverse effects.

Statin-related myopathy

Journal	*N Engl J Med*
Study Size	
Replications	None
Contrary Studies	None
Applicable Ethnicities	European
Marker	rs4149056

Who	Genotype	What It Means
	CC	Greatly increased odds of myopathy while on simvastatin.
Frank	CT	Substantially increased odds of myopathy while on simvastatin.
	TT	Typical odds of myopathy while on simvastatin.

About one person in 10,000 who takes statin drugs experiences muscle pain and/or weakness in a given year, a condition known as myopathy. It appears that higher doses increase the condition's likelihood. This study compared 85 patients who experienced myopathy to 90 patients who did not. All were taking 80 mg of simvastatin daily — a relatively high dose — as part of a much larger study involving 12,000 patients who were taking the drug after experiencing a heart attack. The authors found that having one C at rs4149056, a SNP in the SLCO1B1 gene, increased a person's odds of having myopathy 4.5 times compared to the TT genotype. Having two C copies of the SNP increased a person's odds of myopathy by about 17 times. The authors of the study succeeded in detecting a similar effect in individuals taking 40 mg of simvastatin daily, but the effect was much less extreme. Please note that myopathy is a very rare side effect of statins even among those with genotypes that increase their odds of experiencing it. Anyone who is following a statin regimen and has concerns about the risk of myopathy should consult a physician before taking any action.

Citations

SEARCH Collaborative Group et al. (2008). "SLCO1B1 variants and statin-induced myopathy--a genomewide study." *N Engl J Med* 359(8):789-99.

{PRG: SAME SCREEN Q4a-Q4c_d}

Q4a. Based on his statins drug response results, what are Frank's chances of myopathy while taking statin therapy?

1 Higher than average
2 About average
3 Lower than average

{DESIGN: CHECK ALL THAT APPLY}
{PRG: Q4c_d IS MUTUALLY EXCLUSIVE}

Q4c. Based on these results, what should Frank do next?
(*Please select all that apply.)*

Q4c_a. Frank should make an appointment with his physician to discuss these results
Q4c_b. Frank should stop taking simvastatin immediately
Q4c_c. Frank should request different medications from his doctor to lower his cholesterol level
Q4c_d. No actions are necessary based on Frank's results

{NOTE: SECTION HEADER “Utility of Genetic Results”}

INTRO_4: Section 4 of 4: Utility of Genetic Results

P1. How many times have you viewed your {DISPLAY PRE_1} results since receiving them?

1 1 time
2 2-3 times
3 4 or more times

{SHOWP2 IF P1 DOES NOT = 0}

P2. In total, how much time have you spent reviewing the {DISPLAY PRE_1} web site since receiving your {DISPLAY PRE_1} results?
1 Less than 30 minutes
2 30-60 minutes
3 1-2 hours
4 More than 2 hours

{DESIGN: GRID P5-P8}

To what extent do you agree or disagree with the following statements?

1 Strongly Disagree
2 Somewhat Disagree
3 Neither Disagree nor Agree
4 Somewhat Agree
5 Strongly Agree

P6. The information I received from {DISPLAY PRE_1} has influenced how I will manage my health in the future
P7. Having personal genomic testing made me feel like I have more control over my health
P8. Having personal genomic testing helped me to get a better perspective on my health status
P9. What I learned from my personal genomic testing can help reduce my chances of getting sick

---

P3_TEXT. **Please read the following statements and select the response that best applies to you.**

{DESIGN: GRID P3-P4}

1 Not at all
2 A little
3 Somewhat
4 Very
5 Extremely

P3. In general, how satisfied are you regarding your decision to obtain personal genomic testing?
P4. In general, how valuable were your {DISPLAY:PRE_1} results?

{NO soft prompt on P4b}

P4b. Please explain why you think the personal genomic testing experience was valuable or was not valuable.

[OPEN END RESPONSE]
