# Supplementary material for: Design, methods, and participant characteristics of the Impact of Personal Genomics (PGen) Study, a prospective cohort study of direct-to-consumer personal genomic testing customers
Source: Genome Med. 2014 Dec 3;6(12):96. doi: 10.1186/s13073-014-0096-0 (PMC4256737; doi:10.1186/s13073-014-0096-0)
Supplement: Additional file 3: — 6 month follow-up PGen Study survey. [file 13073_2014_96_MOESM3_ESM.pdf]

6 Month Follow Up Questionnaire Specifications for PGen/ S10028 (F9)

Short URL to direct towards production survey	https://www.ssgresearch.com/pgen3
Support email address to include in header	pgen@ssgresearch.com
Support phone number to include in header (if needed)	

Logo to use if other than SSG logo
Please list network location of other logo to use:

Mandatoriness (check the appropriate setting)

	All questions are optional unless otherwise noted
x	All questions are optional with a soft prompt included if no answer is provided [OTHER SPECIFY FIELDS SHOULD ALSO HAVE SOFT PROMPTS]
	All questions are mandatory

Please provide text to use for Mandatoriness prompt if being used (Default text to use is provided below):
General:

We noticed that you did not answer a question on the previous page. It is important to us that we get a complete set of responses from you. Please return to the previous page by clicking "Previous" and select an answer for each question. If you would rather not select an answer, you may instead continue to the next page by clicking "Next."

Other specify:

You selected 'Other' but did not specify your answer. Please return to the last question by clicking "Previous" and type in your specific answer. If you would rather not specify an answer, you may instead continue to the next page by clicking "Next."

Header Sections (if being used)

Section Label	Questions in Section	Section Label	Questions in Section
Q1-B3	Using Your Genetic Results	G1- M3b_1	Health, Behaviors and Insurance
C1-C10	Screening	M4, S1-S4	Genetics and Sharing Your Results
D1	How You're Feeling		
D2-D3	Reactions to Genetic Results		
E1-E2h	Risk Perceptions		
F1-F8	Utility of Genetic Results		

Survey Title to appear in header (appears above the section header bar)

The Impact of Personal Genomics (PGen) Study

Welcome Page text (please modify the following as needed)

Welcome to the Impact of Personal Genomics (PGen) Study!
This is the last of three surveys for this study.

Resume Page text (please modify the following as needed)

Thank you for returning to the survey. Please click "resume" to begin where you last left off…

End Page Text

Thank you for your participation! This concludes the last survey for this research study.

For information about the PGen Study, please refer to the study website at:

http://www.genomes2people.org/g2p/pgen/

Results of the PGen Study will be posted on the study website as they become available.

Within the next 2 weeks, you will receive a $20 Amazon.com gift certificate via email.

You may now close your browser.

Survey Title appearing in browser window

PGen Study

GENERAL PROGRAMMING NOTES

All emphasis should be programmed in black, all caps text instead of lowercase blue text.

Preloads

PRE_1 Genetics Company
- 1 Pathway Genomics
- 2 23andMe

{Note: Will preload PRE_2 from DEM3 in baseline survey}
PRE_2 Gender
- 1 Male
- 2 Female

PRE_C2. Conditions of interest from baseline survey
{Note: Will preload variables below based on selections from baseline survey}
C2_1 Osteoarthritis
C2_2 Rheumatoid arthritis
C2_3 Asthma
C2_6 Celiac disease
C2_8 Ulcerative colitis
C2_9 Breast cancer (females only)

C2_10 Colorectal cancer
C2_12 Leukemia
C2_13 Lung cancer
C2_14 Prostate cancer (males only)
C2_15 Skin cancer (Melanoma)
C2_17 Heart disease (Coronary artery disease)
C2_20 Blood clotting (Venous thromboembolism)
C2_21 Chronic kidney disease
C2_22 High cholesterol
C2_23 Diabetes
C2_24 Age-related macular degeneration
C2_25 Glaucoma
C2_26 Bipolar disorder
C2_27 Alzheimer's disease
C2_28 ALS (Lou Gehrig's disease)
C2_29 Multiple sclerosis
C2_30 Parkinson's disease
C2_31 Obesity

{PRG NOTE: Section Header: Using Your Genetic Results}

{GRID Q1a-Q1i}

Q1_TEXT. Please read the following statements and select the response that best applies to you.

Q1. The personal genomic information I received from {DISPLAY PRE_1} allowed me…

1 Not at all
2 Somewhat
3 Very Much

Q1a. To satisfy my curiosity about my genetic makeup.
Q1b. To see if I was at risk for specific diseases.
Q1c. To learn about my genes without going through a physician.
Q1d. To find out how I can improve my health.
Q1e. To find out about my individual response to different types of medications.
Q1f. To better plan for the future.
Q1g. To participate in something fun and entertaining.
Q1h. To satisfy my interest in genetics in general.
Q1i. To participate in research.

{GRID Q1j-Q1l}

Q1_TEXT. Please read the following statements and select the response that best applies to you.

Q1. The personal genomic information I received from {DISPLAY PRE_1} allowed me…

1 Not at all
2 Somewhat
3 Very Much
4 Not Applicable

Q1j. To learn more about my genetics because I have limited information about my family health history.
Q1k. To learn more about my genetics because I am adopted.
Q1l. To get information about the risk of health conditions for my current children or future children.

A. COMMUNICATION OF RESULTS AND GENETICS AND HEALTH-INFORMATION SEEKING

AD1. Did you know others who used a personal genomics service prior to you?

1 Yes
0 No

{PRG: SHOW AD1_A IF AD1 = 1 OTHERWISE GOTO AD2}

AD1_A. What is their relationship to you? *(Select all that apply)*

1 Family members
2 Friends
3 Co-workers / colleagues
4 Contacts on social networking services (e.g., Facebook, MySpace, Twitter)
5 Contacts on health- or disease-related social networking services (e.g. Patients Like Me, 23andMe's discussion forum, Cure Together, disease-specific patient networks)
6 Other *(Please specify):* [TEXT RESPONSE]

AD2. Since you received your [DISPLAY PRE_1] results, have you encouraged others to obtain personal genomic testing?

1 Yes
0 No

{PRG: SHOW AD2_A IF AD2=1 OTHERWISE GOTO AD3}

AD2_A. Who have you encouraged to obtain personal genomic testing? *(Select all that apply)*

1 Family members
2 Friends
3 Co-workers / colleagues
4 Contacts on social networking services (e.g., Facebook, MySpace, Twitter)
5 Contacts on health- or disease-related social networking services (e.g. Patients Like Me, 23andMe's discussion forum, Cure Together, disease-specific patient networks)
6 Other *(Please specify):* [TEXT RESPONSE]

AD3. To your knowledge, has anyone you know purchased personal genomic testing sine you received your results?

1 Yes
0 No

{PRG: SHOW AD3_A IF AD3 = 1 OTHERWISE GOTO A1}

AD3_A. Who do you know that has purchased personal genomic testing since you received your [DISPLAY PRE_1] results?

1 Family members
2 Friends
3 Co-workers / colleagues
4 Contacts on social networking services (e.g., Facebook, MySpace, Twitter)

5 Contacts on health- or disease-related social networking services (e.g. Patients Like Me, 23andMe's discussion forum, Cure Together, disease-specific patient networks)
6 Other *(Please specify):* [TEXT RESPONSE]

A1. Have you discussed your {DISPLAY PRE_1} results with anyone?

1 Yes
0 No

{PRG: SHOW A1_A IF A1 = 0 OTHERWISE GOTO FILTER BEFORE A2}

A1_A. Why have you **NOT** discussed your [DISPLAY PRE_1] results with anyone?

[OPEN ENDED RESPONSE]

{PRG: SHOW IF A1=1; OTHERWISE SKIP TO A3}

A2. With whom did you discuss your {DISPLAY PRE_1} results?
(Select all that apply)

1 Family members
2 Friends
3 Co-workers/ colleagues
4 Primary care provider
5 Genetics specialist (e.g. genetic counselor, clinical geneticist)
6 Other medical professional
7 Contacts on social networking services (e.g., Facebook, MySpace, Twitter)
8 Contacts on health- or disease-based social networking services (e.g. Patients Like Me, 23andMe's discussion forum, Cure Together, disease-specific patient networks)
9 Other *(Please specify):* [TEXT RESPONSE]

{PRG: SHOW A2_A IF NO VALUE OF A2=1, OTHERWISE GOTO A2_1A}

A2_A. Why have you **NOT** discussed your [DISPLAY PRE_1] results with family members? *(Select all that apply).*

1 I don't feel that my results are important enough to share.
2 I don't think family members are interested in my results.
3 I am concerned about how my family members would react to my results.
4 I plan to discuss my results with family members but haven't gotten around to it.
5 Other *(Please specify)* [TEXT RESPONSE]

{PRG: SHOW IF A2=1; OTHERWISE SKIP TO FILTER BEFORE A2_6a}
{PRG: SELECT ALL THAT APPLY}

A2_1a. With which family member(s) did you talk about your {DISPLAY PRE_1} results?

(Select all that apply)

1 Spouse/significant other
2 Children
3 Brothers or sisters
4 Parents
5 Other relatives *(Please specify)* [TEXT RESPONSE]

{PRG: SHOW A2_B IF A2 DOES NOT EQUAL 4 OR 5 OR 6 OTHERWISE GOTO FILTER BEFORE A2_6A}

A2_B. Why have you **NOT** discussed your [DISPLAY PRE_1] results with a medical professional? *(Select all that apply*)

1 I would have concerns about my [DISPLAY PRE_1] results being placed in my medical record
2 I don't feel that my results are important enough to share.
3 I plan to discuss my results with a medical professional but haven't gotten around to it.
4 Other (*Please specify)* [TEXT RESPONSE]

{PRG: SHOW IF A2=4; OTHERWISE SKIP TO FILTER BEFORE A2_5a}

A2_4B. How willing was your primary care provider to discuss the meaning of your [DISPLAY PRE_1] results?
1 Not at all willing
2 Somewhat willing
3 Very willing

A2_4C. How willing was your primary care provider to use your [DISPLAY PRE_1] results in your medical care?
1 Not at all willing
2 Somewhat willing
3 Very willing

A2_4D. Did the interpretation of your results provided by your primary care provider differ from the interpretation provided by [DISPLAY PRE_1]?
1 Yes
0 No

{PRG: SHOW A2_4D1 IF A2_4D = 1 OTHERWISE GOTO A2_4A}

A2_4D1. How much do you trust the interpretation from your primary care provider?
1 Not at all
2 A little
3 Somewhat
4 Very
5 Extremely

A2_4D2. How much do you trust the interpretation from [DISPLAY PRE_1]?

1 Not at all
2 A little
3 Somewhat
4 Very
5 Extremely

{PRG: A2_4E IS EXEMPT FROM SOFT PROMPT}
{DESIGN: SHOW A2_4A AND A2_4E ON SAME PAGE}

A2_4a. How satisfied were you with your discussion of your {DISPLAY PRE_1} results with your primary care provider?

1 Not at all satisfied
2 Somewhat satisfied
3 Very satisfied

A2_4E. Please explain why you were or were not satisfied with your discussion of your [DISPLAY PRE_1] results with your primary care provider. *(Optional)*

[OPEN ENDED RESPONSE]

{PRG: SHOW IF A2=5; OTHERWISE SKIP TO FILTER BEFORE A2_6}
{DESIGN: SHOW A2_5A AND A2_5E ON SAME PAGE}
{PRG: A2_5E IS EXEMPT FROM SOFT PROMPT}

A2_5a. How satisfied were you with your discussion of your {DISPLAY PRE_1} results with your genetic counselor/specialist?

1 Not at all satisfied
2 Somewhat satisfied
3 Very satisfied

A2_5E. Please explain why you were or were not satisfied with your discussion of your [DISPLAY PRE_1] results with your genetic counselor / specialist *(Optional)*

[OPEN ENDED RESPONSE]

A2_5b. Did you find the genetic counselor/specialist through {DISPLAY PRE_1}?

1 Yes
0 No

{PRG: SHOW IF A2_5b=0; OTHERWISE SKIP TO FILTER BEFORE A2_6}
{PRG: SELECT ALL THAT APPLY}

A2_5c. How did you find the genetic counselor/specialist?
(Select all that apply)

1 Recommendation by primary care provider
2 Recommendation by other health care provider
3 National Society of Genetic Counselors (NSGC) website
4 Internet search
5 Other *(Please specify):* [TEXT RESPONSE]

{PRG: SHOW IF A2=6; OTHERWISE SKIP TO FILTER BEFORE A2_6}

A2_6a. With what {PRG: IF A2=4 SHOW "OTHER"} type of medical professional(s) did you talk about your {DISPLAY PRE_1} results? *(Please check all that apply.)*

1 Anesthesiologist
2 Nutritionist
3 Obstetrician/Gynecologist
4 Oncologist
5 Physician assistant, nurse, or medical assistant
6 Reproductive Endocrinologist
7 Surgeon
8 Pediatrician/Child's physician
9 Other specialist/Other medical professional *(Please specify)* [TEXT RESPONSE]

A3. Do you plan to discuss your {DISPLAY PRE_1} results with anyone {PRG: IF A1 = 1 DISPLAY "else"}?

1 Yes
0 No

{PRG: SHOW A4 IF A3=1, OTHERWISE SKIP TO A5}
{PRG: A4 SELECT ALL THAT APPLY}

A4. With whom do you plan to discuss your {DISPLAY PRE_1} results?
(Select all that apply)

1 Health care provider
2 Spouse/significant other
3 Other family members
4 Friends
5 Co-workers/colleagues
6 Other *(Please specify)* [TEXT RESPONSE]

{PRG: S5 SELECT ALL THAT APPLY}

S5. If you had a question about your {DISPLAY PRE_1} results, whom would you consult?
(Select all that apply)

1 Spouse/significant other
2 Other family member
3 A friend
4 A healthcare provider

5 {DISPLAY PRE_1}
6 Health and medical websites
7 Other *(Please specify)* [TEXT RESPONSE]
8 No one

S6. How many people have you consulted with questions about your {DISPLAY PRE_1} results?

0 No one
1 1
2 <5
3 5-10
4 >10

A5. Have your {DISPLAY PRE_1} results prompted you to seek out more information about health or medical topics related to your results?

1 Yes
0 No

{PRG: SAME SCREEN A5a-A5b}
{PRG: SHOW A5a-A5b IF A5=1; OTHERWISE SKIP TO A6}
{PRG: A5A – A5B EXEMPT FROM SOFT PROMPT}

A5a. What type of information have your {DISPLAY PRE_1} results prompted you to seek out? *(Optional)*

[OPEN END RESPONSE]

A5b. Where did you get the information? ? *(Optional)*

[OPEN END RESPONSE]

A6. Have your {DISPLAY PRE_1} results prompted you to make an appointment with a medical professional(s)?

2 Yes, I have already made an appointment
1 I plan to make an appointment
0 No, I do not plan to make an appointment

{PRG: SHOW IF A6=1 or 2; OTHERWISE SKIP TO S1}

A6a. What type of medical professional(s) have your {DISPLAY PRE_1} results prompted you to make an appointment with?

10 Primary care provider
11 Genetics specialist (e.g. genetic counselor, clinical geneticist)
1 Anesthesiologist
2 Nutritionist
3 Obstetrician/Gynecologist

4 Oncologist
5 Physician assistant, nurse, or medical assistant
6 Reproductive Endocrinologist
7 Surgeon
8 Pediatrician/Child's physician
9 Other specialist/Other medical professional *(Please specify)* [TEXT RESPONSE]

A6B. What was it about your [DISPLAY PRE_1] results that prompted you to make an appointment with a medical professional(s)?

[OPEN ENDED RESPONSE]

{DEISGN: GRID A7}

Please indicate how much you agree or disagree with the following statement.

1 Strongly Disagree
2 Somewhat Disagree
3 Neither Agree nor Disagree
4 Somewhat Agree
5 Strongly Agree

A7. I believe that my primary care physician understands genetics well enough to advise me on the implications of my {DISPLAY PRE_1} results for my health.

B. HEALTH CARE UTILIZATION

B1. As a result of seeing your genetic information from {DISPLAY PRE_1}, have you SPOKEN WITH YOUR DOCTOR about any other tests, medical exams, or procedures?

1 Yes
0 No

{PRG: SHOW B1_2 IF B1 = 1 OTHERWISE SKIP TO B2}

B1_2. Who initiated these other tests, medical exams, or procedures?
1 I requested them
2 My doctor suggested them
3 They were a combination of my requests and my doctor's suggestions

---

{PRG: SHOW B1a IF B1=1 OTHERWISE SKIP TO B2}

B1a. What kind of tests, medical exams, or procedures have you spoken about with your doctor as a result of seeing your genetic information from {DISPLAY PRE_1}? *(Select all that apply)*

1	Genetic test(s) to confirm a variant such as BRCA1, BRCA2, Factor II or Factor V, or other carrier status variants, such as cystic fibrosis
2	Medical exams or procedures to screen or test for a specific disease/condition
3	Whole body scan
4	Other

{PRG: SHOW B1B IF B1A = 1}

B1B. Please specify the genetic test(s) to confirm a variant that you spoke about with your doctor as a result of seeing your genetic information.

[TEXT RESPONSE]

{PRG: SHOW B1C IF B1A = 2}

B1C. Please specify the medical exams or procedures you spoke about with your doctor as a result of seeing your genetic information.

[TEXT RESPONSE]

B2. As a result of seeing your genetic information from {DISPLAY PRE_1}, have you HAD any tests, medical exams, or procedures?

1	Yes
0	No

{PRG: SHOW B2a IF B2=1}

B2a. What kind of tests, medical exams, or procedures did you have as a result of seeing your genetic information from {DISPLAY PRE_1}? (*Please select all that apply)*

1	Genetic test(s) to confirm a variant such as BRCA1, BRCA2, Factor II or Factor V, or other carrier status variants, such as cystic fibrosis
2	Medical exams or procedures to screen or test for a specific disease/ condition
3	Whole body scan
4	Other

{PRG: SHOW B2D IF B2A = 1}

B2D. Please specify the genetic test(s) to confirm a variant that you had as a result of seeing your genetic information.

[TEXT RESPONSE]

{PRG: SHOW B2E IF B2A = 2}

B2E. Please specify the medical exams or procedures that you had as a result of seeing your genetic information.
[TEXT RESPONSE]

{PRG: SHOW B2b IF B2a=2 and/or 3; OTHERWISE SKIP TO FILTER BEFORE B2c}

B2b. Were you diagnosed with any diseases/conditions as a result of these medical exams or procedures?

1 Yes
0 No

{PRG: SHOW B2b_1 IF B2b=1; OTHERWISE SKIP TO FILTER BEFORE B2c}

B2b_1. What disease(s) or condition(s) were you diagnosed with?

[OPEN END RESPONSE]

{PRG: SHOW B2c IF B2a=1}

B2c. Were the results of your genetic test(s) to confirm a genetic variant consistent or inconsistent with your [DISPLAY PRE_1] data?

1 Consistent
2 Inconsistent
3 Results are still pending

{PRG NOTE: SECTION HEADER: Screening}

Now we would like to ask you a few questions about your routine health screening.

C1. Blood cholesterol is a fatty substance found in the blood. Blood can be taken and used to determine your cholesterol level.

Since receiving your {DISPLAY PRE_1} results, have you had a blood test to check your cholesterol?

1 Yes
0 No
99 Don’t know or not sure

C2. A blood sugar test is a blood test that measures your blood glucose or blood sugar.

Since receiving your {DISPLAY PRE_1} results, have you had your blood sugar tested?

1 Yes
0 No
99 Don’t know or not sure

C3. A colonoscopy is when a tube is inserted in the rectum to view the bowel for signs of cancer or other health problems. In this exam, the entire colon is checked. Anesthesia or pain medication is usually required.

Since receiving your {DISPLAY PRE_1} results, have you had a colonoscopy?

1	Yes
0	No
99	Don't know or not sure

C4. Since receiving your {DISPLAY PRE_1} results, have you had any tests in which a physician or healthcare professional looked for signs of heart disease?

1	Yes
0	No
99	Don't know or not sure

{PRG: SHOW C5-C6 IF PRE_2=2; OTHERWISE SKIP TO FILTER BEFORE C7}

C5. A mammogram is an x-ray of each breast to look for early signs of breast cancer.

Since receiving your {DISPLAY PRE_1} results, have you had a mammogram?

1	Yes
0	No
99	Don't know or not sure

C5a. MRI scans use magnets and radio waves instead of x-rays to produce very detailed, cross-sectional images of the body. MRI scans can take a long time -- often up to an hour. You have to lie inside a narrow tube. For breast imaging, doctors inject a dye into a small vein in the arm before or during the exam.

Since receiving your {DISPLAY PRE_1} results, have you had a breast MRI scan?

1	Yes
0	No
99	Don't know or not sure

C6. A clinical breast exam is when a physician, nurse, or other health professional feels your breasts for lumps.

Since receiving your {DISPLAY PRE_1} results, have you had a clinical breast exam?

1	Yes
0	No
99	Don't know or not sure

{PRG: SHOW IF DEM3=2; OTHERWISE SKIP TO C8}

C7. A Pap test, also called a Pap smear, is when a physician or other healthcare professional uses a special stick or brush to take a few cells from inside and around the cervix.

Since receiving your {DISPLAY PRE_1} results, have you had a Pap test?

1 Yes
0 No
99 Don't know or not sure

{PRG: SHOW IF PRE_2=1; OTHERWISE SKIP TO C8}

C8. A Prostate-Specific Antigen test, also called a PSA test, is a blood test used to check men for prostate cancer.

Since receiving your {DISPLAY PRE_1} results, have you had a PSA test?

1 Yes
0 No
99 Don't know or not sure

C9. Since receiving your {DISPLAY PRE_1} results, have you had any additional blood tests, imaging studies, or exams in which a physician or healthcare professional looked for signs of cancer?

1 Yes
0 No
99 Don't know or not sure

{PRG: C9A IS CHECK ALL THAT APPLY}
{PRG: SHOW C9A IF C9 = 1 OTHERWISE GOTO C10}

C9A. What additional cancer screening tests / exams have you had since receiving your [DISPLAY PRE_1] results?
1 Skin cancer (melanoma) screening
2 Lung cancer screening
3 Other *(Please specify)* [TEXT RESPONSE]

C10. In what calendar year did you have your last physical check-up?

[NUMERIC RANGE 1950-2012]

C11. In the past 6 months, how many medical visits with a physician or healthcare provider have you had?

[NUMERIC RANGE 0-50] visits

{PRG NOTE: SECTION HEADER: "How You're Feeling"}

{DESIGN: GRID D1a-B1b}

D1. Over the **past two weeks**, how often have you:

1 Not at all
2 Several days
3 More than half of the days
4 Nearly every day

D1a. Felt nervous, anxious, or on edge?
D1b. Been unable to stop or control worrying?
D1c. Felt calm and peaceful?
D1d. Been a happy person?
D1e. Had little interest or pleasure in doing things?
D1f. Felt down, depressed or hopeless?

{PRG NOTE: SECTION HEADER: Reactions to Genetic Results}
{PRG: D3B IS EXEMPT FROM SOFT PROMPT}

D2_TEXT. **Please read the following statements and select the response that best applies to you.**

{DESIGN: GRID D2a-D2k}

D2. Since receiving my genetic results from {DISPLAY PRE_1}, I have…

1 Never
2 Rarely
3 Sometimes
4 Often

Felt surprised about my results
Felt disappointed about my results
D2a. Felt upset about my results
D2b. Felt relieved about my results
D2c. Felt happy about my results
D2d. Felt motivated to change my lifestyle because of my results
D2e. Worried about my risk of getting diseases
D2f. Been uncertain about what my results mean about my risk of developing diseases
D2g. Been uncertain about what my results mean for my child(ren)'s and/or family's disease risk
D2h. Felt unsure about what to do to prevent diseases
D2i. Felt concerned about how my results will affect my insurance status
D2j. Had difficulty talking about my results with others
D2k. Wanted to tell others about my results

{PRG: NO SOFT PROMPT FOR D3}
D3. Which result or results were most important to you?

[OPEN END RESPONSE]

D3b. Please explain why this result or results were most important to you.

[OPEN END RESPONSE]

{PRG NOTE: SECTION HEADER: "Risk Perceptions"}

{DESIGN: GRID E1_1-E_12}

E1. Compared to the average [DISPLAY "man" IF PRE_2=1 OR "woman" IF PRE_2=2] of your age, what would you say your chances are of developing these conditions?

1 Much lower than average
2 Lower than average
3 Average
4 Higher than average
5 Much higher than average
6 I already have this condition

E1_1. Alzheimer's disease
{PRG: SHOW E1_2 IF PRE_2=2}
E1_2. Breast cancer
{PRG: SHOWE1_3 IF PRE_2=1}
E1_3. Prostate cancer
E1_4. Colorectal cancer
E1_5. Lung cancer
E1_6. Diabetes
E1_7. Heart disease (Coronary artery disease)
E1_8. Obesity
E1_9. Parkinson's disease

{PRG: SHOW E1_C2_1 IF BASELINE C2.1=3}
E1_C2_1 Osteoarthritis
{PRG: SHOW E1_C2_2 IF BASELINE C2.2=3}
E1_C2_2 Rheumatoid arthritis
{PRG: SHOW E1_C2_3 IF BASELINE C2.3=3}
E1_C2_3 Asthma
{PRG: SHOW E1_C2_6 IF BASELINE C2.6=3}
E1_C2_6 Celiac disease
{PRG: SHOW E1_C2_8 IF BASELINE C2.8=3}
E1_C2_8 Ulcerative colitis
{PRG: SHOW E1_C2_12 IF BASELINE C2.12=3}
E1_C2_12 Leukemia
{PRG: SHOW E1_C2_15 IF BASELINE C2.15=3}
E1_C2_15 Skin cancer (Melanoma)
{PRG: SHOW E1_C2_20 IF BASELINE C2.20=3}
E1_C2_20 Blood clotting (Venous thromboembolism)
{PRG: SHOW E1_C2_21 IF BASELINE C2.21=3}
E1_C2_21 Chronic kidney disease
{PRG: SHOW E1_C2_22 IF BASELINE C2.22=3}
E1_C2_22 High cholesterol

{PRG: SHOW E1_C2_24 IF BASELINE C2.24=3}
E1_C2_24 Age-related macular degeneration
{PRG: SHOW E1_C2_25 IF BASELINE C2.25=3}
E1_C2_25 Glaucoma
{PRG: SHOW E1_C2_26 IF BASELINE C2.26=3}
E1_C2_26 Bipolar disorder
{PRG: SHOW E1_C2_28 IF BASELINE C2.28=3}
E1_C2_28 ALS (Lou Gehrig's disease)
{PRG: SHOW E1_C2_29 IF BASELINE C2.29=3}
E1_C2_29 Multiple sclerosis

{DESIGN: GRID E2a-E2h}

E2. Out of all the genetic results you received from {DISPLAY PRE_1}, how many were of the following types?

0 None of them
1 A few of them
2 Many of them
3 All of them
99 Don't know or not sure

E2a. Results you found interesting
E2b. Results showing you are at higher than average risk for a certain condition
E2c. Results showing you are at lower than average risk for a certain condition
E2d. Results that show you have average or near average risks for a certain condition
E2e Results showing that you are a carrier for a genetic condition/disease
E2f. Results that show you may respond differently to certain medications
E2g. Results you can use to improve your health
E2h. Results you don't understand

{PRG NOTE: SECTION HEADER: "Utility of Genetic Results"}

F1. How many times have you viewed your {DISPLAY PRE_1} results since receiving them?

1 1 time
2 2-3 times
3 4 or more times

{PRG: SHOW F1A IF F1 = 2 OR 3}
{PRG: F1A IS EXEMPT FROM SOFT PROMPT}

F1A. Why have you viewed your results more than once? *(Optional)*
[OPEN ENDED RESPONSE]

{SHOW F2 IF F1 > 0}

F2. In total, how much time have you spent reviewing your {DISPLAY PRE_1} results?

1 Less than 1 hour
2 1-2 hours
3 2-5 hours
4 5-10 hours
5 More than 10 hours

{DESIGN: GRID F2_A – F2_F}
{PRG: SHOW F2_E – F2_F IF PRE_1 = 2 OTHERWISE HIDE}

Now that you have received your results, to what extent are you interested in the following types of information?
1 Not at all interested
2 Somewhat interested
3 Very interested

F2_A. Risk of disease or health condition
F2_B. Drug response (medication)
F2_C. Carrier status (e.g. for pre-pregnancy planning)
F2_D. Ancestry
F2_E. Traits
F2_F. Participation in company-sponsored genomic research (outside of this study)

[NOTE: The following questions are for 23andMe ONLY]

{PRG: SHOW ME1 IF PRE_1 = 2 OTHERWISE GOTO FILTER BEFORE F3}

ME1. When you viewed your [DISPLAY PRE_1] results for disease risks, drug response and traits, did you notice the four-star rating system associated with the results?
1 Yes
0 No

{PRG: SHOW ME2 IF ME1 = 1 OTHERWISE GOTO F3}

ME2. How much importance did you place on the four-star rating system while viewing your results?

1 None
2 A little
3 Some
4 A lot

[NOTE: The following questions are for Pathway ONLY]

{PRG: SHOW PW1 IF PRE_1 = 1 OTHERWISE GO TO F3}

PW1. When you viewed your [DISPLAY PRE_1] results, did you notice whether the results were based off "validated" or "preliminary" research?

1 Yes
0 No

{PRG: SHOW PW2 IF PW1 = 1 OTHERWISE GOTO PW4}

PW2. How much importance did you place on whether the marker was validated or preliminary while viewing your results?

1 None
2 A Little
3 Some
4 A lot

{DESIGN: SHOW GRID F3-F4 AND F4B ON SAME PAGE}
{DESIGN: GRID F3-F4}
{PRG: F4B IS EXEMPT FROM SOFT PROMPT}

1 Not at all
2 A little
3 Somewhat
4 Very
5 Extremely

F3. In general, how satisfied are you regarding your decision to obtain personal genomic testing?

F4. In general, how valuable were your {DISPLAY PRE_1} results?

F4b. Please explain why you think the personal genomic testing experience was valuable or was not valuable. *(Optional)*

[OPEN END RESPONSE]

{PRG: F4C IS EXEMPT FROM SOFT PROMPT}
{DEISGN: SHOW GRID F4A AND F4C ON SAME PAGE}
{DESIGN: GRID F4A-F4C}

1 Not at all
2 A little
3 Somewhat
4 Very
5 Extremely

F4a. How important is it for you to get your personal test results updated as genomic technologies improve?

F4c. How useful do you anticipate that your results will be in your medical care five to ten years from now?

F4d. How might you envision using your genetic information in the future? *(Optional)*
[OPEN ENDED RESPONSE]

{DESIGN: GRID F5a-F5d}

F5. To what extent do you agree or disagree with the following statements?

1 Strongly Disagree
2 Somewhat Disagree
3 Neither Agree nor Disagree
4 Somewhat Agree
5 Strongly Agree

F5b. The information I received from {DISPLAY PRE_1} has influenced how I will manage my health in the future.
F5c. Having personal genomic testing made me feel like I have more control over my health.
F5d. Having personal genomic testing helped me to get a better perspective on my health status.
F5e. What I learned from my personal genomic testing can help reduce my chances of getting sick.

{DESIGN: GRID F6a-F6p}

F6. To what extent do you agree or disagree with the following statements?

1 Strongly Disagree
2 Somewhat Disagree
3 Neither Agree nor Disagree
4 Somewhat Agree
5 Strongly Agree

F6a. I am confident in the quality and accuracy of my {DISPLAY PRE_1} results
F6c. The education materials provided were adequate
F6d. I feel that I got what I paid for
F6h. I would have utilized in-person genetic counseling services had they been available
F6i. I learned something to improve my health that I didn't know before
F6j. I am disappointed that my {DISPLAY PRE_1} results did not tell me more information
F6k. I found my {DISPLAY PRE_1} results interesting
F6l. I found the information from {DISPLAY PRE_1} to be fun and entertaining

---

{PRG: GRID F6_2B – F6_2P}
{PRG: RANDOMIZE BLOCK F6_2B – F6_2P}

F6_2. To what extent do you agree or disagree with the following statements?

1 Strongly Disagree
2 Somewhat Disagree
3 Neither Agree nor Disagree
4 Somewhat Agree
5 Strongly Agree

F6_2b. It is important to me that my genetic information is kept private
F6_2e. I think that health insurance should cover personal genomic testing
F6_2f. I think genetic information should be part of a standard medical record
F2_6g. Genetic tests should be available more widely (e.g. test kits at drugstores)
F6_2m. I think that parents should be able to get {DISPLAY PRE_1} results for their children if they want to
F6_2n. I think the government should put more effort into regulating personal genomic testing
F6_2o. Tests like these should only be available to people through their doctor
F6_2p. I think people have a right to access their own genetic information without going through a medical professional.

{DESIGN: GRID F7a-F7e}

F7. Please reflect on the decision that you made about pursuing personal genomic testing.

To what extent do you agree or disagree with the following statements?

1 Strongly Disagree
2 Somewhat Disagree
3 Neither Agree nor Disagree
4 Somewhat Agree
5 Strongly Agree

F7a. It was the right decision
F7b. I regret the decision that I made
F7c. I would make the same decision
F7d. The decision did me a lot of harm
F7e. The decision was a wise one

{PRG: NO SOFT PROMPT FOR F8}

F8. Thinking back to your initial decision to seek personal genomic services, is there anything that you wish you had known then, before you sought the service? If so, please explain.

[OPEN END RESPONSE]

{PRG NOTE: SECTION HEADER: "Health, Behaviors and Insurance"}

{PRG: G1 SELECT ALL THAT APPLY}

G1. Have you made any of the following health or wellness changes that were specifically motivated by your {DISPLAY PRE_1} results?
(Select all that apply)

1 Diet
2 Exercise
3 Use of vitamins/ herbal supplements
4 Use of aspirin every day
5 Other *(please specify):* [TEXT RESPONSE]
6 I have not made any health or wellness changes that were motivated by my {DISPLAY PRE_1} results

---

{PRG: SHOW IF G1=1; OTHERWISE SKIP TO FILTER BEFORE G1_2}
{DESIGN: GRID G1_1a-G1_1d}

G1_1. Was this health or wellness change relating to your **diet**…

1 Yes
0 No

G1_1a. Self-motivated?
G1_1b. Recommended by a healthcare professional?
G1_1c. Recommended by [DISPLAY PRE_1]?

G1_1e. Please describe this change in greater detail:

[OPEN END RESPONSE]

{PRG: SHOW IF G1=2; OTHERWISE SKIP TO FILTER BEFORE G1_3}
{DESIGN: GRID G1_2a-G1_2d}

G1_2. Was this health or wellness change relating to **exercise**…

1 Yes
0 No

G1_2a. Self-motivated?
G1_2b. Recommended by a healthcare professional?
G1_2c. Recommended by [DISPLAY PRE_1]?

G1_2e. Please describe this change in greater detail:

[OPEN END RESPONSE]

{PRG: SHOW IF G1=3; OTHERWISE SKIP TO FILTER BEFORE G1_4}
{DESIGN: GRID G1_3a-G1_3d}

G1_3. Was this health or wellness change relating to use of **vitamins/herbal supplements**…

1 Yes
0 No

G1_3a. Self-motivated?
G1_3b. Recommended by a healthcare professional?
G1_3c. Recommended by [DISPLAY PRE_1]?

G1_3e. Please describe this change in greater detail:

[OPEN END RESPONSE]

{PRG: SHOW IF G1=4; OTHERWISE SKIP TO G2}
{DESIGN: GRID G1_4a-G1_4d}

G1_4. Was this health or wellness change relating to **taking aspirin every day**…

1 Yes
0 No

G1_4a. Self-motivated?
G1_4b. Recommended by a healthcare professional?
G1_4c. Recommended by [DISPLAY PRE_1]?

{NO SOFT PROMPT}
G1_4e. Please describe this change in greater detail: *(Optional)*

[OPEN END RESPONSE]

{SHOW G1_5 IF G1.5TEXT IS ENTERED}

G1_5. Was this health or wellness change relating to {RESPONSE:G1.5TEXT}

1 Yes
0 No

G1_5a. Self-motivated?
G1_5b. Recommended by a healthcare professional?
G1_5c. Recommended by [DISPLAY PRE_1]?

G2. Do you think you will use your {DISPLAY PRE_1} results to guide your future use of medication?

1 Yes
0 No
99 Don’t know

{DESIGN: GRID G3a-G3e}

G3. Have you made changes to any of the following types of medications and supplements as a result of seeing your {DISPLAY PRE_1} results?

1 Yes
0 No

G3a. Prescription medications
G3b. Non-prescription medications
G3c. Nutritional supplements
G3d. Alternative medicines
G3e. Other

{PRG: SHOW G3a_1- G3a_3 IF G3a=1; OTHERWISE SKIP TO FILTER BEFORE G3b_1}
{DESIGN: SAME SCREEN G3a_1- G3a_3}
{PRG: G3a_3 IS EXEMPT FROM SOFT PROMPT}

G3a_1. What changes did you make to your **prescription** medications? (*Please select all that apply.)*

1 Stopped taking a medication you were taking
2 Started taking a new medication
3 Lowered the dosage of a medication you were taking
4 Raised the dosage of a medication you were taking
5 Switched from one medication to another medication

G3a_2. Did you consult with a medical professional prior to making these changes to your prescription medications?

1 Yes
0 No

G3a_3. Can you tell us a bit more about why your {DISPLAY PRE_1} results prompted you to make these changes to your prescription medications? *(Optional)*

[OPEN END RESPONSE]

{PRG: SHOW G3b_1- G3b_3 IF G3b=1; OTHERWISE SKIP TO FILTER BEFORE G3c_1}
{DESIGN: SAME SCREEN G3b_1- G3b_3}
{PRG: G3B_3 IS EXEMPT FROM SOFT PROMPT}

G3b_1. What changes did you make to your **non-prescription** medications? (*Please select all that apply.)*

1 Stopped taking a medication you were taking
2 Started taking a new medication
3 Lowered the dosage of a medication you were taking
4 Raised the dosage of a medication you were taking
5 Switched from one medication to another medication

G3b_2. Did you consult with a medical professional prior to making these changes to your non-prescription medications?

1 Yes
0 No

G3b_3. Can you tell us a bit more about why your {DISPLAY PRE_1} results prompted you to make these changes to your non-prescription medications? *(Optional)*

[OPEN END RESPONSE]

{PRG: SHOW G3c_1- G3c_3 IF G3c=1; OTHERWISE SKIP TO FILTER BEFORE G3d_1}
{DESIGN: SAME SCREEN G3c_1- G3c_3}
{PRG: G3C_3 IS EXEMPT FROM SOFT PROMPT}

G3c_1. What changes did you make to your **nutritional supplements**? (*Please select all that apply.)*

1 Stopped taking a supplement you were taking
2 Started taking a new supplement
3 Lowered the dosage of a supplement you were taking
4 Raised the dosage of a supplement you were taking
5 Switched from one supplement to another supplement

G3c_2. Did you consult with a medical professional prior to making these changes to your nutritional supplements?

1 Yes
0 No

G3c_3. Can you tell us a bit more about why your {DISPLAY PRE_1} results prompted you to make these changes to your nutritional supplements? *(Optional)*

[OPEN END RESPONSE]

{PRG: SHOW G3d_1- G3d_3 IF G3d=1; OTHERWISE SKIP TO FILTER BEFORE G3e_1}
{DESIGN: SAME SCREEN G3d_1- G3d_3}
{PRG: G3D_3 IS EXEMPT FROM SOFT PROMPT}

G3d_1. What changes did you make to your **alternative medicines**? (*Please select all that apply.)*

1 Stopped taking a medication you were taking
2 Started taking a new medication
3 Lowered the dosage of a medication you were taking
4 Raised the dosage of a medication you were taking
5 Switched from one medication to another medication

G3d_2. Did you consult with a medical professional prior to making these changes to your alternative medicines?

1 Yes
0 No

G3d_3. Can you tell us a bit more about why your {DISPLAY PRE_1} results prompted you to make these changes to your alternative medicines? *(Optional)*

[OPEN END RESPONSE]

{PRG: SHOW G3e_0- G3e_3 IF G3e=1; OTHERWISE SKIP TO H1}
{DESIGN: SAME SCREEN G3e_0- G3e_3}
{PRG: G3E_3 IS EXEMPT FROM SOFT PROMPT}

G3e_0. What **other** medications and supplements have you made changes to?

[OPEN END RESPONSE]

G3e_1. What changes did you make to the other medications and supplements that you listed? (*Please select all that apply.)*

1 Stopped taking a medication/supplement you were taking
2 Started taking a new medication/supplement
3 Lowered the dosage of a medication/supplement you were taking
4 Raised the dosage of a medication/supplement you were taking
5 Switched from one medication/supplement to another

G3e_2. Did you consult with a medical professional prior to making these changes to the other medications and supplements that you listed?

1 Yes
0 No

G3e_3. Can you tell us a bit more about why your {DISPLAY PRE_1} results prompted you to make these changes to the other medications and supplements that you listed? *(Optional)*

[OPEN END RESPONSE]

{DESIGN: GRID H1-H2}

0 None
1 1 or less
2 2
3 3
4 4
5 5 or more

H1. On a typical day, how many servings of **fruit** do you eat? *(A serving size equals 1 piece of fruit or melon wedge, 3/4 cup of 100% juice, 1/2 cup canned fruit, or 1/4 cup dried fruit)*

H2. On a typical day, how many servings of **vegetables** do you eat? *(A serving size equals 1/2 cup chopped raw or cooked vegetables, 1 cup leafy raw vegetables, or 3/4 cup 100% vegetable juice.)*

{PRG: DISPLAY RESPONSE OPTIONS HORIZONTALLY ACROSS THE SCREEN}

J1. What is your height?

J1a. [NUMERIC RANGE 4-7] Feet J1b. [NUMERIC RESPONSE, 0-11] Inches

---

J2. What is your weight?

[NUMERIC RANGE 70-400] Pounds

{DESIGN: GRID J3A – J3C}

J3. As a result of seeing your {DISPLAY PRE_1} results, did you:

1 Yes
0 No

J3a. Join a gym or other exercise program?
J3b. Start exercising on your own?
J3C. Increase your amount of exercise?

{PRG: J4a_1 AS CHECKBOX}
{PRG: RESPONDENT CANNOT SELECT J4a_1 AND ENTER A RESPONSE IN J4a}

J4_TEXT. The next questions are about physical activities that you may do in your **leisure** time.

J4. How many days per week do you do **vigorous leisure-time** physical activities for **at least 10 minutes** that cause **heavy** sweating or **large** increases in breathing or heart rate?

J4a. [NUMERIC RANGE, ALLOW 0-7] days per week

{PRG: SHOW IF J4a>0; OTHERWISE SKIP TO J5}

J4c. About how long do you do these vigorous leisure-time physical activities each time?

[NUMERIC RANGE, ALLOW 1-240] minutes

{PRG: J5a_1 AS CHECKBOX}
{PRG: RESPONDENT CANNOT SELECT J5a_1 AND ENTER A RESPONSE IN J5a}

J5. How many days per week do you do **light or moderate leisure-time** physical activities for **at least 10 minutes** that cause **only light** sweating or a **slight** to **moderate** increase in breathing or heart rate?

J5a. [NUMERIC RANGE; ALLOW 0-7] days per week

{PRG: SHOW IF J5a>0; OTHERWISE SKIP TO J6}

J5c. About how long do you do these light or moderate leisure-time physical activities each time?

[NUMERIC RANGE, ALLOW 1-240] minutes

{PRG: J6a_1 AS CHECKBOX}
{PRG: RESPONDENT CANNOT SELECT J6a_1 AND ENTER A RESPONSE IN J6a}

J6. How many days per week do you do **leisure-time** physical activities **specifically designed to strengthen your muscles** such as lifting weights or doing calisthenics?
(Include all such activities even if you have mentioned them before.)

J6a. [NUMERIC RANGE, ALLOW 0-7] days per week

{PRG: SHOW IF J6a>0; OTHERWISE SKIP TO J7}

J6c. About how long do you do these strengthening leisure-time physical activities each time?

[NUMERIC RANGE, 1-240] minutes

{PRG: SHOW J7 IF J4a >= 3; OTHERWISE SKIP TO FILTER BEFORE J8}

J7. How long have you done **vigorous leisure-time** physical activities 3 or more times a week?

1 I have been doing them for **more** than 6 months
2 I have been doing them for **less** than 6 months

{PRG: SHOW J8 IF J4a < 3; OTHERWISE SKIP TO K1}

J8. Do you intend to increase your level of physical activity?

1 I intend to in the next 30 days
2 I intend to in the next 6 months
3 I do **not** intend to in the next 6 months

K1. Have you smoked at least 100 cigarettes in your entire life?

1 Yes
0 No

{PRG: SHOW IF K1=1; OTHERWISE SKIP TO M1}

K2. Do you **now** smoke cigarettes not at all, some days, or every day?

1 Not at all
2 Some days
3 Every day

{PRG: SHOW IF K2=2 OR 3; OTHERWISE SKIP TO M1}

K2a. How many cigarettes per day do you smoke?

1 10 or less
2 11-20
3 21-30
4 31 or more

M1. Have you made any changes to your insurance coverage (i.e., health, life, long-term care, disability) that were related to your {DISPLAY PRE_1} results?

1 Yes
0 No

{PRG: SHOW M1a IF M1=1; OTHERWISE SKIP TO M1b}
{PRG: M1a SELECT ALL THAT APPLY}

M1a. To which insurance coverage did you make changes? *(Select all that apply)*

1 Health insurance
2 Life insurance
3 Long-term care insurance
4 Disability

{PRG: SHOW IF M1a=1; OTHERWISE SKIP TO FILTER BEFORE M1a_2}
{PRG: M1a_1 SELECT ALL THAT APPLY}

M1a_1. What changes did you make to your health insurance coverage? *(Select all that apply)*

1 Dropped coverage
2 Decreased existing coverage
3 Added coverage
4 Increased existing coverage
5 Other *(Please specify)*: [TEXT RESPONSE]

{PRG: SHOW IF M1a=2; OTHERWISE SKIP TO FILTER BEFORE M1a_3}
{PRG: M1a_2 SELECT ALL THAT APPLY}

M1a_2. What changes did you make to your life insurance coverage? *(Select all that apply)*

1 Dropped coverage
2 Decreased existing coverage
3 Added coverage
4 Increased existing coverage
5 Other *(Please specify)*: [TEXT RESPONSE]

{PRG: SHOW IF M1a=3; OTHERWISE SKIP TO FILTER BEFORE M1a_4}
{PRG: M1a_3 SELECT ALL THAT APPLY}

M1a_3. What changes did you make to your long-term care insurance coverage? *(Select all that apply)*

1 Dropped coverage
2 Decreased existing coverage
3 Added coverage
4 Increased existing coverage
5 Other *(Please specify)*: [TEXT RESPONSE]

{PRG: SHOW IF M1a=4; OTHERWISE SKIP TO FILTER BEFORE M1b}
{PRG: M1a_4 SELECT ALL THAT APPLY}

M1a_4. What changes did you make to your disability insurance coverage? *(Select all that apply)*

1 Dropped coverage
2 Decreased existing coverage
3 Added coverage
4 Increased existing coverage
5 Other *(please explain)*: [TEXT RESPONSE]

M1b. Do you **plan to make** any {PRG: IF M1=1 DISPLAY “other”} changes to your insurance coverage that are related to your {DISPLAY PRE_1} results?

1 Yes
0 No

{PRG: SHOW IF M1b=1; OTHERWISE SKIP TO M2}

M1b_1. What changes do you plan to make to your insurance coverage? (select all that apply)

1 Drop coverage
2 Decrease existing coverage
3 Add coverage
4 Increase existing coverage
5 Other *(please explain)*: [TEXT RESPONSE]

M2. Have you made any changes to your financial or retirement plans that were related to your {DISPLAY PRE_1} results?

1 Yes
0 No

{PRG: SHOW IF M2=1; OTHERWISE SKIP TO FILTER BEFORE M2b}
{PRG: M2A IS EXEMPT FROM SOFT PROMPT}

M2a. What changes have you made to your financial or retirement plans?

[OPEN END RESPONSE]

M2b. Do you **plan to make** any {PRG: IF M2=1 DISPLAY “other”} changes to your financial or retirement plans that are related to your {DISPLAY PRE_1} results?

1 Yes
0 No

{PRG: SHOW M2b_1 IF M2b =1, OTHERWISE SKIP TO M3}
{PRG: M2B_1 IS EXEMPT FROM SOFT PROMPT}

M2b_1. What changes do you plan to make to your financial or retirement plans?

[OPEN END RESPONSE]

M3. As a result of learning your genetic information from {DISPLAY PRE_1}, have you made any other changes related to advanced planning (e.g., will, advance directives, power of attorney)?

1 Yes
0 No

{PRG: SHOW IF M3=1; OTHERWISE SKIP TO FILTER BEFORE M3b}
{PRG: M3A IS EXEMPT FROM SOFT PROMPT}

M3a. What changes have you made related to advanced planning?

[OPEN END RESPONSE]

M3b. Do you **plan to make** any {PRG: IF M3=1 DISPLAY “other”} changes to your advanced planning that are related to your {DISPLAY PRE_1} results?

1 Yes
0 No

{PRG: SHOW IF M3b=1; OTHERWISE SKIP TO M4}
{PRG: M3B_1 IS EXEMPT FROM SOFT PROMPT}

M3b_1. What changes do you plan to make to your advanced planning?

[OPEN END RESPONSE]

{PRG NOTE: SECTION HEADER: "Genetics and Sharing Your Results"}

M4. Please indicate whether you think the following statements are true or false.

1 True
0 False

M4a. Healthy parents can have a child with an inherited disease
M4b. If your close relatives have diabetes or heart disease, you are more likely to develop these conditions
M4c. Some genetic disorders occur more often within particular ethnic groups
M4d. Most genetic disorders are caused by only a single gene
M4e. Once a genetic marker for a disorder is identified in a person, the disorder can usually be prevented or cured
M4f. A disease is only genetically determined if more than one family member is affected
M4g. Some of the genetic disorders occur later in adult life
M4h. A healthy lifestyle can prevent or lessen the negative consequences of genetic predispositions to some diseases
M4i. The environment has little or no effect on how genes contribute to disease

---

{PRG: M5 IN GRID}

M5. How much do you agree or disagree with the following statements?

1 Strongly Disagree
2 Disagree
3 Somewhat Disagree
4 Neither Agree nor Disagree
5 Somewhat Agree
6 Agree
7 Strongly Agree

M5a. I am confident in my ability to understand information about genetics.
M5b. I am able to understand information about how my DNA can affect my health.
M5c. I have a good idea about how genetics may influence risk for disease generally.
M5d. I have a good idea about how my own genetic make-up might affect my risk for diseases.
M5e. I am able to explain to others how genetic variants affect one's health.

You are almost done with the survey! Here is the final set of questions.
{PRG: S0 IS EXEMPT FROM SOFT PROMPT}

S0. In general, do you think your yourself as…

1 Extremely liberal
2 Liberal
3 Slightly liberal
4 Moderate, middle of the road
5 Slightly conservative
6 Conservative
7 Extremely conservative

S1. Which social networking sites do you participate in?
(Select all that apply)

1 Twitter
2 Facebook
4 LinkedIn
5 PatientsLikeMe
6 23andMe's discussion forum
7 Other *(Please specify)* [TEXT RESPONSE]
8 I do not participate in any social networking sites

{SHOW S1a IF S1.8 IS SELECTED}

S1a. How often do you log into any social networking sites?

1 More than a few times a day
2 A few times a day
3 Once a day
4 Once every few days
5 About once a week
6 Only occasionally

{PRG: GRID S2_1A – S2_1C}

S2_1. Have you ever done any of the following:

1 Yes
0 No

S2_1a Gone online to connect with others who might have health concerns similar to yours?
S2_1d Gone online to find information provided by others who have health concerns similar to yours?
S2_1b Posted about health or medical matters on social networking sites such as Facebook, Twitter, PatientsLikeMe, or 23andMe's discussion forum?
S2_1c Shared some of your {DISPLAY PRE_1} results on any social networking sites?

{PRG: SHOW S2a IF S2_1A= 1}

S2a. What sites or other online resources have you used to find others who might have health concerns similar to yours?

[OPEN END RESPONSE]

{PRG: SHOW S2b IF S2_1B= 1}

S2b. On which social networking sites have you posted about health or medical matters?
(Select all that apply)

1 Twitter
2 Facebook
4 LinkedIn
5 PatientsLikeMe
6 23andMe's discussion forum
7 Other (Please specify) [TEXT RESPONSE]

{PRG: SHOW S2c IF S2_3= 1}

S2c. How many people did you meet or reconnect with by sharing your {DISPLAY PRE_1} results on social networking sites?

0 No one
1 1
2 <5
3 5-10
4 >10

S2D. May we contact you about opportunities to participate in future research (for example, other surveys or interviews)?

1 Yes
0 No

S2E. May we contact you about opportunities to speak with the media about your experience of participating in the PGen Study?

1 Yes
0 No

{PRG: SHOW S2F IF SUM S2D AND S2E > 0; OTHERWISE GOTO S2G}

S2F. Thank you! Please enter your mailing address and phone number so that we may contact you in future.

Mailing Address: [TEXT RESPONSE]
City: [TEXT RESPONSE}
State: [TEXT RESPONSE]
Zip Code: [NUMERICAL RESPONSE 00000-99999]
Phone number: [NUMERICAL RESPONSE]

{PRG: S2G IS EXEMPT FROM SOFT PROMPT}

S2G. Is there anything else you'd like to share about your experience of participating in the PGen Study? *(Optional)*
[OPEN ENDED RESPONSE]
