# Supplementary material for: Design, methods, and participant characteristics of the Impact of Personal Genomics (PGen) Study, a prospective cohort study of direct-to-consumer personal genomic testing customers
Source: Genome Med. 2014 Dec 3;6(12):96. doi: 10.1186/s13073-014-0096-0 (PMC4256737; doi:10.1186/s13073-014-0096-0)
Supplement: Additional file 4: — 6 month follow-up non-responder PGen Study survey. [file 13073_2014_96_MOESM4_ESM.pdf]

S10028: PGen 6-month NR Survey

Short URL to direct towards production survey	https://www.ssgresearch.com/pgennr
Support email address to include in header	pgen@ssgresearch.com
Support phone number to include in header (if needed)	

Logo to use if other than SSG logo
Please list network location of other logo to use:

Mandatoriness (check the appropriate setting)

	All questions are optional unless otherwise noted
x	All questions are optional with a soft prompt included if no answer is provided [OTHER SPECIFY FIELDS SHOULD ALSO HAVE SOFT PROMPTS]
	All questions are mandatory

Please provide text to use for Mandatoriness prompt if being used (Default text to use is provided below):
General:

We noticed that you did not answer a question on the previous page. It is important to us that we get a complete set of responses from you. Please return to the previous page by clicking "Previous" and select an answer for each question. If you would rather not select an answer, you may instead continue to the next page by clicking "Next."

Other specify:

You selected 'Other' but did not specify your answer. Please return to the last question by clicking “Previous” and type in your specific answer. If you would rather not specify an answer, you may instead continue to the next page by clicking “Next.”

Header Sections (if being used)
NONE

Survey Title to appear in header (appears above the section header bar)

The Impact of Personal Genomics (PGen) Study

Welcome Page text (please modify the following as needed)

Welcome to the Impact of Personal Genomics (PGen) Study!

This is the last of three surveys for this study.

This very short version of the survey will take only 3-5 minutes of your time.

Please enter your Study ID as provided to you in the letter or email you received, then click Start Survey to begin!

Resume Page text (please modify the following as needed)

Thank you for returning to the survey. Please click “resume” to begin where you last left off…

End Page Text

S10028: PGen 6-month NR Survey

Thank you for your participation! This concludes the last survey for this research study.

For information about the PGen Study, please refer to the study website at:

http://www.genomes2people.org/g2p/pgen/

Results of the PGen Study will be posted on the study website as they become available.

Within the next 2 weeks, you will receive a $20 Amazon.com gift certificate via email.

You may now close your browser.

Survey Title appearing in browser window

PGen Study

GENERAL PROGRAMMING NOTES

All emphasis should be programmed in black, all caps text instead of lowercase blue text.

Preloads

PRE_1 Genetics Company

1 Pathway Genomics
2 23andMe

A1. Have you discussed your {RESPONSE:PRE_1} results with anyone?

1 Yes
0 No

{PRG: SHOW A2 IF A1=1}
{PRG: Select all that apply}

A2. With whom did you discuss your {RESPONSE:PRE_1} results? *(Select all that apply)*

1 Family members
2 Friends
3 Co-workers/colleagues
4 Primary care provider
5 Genetics specialist (e.g., genetic counselor, clinical geneticist)
6 Other medical professional
7 Contacts on social networking services (e.g., Facebook, MySpace, Twitter)
8 Contacts on health-or disease-based social networking services (e.g., Patients Like Me, 23andMe's discussion forum, Cure Together, disease-specific patient networks)

A6. Have your {RESPONSE:PRE_1} results prompted you to make an appointment with a medical professional(s)?

2 Yes, I have already made an appointment
1 I plan to make an appointment
0 No, I do not plan to make an appointment

B2. As a result of seeing your genetic information, have you had any tests, medical exams, or procedures?

1 Yes
0 No

{SHOW B2a IF B2=1, OTHERWISE GO TO G1}
{PRG: B2a CHECK ALL THAT APPLY}

B2a. What kind of tests, medical exams, or procedures did you have as a result of seeing your genetic information? *(Select all that apply)*

1 Genetic test(s) to confirm a variant such as BRCA1, BRCA2, Factor II or Factor V, or other carrier status variants, such as cystic fibrosis
2 Medical exams or procedures to screen or test for a specific disease/condition
3 Whole body scan
4 Other

{PRG: CHECK ALL THAT APPLY}
{PRG: G1.6 IS MUTUALLY EXCLUSIVE}

G1. Have you made any of the following health or wellness changes that were specifically motivated by your results? *(Select all that apply)*

1 Diet
2 Exercise
3 Use of vitamins/herbal supplements
4 Use of aspirin every day
5 Other [SPECIFY]
6 None

M1. Have you made any changes to your insurance coverage (i.e. health, life, long-term care, disability) that were related to your results?

1 Yes
0 No

{SHOW M1a IF M1=1}
{PRG: SELECT ALL THAT APPLY}

M1a. To which insurance coverage did you make changes? *(Select all that apply)*

1 Health insurance
2 Life insurance
3 Long-term care insurance
4 Disability

M1b. Do you **plan to make** any {PRG: IF M1=1 DISPLAY "other"} changes to your insurance coverage that are related to your {RESPONSE:PRE_1} results?

1 Yes
0 No

F3. In general, how satisfied are you regarding your decision to obtain personal genomic testing?

1 Not at all
2 A little
3 Somewhat
4 Very
5 Extremely

F4. In general, how valuable were your {RESPONSE:PRE_1} results?

1 Not at all
2 A little
3 Somewhat
4 Very
5 Extremely

{PRG: SHOW D3 and D3b ON SAME PAGE}
{PRG: D3 and D3b COMMENTARY }

D3. Which result or results were most important to you?
[OPEN ENDED RESPONSE]

D3b. Please explain why this result or results were most important to you.
[OPEN ENDED RESPONSE]

Q1. Do you recall receiving an email invitation over the past few months for the final PGen Study survey?

1 Yes
0 No
2 Don't know

{SHOW Q2 IF Q1=1}
{PRG: SELECT ALL THAT APPLY}

Q2. Why did you not complete the final PGen Study survey when you received the email invitation? *(Select all that apply.)*

1 Forgot
2 Not interested
3 No time
4 Too long
5 Too complex
6 I didn't trust that my data would be kept confidential
7 Topic concerns

8 Technical difficulties with the website
9 I have already completed other questionnaires and didn't want to do another one
10 It wasn't worth the $20
11 Difficulties/problems with previous surveys for this study
12 I didn't realize that I was being asked to participate again
13 No particular reason
14 Other [Please specify]

Q6MONTH. Once you answer this final question, you will be finished with the short version of the survey, and you will receive a $20 Amazon.com gift card within 2 weeks. Many people who complete this shortened survey ask to continue and finish the full survey. You will have the opportunity to do this, if you wish. Whether or not you complete the full survey will not impact the receipt of your gift card.

Would you like to complete the full survey now?

1 Yes (you will be automatically re-directed to the full survey)
0 No

{PRG: IF Q6MONTH = 0 JUMP TO END AND SUBMIT}
{PRG: IF Q6MONTH = 1, JUMP TO END AND SUBMIT AND REDIRECT TO FULL SURVEY}
